# An axisymmetric shock breakout indicated by prompt polarized emission from the type II supernova 2024ggi

**DOI:** 10.1126/sciadv.adx2925

**Published:** 2025-11-12

**Authors:** Yi Yang, Xudong Wen, Lifan Wang, Dietrich Baade, J. Craig Wheeler, Alexei V. Filippenko, Avishay Gal-Yam, Justyn Maund, Steve Schulze, Xiaofeng Wang, Chris Ashall, Mattia Bulla, Aleksandar Cikota, He Gao, Peter Hoeflich, Gaici Li, Divya Mishra, Ferdinando Patat, Kishore C. Patra, Sergiy S. Vasylyev, Shengyu Yan

**Affiliations:** ^1^ Department of Physics, Tsinghua University, Qinghua Yuan, Beijing 100084, China.; ^2^ School of Physics and Astronomy, Beijing Normal University, Beijing 100875, China.; ^3^ Department of Physics and Astronomy, Texas A&M University, 4242 TAMU, College Station, TX 77843, USA.; ^4^ George P. and Cynthia Woods Mitchell Institute for Fundamental Physics and Astronomy, Texas A&M University, 4242 TAMU, College Station, TX 77843, USA.; ^5^ European Organisation for Astronomical Research in the Southern Hemisphere (ESO), Karl-Schwarzschild-Str. 2, 85748 Garching b. München, Germany.; ^6^ University of Texas at Austin, 1 University Station C1400, Austin, TX 78712-0259, USA.; ^7^ Department of Astronomy, University of California, Berkeley, CA 94720-3411, USA.; ^8^ Hagler Institute for Advanced Study, Texas A&M University, 3572 TAMU, College Station, TX 77843, USA.; ^9^ Department of Particle Physics and Astrophysics, Weizmann Institute of Science, Rehovot, Israel.; ^10^ Department of Physics, Royal Holloway, University of London, Egham Hill, Egham, TW20 0EX, UK.; ^11^ Center for Interdisciplinary Exploration and Research in Astrophysics (CIERA), Northwestern University, 1800 Sherman Ave, Evanston, IL 60201, USA.; ^12^ Department of Physics, Virginia Tech, 850 West Campus Drive, Blacksburg, VA 24061, USA.; ^13^ Institute for Astronomy, University of Hawai’i at Manoa, 2680 Woodlawn Dr., Hawai’i, HI 96822, USA.; ^14^ Department of Physics and Earth Science, University of Ferrara, via Saragat 1, I-44122 Ferrara, Italy.; ^15^ INFN, Sezione di Ferrara, via Saragat 1, I-44122 Ferrara, Italy.; ^16^ INAF, Osservatorio Astronomico d’Abruzzo, via Mentore Maggini snc, 64100 Teramo, Italy.; ^17^ Gemini Observatory/NSF’s NOIRLab, Casilla 603, La Serena, Chile.; ^18^ Institute for Frontier in Astronomy and Astrophysics, Beijing Normal University, Beijing 102206, China.; ^19^ Department of Physics, Florida State University, Tallahassee, FL 32306, USA.; ^20^ Department of Astronomy and Astrophysics, University of California, Santa Cruz, CA 95064, USA.

## Abstract

The death of massive stars is triggered by an infall-induced bounce shock that disrupts the star. How such a shock is launched and propagates through the star is a decade-long puzzle. Some models assume that the shock can be reenergized by absorbing neutrinos, leading to highly aspherical explosions. Other models involve jet-powered shocks that lead to bipolar explosions reflected in the geometry of the shock-breakout emission. We report measurement of the geometry of the shock breakout through unprecedentedly early spectropolarimetry of the nearby type II supernova 2024ggi starting ~1.2 days after the explosion. The measurement indicates a well-defined symmetry axis of the shock breakout, which is also shared by the hydrogen-rich envelope that emerged after the circumstellar matter was engulfed by the ejecta, revealing a persisting and prominent symmetry axis throughout the explosion. These findings suggest that the physical mechanism driving the explosion of massive stars manifests a well-defined axial symmetry and acts on large scales.

## Teaser

Prompt polarized emissions reveal that the terminating explosion of a massive star is axisymmetric.

## INTRODUCTION

“Since the beginning of physics, symmetry considerations have provided us with an extremely powerful and useful tool in our effort to understand nature (*1*).” The geometry of a supernova (SN) explosion, which has been found aspherical, provides fundamental information on stellar evolution and the physical processes leading to these cosmic fireworks (*2*). Iron-core collapses of massive stars in the mass range of 8 to 20 solar masses (*3*, *4*) are the dominant stellar explosions in the nearby universe (*5*).

Neutrino-driven models of core-collapse supernovae (CCSNe) have only become successful in recent years thanks to three-dimensional (3D) simulations. In particular, the rebounce shockwave may stall to accretion toward certain directions, while the accretion of in-falling matter onto the proto neutron star and neutrino energy deposition is continuous in other directions. Such a neutrino-driven explosion would result in a break of spherical symmetry (*6*–*8*). Nevertheless, explaining the details about the generation of the shock waves during the collapse of the stellar core and the energy transportation via a burst of neutrinos to produce an explosion remains a challenge. Alternative models include the deposition of energy in the stellar envelope through mechanisms such as magnetorotational processes during the formation of the protoneutron star. This process, in which the progenitor iron core exhibits a short rotation period of ≲10 s (*9*, *10*), may launch moderately relativistic jets into the outer core and the stellar envelope (*11*–*16*). Engines driving core-collapse explosions may follow well-defined global asphericities of the progenitor systems as hinted at by the observational evidence of SN remnants (*17*, *18*) and pulsar kicks (*19*–*23*). Bipolar/jet-driven models compatible with these observations have been proposed (*12*, *13*, *24*–*26*). Explosion models adopting pure neutrino heating within the spherically symmetric scheme (*27*–*29*) or driven by small-scale instabilities (*30*), on the other hand, are expected to be amorphous or exhibit no symmetry axis. Recent 3D radiation-hydrodynamic simulations also illustrate that microscopic neutrino physics details in the early seconds can determine the large-scale ejecta structure that is preserved for days (*31*). Modeling of the blueward color evolution of SN 2023ixf, recorded within a few hours after the first light, infers an inhomogeneous emergence of the shock from the exploding star enshrouded by circumstellar matter (CSM) that started from where the opacity yields the smallest (*32*). The critical link between the shock breakout and the explosion mechanism that drives the expansion of the ejecta may be facilitated by comparing their geometries. Whether the former lines up with that of the SN ejecta and any explosion fingerprints left toward the core-collapse center would thus provide a powerful probe of the explosion physics.

Extremely early spectropolarimetry, taken within about 1 day after shock breakout, offers a unique opportunity to observe how the shock emerges on the surface of the exploding star and interacts with any surrounding CSM as evidenced by short-lived photoionized (“flash ionization”) spectral features (*33*–*39*). Because of the large distances of extragalactic supernovae (SNe), the regions concerned remain angularly unresolved, compressed to the radial velocity and time axes. Critical information about the 3D structure of the ejecta and their interaction with CSM is encoded in polarization spectra. Continuum polarization measures the deviations of the photosphere from spherical symmetry. Line polarization traces the distribution of elements in the SN ejecta projected onto the plane of the sky (*2*). Modulation of the polarization degree and position angle (PA) across a spectral feature probe the strength of departure from spherical symmetry and its orientation, respectively, delivering a low-resolution 3D map of the corresponding line-forming region (*2*, *40*, *41*). The acquisition of such a dataset close in time to shock breakout only became feasible recently thanks to the transient-alert stream produced by sub-day-cadence wide-field sky surveys, combined with rapid spectropolarimetric follow-up observations.

## RESULTS

### Spectropolarimetry of Supernova 2024ggi

SN 2024ggi was discovered as a transient with rapid intranight rise (*42*) in the spiral galaxy NGC 3621 at a distance of 7.24 ± 0.20 megaparsec (Mpc) (*43*) and was quickly classified as a young type II SN (*44*). The transient alert stream was produced by the “Asteroid Terrestrial-impact Last Alert System” (*45*). The proximity of SN 2024ggi provides a rare opportunity to investigate the pre-to-post-explosion properties of this CCSN in great detail. We initiated a spectropolarimetric time sequence of SN 2024ggi (see Table 1), starting at UTC 05:57 on 2024-04-12 (MJD 60412.248) following the immediate approval of the European Southern Observatory (ESO) Director’s Discretionary Program [ID 113.27R1; principal investigator (PI), Y.Y.]. The first epoch was carried out at ∼1.1 days after the discovery on MJD 60411.14 (*42*), which is an objective observation, and $1.22_{-0.05}^{+0.05}$ days after the estimated time of shock breakout on MJD $60411.03_{-0.05}^{+0.05}$ (*46*), which is model dependent. Throughout this paper, all phases are given relative to the time of the SN discovery. The observing campaign on SN 2024ggi harvested one of the two earliest spectropolarimetric datasets of any transient, the other was $1.39_{-0.02}^{+0.05}$ days after shock breakout (*32*) of SN 2023ixf (*47*). This rare early dataset enables us to measure the geometry of the shock breakout (see the “Spectropolarimetry of SN 2024ggi” section), which took place between days 0.7 and 1.2 as inferred from the early evolution of the ionization states of the CSM emission lines (*46*).

**Table 1.** Log of Very Large Telescope spectropolarimetry of SN 2024ggi.

Epoch	MJD	Phase (day)*	Grism	Exp time (s)^†^	Air mass	Grism	Exp time (s)^†^	Air mass
1	60412.246	1.1	300V	180 × 4 × 2	1.39	–	–	–
2	60413.144	2.0	300V	45 × 4 × 2	1.03	1200B	80 × 4 × 2	1.04
NA^‡^	60416.078	4.9	300V	90 × 4 × 2	1.02	1200B	240 × 4 × 2	1.01
3	60416.988	5.8	300V	90 × 4 × 2	1.22	1200B	240 × 4 × 2	1.16
4	60418.008	6.9	300V	50 × 4 × 2	1.13	–	–	–
5	60422.023	10.9	300V	70 × 4 × 2	1.07	1200R	130 × 4 × 2	1.03
6	60430.996	19.9	300V	75 × 4 × 2	1.08	1200R	140 × 4 × 2	1.05
7	60444.164	33.0	300V	40 × 4 × 2	1.41	–	–	–
8	60491.979	80.8	300V	65 × 4 × 2	1.16	1200R	130 × 4 × 2	1.21
9	60678.246	267.1	300V	480 × 4 × 2	1.26	–	–	–

* Relative to the estimated time of the shock breakout at MJD 60411.03.

† Observations carried out with two exposures each at four different half-wave–plate angles.

‡ Not applicable (NA) since dataset discarded due to poor seeing (∼4.8″).

Investigation of the geometry of the continuum and different spectral features can be facilitated by presenting spectropolarimetry on the normalized Stokes *Q-U* plane (*25*). A prominent axial symmetry of an electron-scattering structure leads to a wavelength-independent polarization PA of the continuum in the *Q-U* plane. For data points with different wavelengths, their distance from the origin (polarization degree *p*) varies owing to different physical properties across the photosphere (e.g., temperature, density, and composition), resulting in a range of optical depths and scattering efficiencies. Together, they form a straight line known as the dominant axis (*40*, *48*).

The polarization over certain spectral ranges can be decomposed into a component along the dominant axis ($P_{d}$) and another one along the orthogonal axis ($P_{o}$). The former captures the most dynamic range of the data (*40*). Its slope in the *Q-U* plane delivers the spatial orientation of the axial symmetry. For ejecta with rotational symmetry, the dominant and orthogonal axes measure the axial asphericity of the ejecta and the deviations from such a geometry, respectively. Therefore, for any wavelength range or spectral line of interest, a clear dominant axis would indicate a prominent axial symmetry of the associated opacity distribution. On the contrary, any clumpy, nonaxisymmetric structure will spread along the orthogonal axis, making the dominant axis less significant (*2*).

**Fig. 1.**
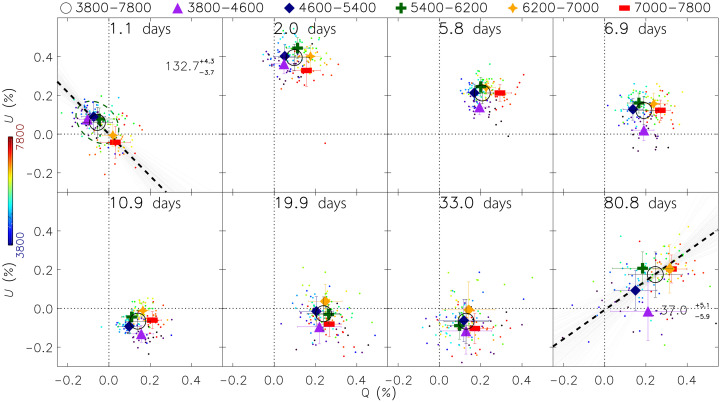
Temporal evolution of the polarization of SN 2024ggi after subtracting the ISP. In the top left and bottom right panels, the black dashed line shows the dominant axis determined from linear fits to the small data points (the PAs and uncertainties are labeled), which cover the wavelength range of 3800 to 7800 Å. The orientation of the dominant axis in degrees with uncertainties is indicated in the subpanels for days 1.1 and 80.8. A dashed ellipsoidal contour, whose major and minor axes respectively represent the 1σ dispersion about the dominant and orthogonal axis, is also presented. In each panel, different symbols mark the error-weighted mean polarization calculated over the wavelength ranges identified in the color bar. A marked change of the continuum polarization (from days 1.1 to 2.0) is followed by a gradual drift until a roughly stationary geometry is reached at day 10.9. This behavior is accompanied by a clockwise rotation of the distribution of the data points, revealing a large-scale transformation of the geometry as the CSM is swept up by the SN ejecta. Light gray lines in the top left and bottom right panels present the dominant axes fitted to the data through a Monte Carlo resampling approach using the errors in *Q* and *U* measured at each wavelength bin.

After removal of the interstellar polarization (ISP) arising from the foreground interstellar dust (see the “Interstellar polarization” section), in Fig. 1, we present the temporal evolution of the intrinsic continuum polarization of SN 2024ggi at eight epochs from days 1.1 to 80.8. In each panel, different symbols mark the inverse 1σ error weighted mean polarization over the wavelength ranges identified in the color bar. In the top left and bottom right panels, the black dashed lines show the dominant axes of the first and last datasets. In these ISP-corrected data, *Q* = 0 and *U* = 0 are between the red and blue wavelengths at day 1.1 and near the blue end of the dominant axis at day 80.8. The data at intermediate epochs do not show clear dominant axes. These data are substantially displaced from *Q* = 0 and *U* = 0. A marked change of the continuum polarization (from days 1.1 to 2.0) is followed by a gradual drift until a roughly stationary geometry is reached at day 10.9, indicating a large-scale transformation of the geometry as the CSM is swept up by the SN ejecta. Throughout all analyses and figures, the ISP has been subtracted unless stated otherwise.

### Stage I—The shock-breakout phase

**Fig. 2.**
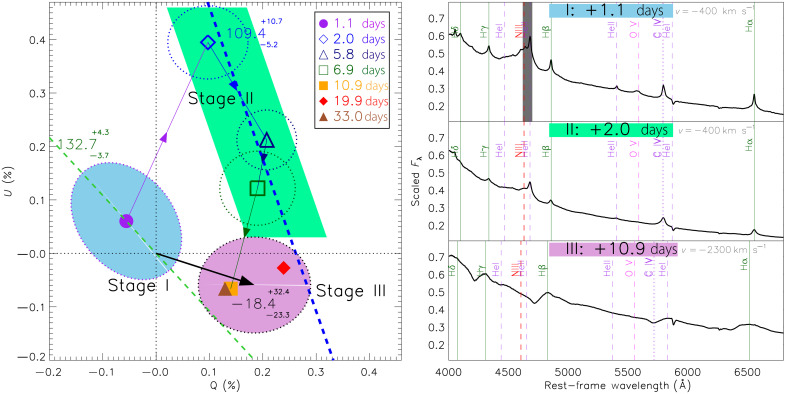
Temporal evolution of the continuum polarization of SN 2024ggi displayed in the *Q-U* plane. (**Left**) The blue-, green-, and pink-shaded areas mark the three stages of the SN 2024ggi polarimetry. Different symbols represent the continuum polarization of SN 2024ggi at different epochs. The thin green dashed line shows the dominant axis at day 1.1 for comparison. The blue dashed line approximately follows the stage II locus (days 2.0 to 6.9), when the interaction between the ejecta and CSM led to a change in overall geometry. The black arrow represents the PA of the continuum polarization of stage III, which was estimated by the error-weighted mean of days 10.9, 19.9, and 33.0. The size of each contour is determined by the standard deviation of the polarization measured at the encircled epoch(s). (**Right**) The top, middle, and bottom right panels show the scaled flux-density spectra ($F_{\lambda }$) at days 1.1 (stage I), 2.0 (stage II), and 10.9 (stage III), respectively, with major photoionized lines from several species labeled at velocity *v* in the rest frame. The region of the dark-gray–shaded band at day 1.1 suffers from detector saturation. Observations at day 80.8 are not presented as the polarization is affected by strong outward mixing of the inner He-rich layer and nickel clumps.

At very early epochs, the photosphere of SN 2024ggi was most likely engulfed in the CSM, as evidenced by several highly photoionized narrow features superposed on a blue continuum (see the “Polarization across the photoionized features” section) (*46*, *49*–*52*). The dynamical timescale is short on day 1, when the photospheric radius yields ≲1.5 × 10^14^ cm (*46*) and the ejecta expand rapidly. At day 1.1, the *Q-U* diagram shows a well-defined dominant axis with $2{\text{PA}}_{\text{day}1.1}=132^{\circ }.7_{-3^{\circ }.7}^{+4^{\circ }.3}$ (Fig. 1), where PA = 0.5 tan^−1^(*U*/*Q*). The distribution of the day 1.1 polarization can also be described by an ellipse, whose semimajor and semiminor axes are defined by the scatter about the dominant and orthogonal axes, namely, *a* ≈ 0.12% and *b* ≈ 0.09%, respectively (Fig. 2). As supported by the blueward *g-r* color evolution and the continuous rise of the until about day 1.6 (*46*, *49*, *52*), spectropolarimetry at day 1.1 measures the emission of the shock breakout, when photons promptly diffuse out of the optically thick CSM in certain directions. We note that such a geometry measurement is only feasible immediately after the onset of the shock breakout, during a brief moment when the shock has promptly emerged from the surface of the progenitor in some directions, while the remaining part of the shock is still embedded in the optically thick atmosphere or CSM. Therefore, the first epochs of spectropolarimetry of SN 2023ixf did not infer the shock breakout geometry (*47*, *53*–*55*), as the spread of the shock front to cover the entire surface of the SN 2023ixf progenitor persisted only for the first few hours (*32*).

We remark that the wavelength-dependent polarization on day 1.1 closely resembles the ISP as described by the empirical Serkowski law (*56*). Our attempts to characterize such a time-invariant redistribution of the data points on the *Q-U* plane imply that the day 1.1 polarization wavelength dependence is intrinsic to the SN (see the “Interstellar polarization” section). Instead, a wavelength-dependent photosphere would be expected for a spherically asymmetric shock breakout. The total observed intensity is a summation of various emitting components, each having an intensity of $I_{j}\left(\lambda \right)$ at a given wavelength λ. The net polarization is thus the total polarized flux normalized by the total flux, i.e.,

$$p\left(\lambda \right)=\sum_{j}I_{j}\left(\lambda \right)p_{j}\left(\lambda \right)/\;\sum_{j}I_{j}\left(\lambda \right)$$ (1)

Therefore, even if the polarization of each emission component with a characteristic blackbody temperature is wavelength independent, the net polarization can still be wavelength dependent.

Additional information on the geometry can be deduced from the polarization across spectral lines, which is especially sensitive to the geometric distribution of chemical species involved rather than the global shape represented by the photosphere and the continuum polarization. For a geometric structure with rotational symmetry, the *Q-U* diagram representing the wavelength bins within a spectral line reflects the geometry of the atomic species producing the line. The emitting regions at the earliest epoch most closely trace the ionization front of the shock propagating in the CSM, as indicated by the common dominant axis determined from the continuum and from the spectral features with the highest ionization potentials.

**Fig. 3.**
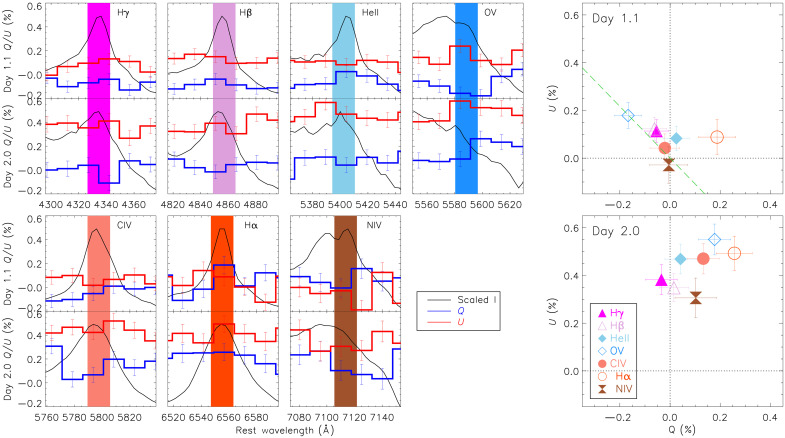
Polarizations measured in the central ±10 Å of various emission peaks at days 1.1 and 2.0. The emission cores as highlighted by the color-shaded spectral regions in the left subpanels are less affected by the electron-scattering emission from the wings (which are sensitive to smaller-scale structures such as lumpiness of the scattering region). Their distribution in the Stokes *Q-U* plane as shown in the top right and bottom right panels for days 1.1 and 2.0 (respectively), traces the geometry of the shock-breakout ionization front with the least influence from other effects. In the top right panel, the green dashed line presents the dominant axes determined over the wavelength range of 3800 to 7800 Å on day 1.1.

Because electron-scattering emission wings are sensitive to small-scale structure such as lumpiness in the scattering CSM, we focus on the emission cores of lines on day 1.1 of SN 2024ggi. The line cores are less affected by electron scattering than the line wings. The polarization of the line cores as displayed in the Stokes *Q-U* plane should more closely trace the geometry of the shock-breakout ionization front with the least influence from other effects. As illustrated in Fig. 3 (top right), all photoionized spectral features line up with the dominant axis on day 1.1. The only exception is Hα; the excitation energy of Hα (χ = 13.6 eV) is the lowest among all lines identified in the earliest flux spectrum and can thus be emitted over a wide range of angles with respect to the direction of the shock breakout so that any geometrical information is strongly diluted. By contrast, the highest excitation state, O V (χ = 113.9 eV), exhibits a clear dominant axis across the O V λ5597 feature similarly to the continuum (fig. S16).

This observational signature can be understood as the associated high ionization potential required by the highly ionized species to be realized close to the shock front, where the highest temperature produces the highest excitation states. The fact that the high-ionization lines (e.g., C IV λλ5801, 5812, N IV λλ7109, 7123, and O V λ5597) in the spectra of SN 2024ggi emerged after day 1.1, rather than before day 0.7 (*46*), is compatible with an early increase in photospheric temperature (*49*, *52*, *57*). This suggests a shock breakout within the CSM where a progressively hotter and stronger radiation field is emitted, in contrast to a shock-cooling process (*52*, *57*–*59*). Accordingly, the polarization of the continuum on day 1.1 traces the geometry of the emitting zone, where the shock breakout promptly leaks into the CSM. The line photons with the highest excitation potential on day 1.1 are formed close to the ionization front produced by the shock; thus, their polarization traces the pre-shocked CSM over the line-forming regions.

### Stage II—The ejecta-CSM interaction

From days 1.1 to 2.0, a clockwise rotation by 2PA ≈ 59° is seen among the data clouds in the Stokes *Q-U* plane, which represents the continuum polarization of more than 3800 to 7800 Å. The rotation continued at a slower rate after day 2.0 until the degree of continuum polarization settled at a roughly stationary level between days 10.9 and 33.0 (Fig. 1). Such temporal evolution does not necessarily imply a rotation of the symmetry axis in space, but it can be due to a change in the relative contributions of different structures to the total signal. Figure 2 summarizes the temporal evolution of the continuum polarization by resampling the observations at each epoch into very broad 800-Å wavelength bins. The green dashed line in Fig. 2 (left) shows the dominant axis as defined by the data on day 1.1. It represents the geometric axis of the photosphere at the earliest epoch, which is mostly within the CSM layer ionized by the shock-breakout flash. From days 2.0 to 6.9, the photosphere recedes into a deeper layer of the CSM where the emission produced by the expanding ejecta interacting with the CSM becomes progressively dominant. The time-evolving continuum polarization during stages I and II as displayed in Fig. 2 clearly reveals a misalignment between the shock breakout and the later ejecta-CSM interaction processes (see the “Modeling the polarization for an expanding envelope” and “The misaligned symmetry axes of the shock breakout and the ejecta-CSM interaction” sections).

The dominant axis can no longer be identified during stage II as seen in individual epochs compared to that on day 1.1 (Fig. 1). The temporal evolution of the continuum polarization measurements from days 2.0 to 6.9 follows a different path compared to the axial symmetry on day 1.1 (the blue dashed line in Fig. 2), demonstrating that the ejecta-CSM interaction process manifests a geometry different from that inferred during the shock-breakout phase: $2{\text{PA}}_{\text{CSM}}=109^{\circ }.8_{-5^{\circ }.2}^{+10^{\circ }.7}$ compared to $2{\text{PA}}_{\text{day}1.1}=132^{\circ }.7_{-3^{\circ }.7}^{+4^{\circ }.3}$, respectively. From days 2.0 to 6.9, lines from ions such as O V, N IV, C IV, and Hβ are much weaker, and their dominant axes become significantly less prominent.

**Fig. 4.**
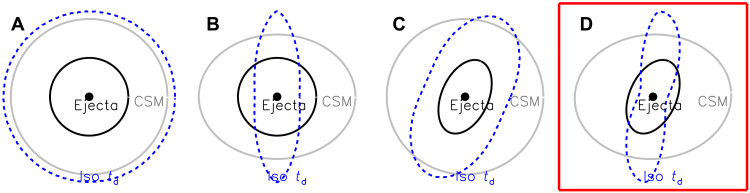
Illustration of the expanding ejecta and the invariant CSM for different explosion schematics. In each panel, the blue dashed contour displays the location of the ionization front estimated from the isodiffusion-time surface (see the “Schematic evolution of the geometry of the ionization front” section) and the solid gray circle/ellipse represents the outer boundary of the CSM, and the solid black circle/ellipse shows the outer boundary of the SN ejecta embedded in the CSM. The different schematics are (**A**) spherical ejecta and spherical CSM, (**B**) spherical ejecta and disk-concentrated CSM, (**C**) aspherical ejecta and spherical CSM, and (**D**) aspherical ejecta and disk-concentrated CSM. The axisymmetric prompt shock-breakout emission during stage I and the time-dependent symmetry axis during the transition to stage II suggest (D) as the most plausible scenario.

We sketch out four possible geometric configurations of the ejecta within the CSM in Fig. 4. The schematic drawing of the CSM exhibits a density variation that manifests as a moderate density enhancement toward a specific orientation, namely, the CSM plane (i.e., by a factor of ≲ 2; see the “Schematic evolution of the geometry of the ionization front” section). The schematics represent only the transition of the emission from stages I to II. On day 1.1, the photosphere displays an axially symmetric structure with a dominant axis that agrees with the shape of the shock breakout from the CSM, eliminating the doubly spherically symmetric case illustrated by Fig. 4A. The configuration evolves rapidly toward a geometry dominated by that of the CSM. However, we find that a spherical shock breakout sculpted by an aspherical CSM (Fig. 4B) and an aspherical shock breaking out of a spherical CSM (Fig. 4C) would both imprint a single symmetry axis at all times (see the “Polarization of the prolate and oblate geometric configurations” section; fig. S17). Both configurations would manifest as a progressive shrinkage of the distance between the data cloud and the zero point in the *Q-U* plane until the data sequence flips to the opposite direction (see the bottom row of fig. S17) instead of displaying the observed gradual rotation (Fig. 1) that draws a loop-like trajectory (Fig. 2). Therefore, we conclude that the symmetry axes of the shock breakout (day 1, green dashed line in Fig. 2) and the ejecta-CSM interaction (days 2 to 7, blue dashed line in Fig. 2) are misaligned, requiring an aspherical shock breakout from the progenitor surface as the explanation. We conclude that Fig. 4D, where the shock breakout and the CSM are both ellipsoidal but misaligned, is a more realistic representation of SN 2024ggi.

### Stage III—Dominance of the hydrogen-rich envelope

At later epochs (day 10.9 and thereafter), the characteristic P Cygni profiles of the Balmer lines are fully developed (fig. S20), implying that the receding photosphere has passed the inner boundary of the CSM and resides in the hydrogen-rich envelope of the exploding progenitor (see the “Polarization across the photoionized features” section for the temporal evolution of the spectral features). Polarimetry on and after day 10.9, thus, probes the geometry of the H-rich envelope of the outermost SN ejecta. The roughly circular, not elongated distribution of the data points in the Stokes *Q-U* plane hinders the identification of a dominant axis of SN 2024ggi at individual epochs. The PA of the H-rich envelope in stage III estimated from the error-weighted mean of the polarization on days 10.9, 19.9, and 33.0 yields $2{\text{PA}}_{\text{ej}}=-20^{\circ }.4_{-25^{\circ }.3}^{+32^{\circ }.4}$, which differs by ∼153° from the symmetry axis inferred for stage I. This change in PA close to a flip in the direction in the *Q-U* plane discloses a similar axial symmetry in stages I and III, with a geometric prolate-to-oblate transformation in between. As an example shown in the “The misaligned symmetry axes of the shock breakout and the ejecta-CSM Interaction” section and the top row of fig. S17, a small change of axial symmetry during stage II would manifest itself as a gradually rotating data cloud in the *Q-U* plane, which qualitatively accounts for the observed evolving continuum polarization of SN 2024ggi. In contrast, a flip of the dominant axis would imply a geometric transformation with the same symmetry axis [Fig. 2, the bottom row of fig. S17; see the “The misaligned symmetry axes of the shock breakout and the ejecta-CSM interaction” section; (*60*)].

**Fig. 5.**
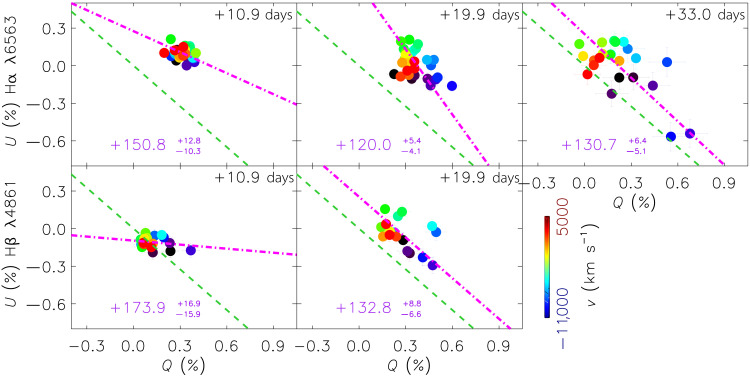
Time-variant polarization across the Balmer lines of SN 2024ggi. Evolution in the *Q-U* plane of Hα from days 10.9 to 33.0 and Hβ from days 10.9 to 19.9 are presented in the top and the bottom rows, respectively. The colors encode rest-frame velocities according to the color bars. In each panel, the magenta dot-dashed line fits the polarization distribution measured at different velocities that cover the corresponding spectral feature. The green dashed lines overplot the dominant axis at day 1.1, which appears to be aligned with that of the H envelope that has progressively emerged after day 6.9.

By approximating the electron-scattering atmosphere with an ellipsoid and a $\rho \left(r\right)\propto r^{-12}$ density distribution (*61*), the temporal evolution of the continuum polarization suggests moderate asphericity if viewed within ∼30° to 60° from the aspect angle of the observer, i.e., ∼0.8≲A≲0.95 and ∼1.2≲A≲1.4 for the prolate (before day 2.0, fig. S18) and the oblate (days 5.8 to 10.9, fig. S19) configurations, respectively (see the “Polarization of the prolate and oblate geometric configurations” section). From days 10.9 to 33.0 (stage III), the Hα and Hβ lines exhibit PAs of the dominant axes that are roughly consistent with the orientation of the data cloud and that of the shock breakout (Fig. 5. The only apparent exception is the Hβ line on day 33.0; however, it is caused by a blend with the emerging blueshifted Fe II λ5018 line (figs. S20 and S11). This tends to confirm that, except for stage II when the ejecta-CSM interaction is prominent, the axial symmetry derived from the continuum persists throughout the explosion of SN 2024ggi.

The detection of SN 2024ggi also in x-rays during the first few days (*62*–*64*) supports the notion that the early shock-breakout process is modified by a dense and confined CSM. The direct measurement of the shock-breakout geometry, which exhibits a spatially elongated, axially symmetric configuration (figs. S17 and S18), is also compatible with the blueward color evolution within the first day (*49*, *52*, *57*). The early polarization evolution of SN 2024ggi is highly complementary to the existence of the CSM and the way the CSM modifies the shock breakout. The symmetry axis defined by the shock breakout, which is aligned with that inferred for stage III, suggests that the core collapse could be driven by a mechanism that shapes the explosion on large scales. Moreover, the continuum polarization of SN 2024ggi shows a conspicuous time evolution but never exceeded ≲0.4% (*A* ≲ 1.4; see the “Polarization of the prolate and oblate geometric configurations” section), which is lower than the ≲2% and ~1% observed in the early phases of the type IIn SN 1998S (*65*) and type IIL/IIP SN 2023ixf (*47*, *53*–*55*). SN 1998S can be adequately modeled with a pole-to-equator density ratio of ~5 (*66*). In summary, the shock-breakout phase of SN 2024ggi shows a well-defined symmetry axis. The moderate global asymmetry is overall consistent with an asymmetry induced by an emitting zone extended in a particular direction.

## DISCUSSION

SN 2024ggi enables measurement of the shock-breakout geometry soon after the explosion. During this brief earliest moment, the geometry reflects the asymmetry of the explosion itself, as the photons toward the preferred directions of the explosion diffuse out promptly (Fig. 4D). SN 2024ggi is also the second of two H-rich CCSNe after SN 2023ixf (*32*, *67*) with spectrophotometric observations carried out days after shock breakout (*32*, *67*–*69*), for which significant asphericity during the shock breakout as well as ejecta engulfing CSM with large-scale asymmetry have been diagnosed (*47*, *53*). This may suggest a general pattern for the shock breakout from dying massive stars.

3D full-sphere SN simulations also suggest the development of large-scale asymmetries that manifest themselves as giant plumes of radioactive matter penetrating deeply into the helium and hydrogen envelopes (*31*, *70*). In contrast, the standing accretion shock instability (*71*, *72*) and a rather steep density gradient near the degenerate core will result in small-scale asymmetries in the ejecta (*73*). The shock breakout that evinces large-scale directional dependencies also indicates that the time at which the shock emerges on the progenitor surface along the plume-mixing or other directions could differ by ∼+0.7 days. Such a significantly aspherical explosion is also supported by very recent 3D hydrodynamic calculations, suggesting that the shock-breakout geometry could be shaped by neutrino-driven turbulence developed at the initial core collapse and preserved during the following few days (*31*). Although such a bubble-driven explosion is compatible with the observed large-scale asymmetry shared by the shock breakout and the SN ejecta, additional mechanisms that regulate the explosion to maintain a well-defined axial symmetry may still be needed. The early axisymmetric configuration of SN 2024ggi may also be compatible with a prompt outflow enhanced moderately toward the polar regions. Core collapses producing a neutron star and involving an amplified magnetic field through magnetorotational instability may lift matter along the rotational axis of the collapsing core (*74*, *75*). This process does not necessarily involve the formation of powerful jets that penetrate the helium and hydrogen envelopes, as implied by the moderate level of asphericity observed throughout the shock breakout and the ejecta expansion phases of SN 2024ggi. Details on how such a Lorentz-force-driven mechanism would account for the prompt axial symmetric emissions of SN 2024ggi require future quantitative model calculations.

Additional geometric clues include the spatially resolved axisymmetric structures consistent with a bipolar outflow in the Crab Nebula (*76*) and Cassiopeia A (*77*, *78*) that can be traced into the explosion zone. The explosion mechanism may be related to collapsar models for long-duration gamma-ray bursts (*79*) and even magnetar models of some superluminous SNe (*80*, *81*).

The misalignment of the axes of the CSM and the ejecta (Fig. 2) deserves further attention. The mass loss from the progenitor star may be governed by processes related to the angular momentum of the progenitor system, either as a single star or a binary companion, which may naturally produce the misalignment of the explosion and the CSM symmetry axes. Binary mass transfer during the common-envelope phase tends to enrich the CSM toward the orbital plane (*82*). Such a disk-concentrated CSM content, which is compatible with the polarization time series of SN 2024ggi, could be ubiquitous considering ≳80% of massive stars are in multiple systems (*83*). A magnetic field, which becomes more toroidal with distance from the progenitor, can also play a key role in shaping the CSM as inferred from well-structured planetary nebulae (*84*, *85*). Unlike the symmetry axis defined by the angular momentum of the system, the origins of the magnetic fields may be more complex and exhibit axes that are significantly different from the stellar rotation axis. For instance, the ejecta symmetry axis of SN 1987A is ∼28° away from the CSM symmetry axis (*86*), and the type IIn SN 1998S displayed conspicuously different PAs of the continuum polarization and the polarization across the Balmer lines (*25*, *65*). During the core collapse and the formation of the protoneutron star, the neutrino-driven instabilities or the initiation of jets through magnetorotational instabilities would also follow the structures of the progenitor stars (*87*–*89*), not the CSM. The combination of rotation encapsulated in the explosion geometry and magnetic fields encapsulated in the CSM geometry may naturally account for the misaligned axial symmetry between the ejecta and CSM.

Spectropolarimetry of SN 2024ggi reveals a moderately aspherical explosion that shows a well-defined symmetry axis shared by the prompt shock-breakout emission and the SN ejecta. This variability illustrates that instead of an amorphous/spherical setup resulting from small-scale instabilities, the core-collapse explosion of SN 2024ggi can be driven by a mechanism that shapes the explosion from the earliest shock breakout throughout the entire ejecta expansion.

## MATERIALS AND METHODS

### Interstellar polarization

To investigate the polarization intrinsic to SN 2024ggi, before proceeding to detailed discussions of the observed polarization, we derive the ISP induced by the dust grains along the SN-Earth line of sight. The ISP estimation was carried out using the earliest (day 1.1) and the day 80.8 polarization, where a global axially symmetric photosphere can be inferred from the presence of a prominent dominant axis.

After correction for the ISP, the dominant axis is seen as a straight line passing through the origin, as expected for an axisymmetric configuration (*90*, *91*), and the intersection of the dominant axes on days 1.1 and 80.8 at $Q_{\text{ISP}}=-0.40\pm 0.05$% and $U_{\text{ISP}}=0.54\pm 0.04$% yields the ISP (fig. S1). The ISP is weakly dependent on wavelength, in particular within the low-ISP regime (*90*). In the coordinate system defined by the Stokes *Q* and *U* parameters [i.e., the *Q-U* plane; (*25*)], the effect of the ISP is largely the introduction of uncertainties of the zero point. It is also expected to be time-independent (*24*) and manifests as an offset in the Stokes *Q-U* diagram without affecting the morphological patterns of the data points. High-resolution spectroscopic observations of the Na I D absorption doublet have led to the conclusion that the line-of-sight extinction toward SN 2024ggi can be decomposed into a Galactic [$E{\left(B-V\right)}_{\text{MW}}=0.120\pm 0.028$ magnitude (mag)] and a host-galaxy [$E{\left(B-V\right)}_{\text{host}}=0.034$ mag; (*52*)] component. Because interstellar extinction and polarization are both induced by dust grains (*56*), the stronger Galactic extinction suggests that the Galactic polarization is the dominant component of the ISP. In the case of SN 2024ggi, the exact ISP level is difficult to estimate with the widely used standard methods [e.g., (*92*, *93*)]. In particular, the absence of resolved cores of emission profiles dominated by unpolarized photons released by recombination is a handicap.

Polarization by dust grains in the interstellar matter shifts the dominant axis away from the origin in the *Q-U* plane. For SN ejecta with a high degree of axial symmetry, the ISP would be located at one of the ends (or beyond them) of the dominant axis (*2*, *40*, *94*). If the variability of an object causes the dominant axis of the intrinsic polarization to rotate, then the rotation angle is independent of the chosen value of the ISP because the latter only introduces a displacement of the data points from the origin. However, careful subtraction of the ISP is of paramount importance when determining the shape of an object from its intrinsic polarization.

Another approach to estimate the total line-of-sight ISP assumes that the emission peak of the strong P Cygni profiles of the Balmer lines is unpolarized during the photospheric phase of type II/IIP SNe (*91*). We estimate the error-weighted mean polarization within a wavelength range of 6550 to 6750 Å to be $Q_{\text{ISP}}^{+33d}=-0.32\pm 0.04\%$ and $U_{\text{ISP}}^{+33d}=0.55\pm 0.08\%$, consistent with the estimate presented above.

We also estimate the ISP from the spectropolarimetry of SN 2024ggi at day 267.1. Because the ejecta expand and the electron-scattering cross section decreases as $\propto t^{2}$, the SN has entered the nebular phase at such a late epoch. Except for several polarized blueshifted absorption components of the P Cygni profile (see the “Spectropolarimetry of SN 2024ggi” section), the continuum spectrum during the nebular phase can be treated as an unpolarized source dominated by significantly blended emission lines from various iron-group elements, which are free from electron scattering and intrinsically unpolarized. Therefore, the continuum polarization on day 267.1 also measures the ISP toward the SN. We measure the error-weighted mean continuum polarization of more than 4000 to 6300 Å as $Q_{\text{ISP}}^{+267d}=-0.25\pm 0.24\%$ and $U_{\text{ISP}}^{+267d}=0.62\pm 0.24\%$, consistent with the other methods. Throughout this paper, $Q_{\text{ISP}}=-\;0.41\%\pm 0.05\%$ and $U_{\text{ISP}}=0.55\%\pm 0.04\%$ are adopted for the intrinsic polarization of SN 2024ggi. These approaches provide different values compared to the Galactic ISP sampled by a bright star ∼1° away from SN 2024ggi. The result of this sanity check is discussed in the Supplementary Text and fig. S2.

The wavelength-dependent polarization of SN 2024ggi at day 1.1 shows a remarkable resemblance to the characteristic wavelength-dependent Serkowski law. In the low ISP regime, the observed wavelength dependence can be well fitted by a single ISP component, consistent with the single-cloud interpretation based on a comprehensive investigation on the effects of ISP induced by various interstellar dust contents (*95*). However, high-resolution spectroscopy of SN 2024ggi obtained at ≈3 days after its explosion reveals at least three major discrete absorbing components (*52*). Therefore, the ISP toward SN 2024ggi may not follow a single cloud model that accounts for the day 1.1 observation.

We also conducted a sanity test to verify that the wavelength dependence of the polarization across the observed wavelength range on day 1.1 is intrinsic to the SN. The wavelength (λ) dependence of the ISP can be approximated by the empirical Serkowski law (*56*)

$$p\left(\lambda \right)/p\left(\lambda_{\text{max}}\right)=\text{exp}\left[-K\;{\text{ln}}^{2}\left(\lambda_{\text{max}}/\lambda \right)\right]$$ (2)

where $\lambda_{\text{max}}$ and $p\left(\lambda_{\text{max}}\right)$ represent the wavelength and the level of the maximum polarization, respectively. The parameter *K* characterizes the width of the peak of the ISP. By attributing the wavelength-dependent polarization of SN 2024ggi on day 1.1 to the ISP, we fitted a Serkowski law to the polarization spectra and present the results in fig. S3 (left). However, as illustrated in fig. S4, the removal of the wavelength dependence derived based on the day 1.1 observation would introduce significant wavelength-dependent polarization at all other epochs. As neither the endpoints nor the line segment passes through the origin on the *Q-U* plane from days 5.8 to 80.8, we conclude that the ISP cannot be naturally accounted for by the wavelength-dependent polarization on day 1.1. The latter, which persisted only briefly after the SN explosion, is therefore intrinsic to the SN and traces the geometry of the shock breakout.

In fig. S3 (right), we overlay the best-fit Serkowski law to the day 1.1 observation onto the polarization of SN 2024ggi on day 267.1, when the SN has entered the nebula phase. We investigated the wavelength dependence of the day 267.1 polarization by resampling the data with large (150 Å) wavelength bins. The result does not reproduce Serkowski’s fit to the day 1.1 observations, further strengthening the claim that the day 1.1 polarization is intrinsic to the SN, rather than the ISP.

### Spectropolarimetry of SN 2024ggi

Spectropolarimetry of SN 2024ggi was carried out with the FOcal Reducer and low-dispersion Spectrograph 2 [FORS2; (*96*)] on Unit Telescope 1 (UT1, Antu) of the Very Large Telescope at the ESO’s Paranal site in Chile. Each observation in the Polarimetric Multi-Object Spectroscopy (PMOS) mode consists of eight exposures at retarder-plate angles of 0°, 22.5°, 45°, and 67.5°. All observations were carried out using the 300V grism and a 1″-wide slit. Therefore, the resolving power and the intrinsic width of each resolution element near its central wavelength at 5849 Å are *R* ∼ 440 and ∼13.3 Å, respectively, corresponding to ∼680 km s^−1^ (*97*). The observing log is available in Table 1.

Preprocessing of the 2D images obtained at each retarder plate angle and the extraction of the ordinary (o) and extraordinary (e) beams were carried out with standard procedures within Image Reduction and Analysis Facility (IRAF) (*98*, *99*). Wavelength calibration of each individual spectrum was performed separately, with a typical root mean square accuracy of ∼0.20 Å. Following the prescriptions in (*100*), we then derived the Stokes parameters and calculated the observed polarization degree ($p_{\text{obs}}$) and PA (${\text{PA}}_{\text{obs}}$)

$$p_{\text{obs}}=\sqrt{Q^{2}+U^{2}},\;{\text{PA}}_{\text{obs}}=\frac{1}{2}\text{arctan}\left(\frac{U}{Q}\right)$$ (3)

where *Q* and *U* denote the intensity (*I*)–normalized Stokes parameters. An additional debiasing procedure was applied because the true value of the polarization degree is nonnegative (*101*)

$$\begin{array}{l} p=\left(p_{\text{obs}}-\frac{\sigma_{p}^{2}}{p_{\text{obs}}}\right)\;\times \;h\left(p_{\text{obs}}-\sigma_{p}\right)\\ \text{PA}={\text{PA}}_{\text{obs}} \end{array}$$ (4)

where $\sigma_{p}$ and *h* denote the 1σ uncertainty in $p_{\text{obs}}$ and the Heaviside step function, respectively. Following the prescription described in previous work [e.g., (*93*, *102*)], where the wavelength-dependent instrumental polarization in the PMOS mode of FORS2 (≲0.1%) was characterized to remain stable over time, this effect was corrected according to the characterization by (*103*). The low instrumental polarization during the campaign of SN 2024ggi polarimetry is consistent with that inferred from the observations of polarized and unpolarized standard stars carried out in each night with observations for our program.

Throughout the paper, all spectra and spectropolarimetry data of SN 2024ggi were corrected to the rest frame adopting the heliocentric recession velocity of NGC 3621 of 730 km s^−1^ [*z* ≈ 0.002435; (*104*)]. Spectropolarimetry of SN 2024ggi obtained from days 1.1 to 267.1 is displayed in figs. S5 to S13. All data are presented in the rest frame and before correcting for the ISP. Principal component decomposition of the SN 2024ggi spectropolarimetry is shown in fig. S14 to better visualize the temporal evolution of the total-flux spectra and the polarization spectra projected onto the dominant axis and the orthogonal axis.

### Polarization across the photoionized features

The exceptionally early-epoch polarimetry includes the short-lived photoionization-powered emission lines during the first days of SN 2024ggi. In the first ∼2 days, the total-flux emission profiles consist of a prominent emission peak and a weak, broad underlying component with full width at half maximum intensity of ≈1000 to 2000 km s^–1^ (fig. S15). We also computed the polarized flux density $p\times f_{\lambda }$ across the flash features and found no significant deviation from the adjacent continuum. The broad wings are due to scattering by free electrons in the unshocked, photoionized CSM (*105*–*107*). The polarization of the electron-scattering wings traces the spatial distribution of the associated ionic species. The spectral-line-specific geometric diagnostics are best derived in the Stokes *Q-U* plane by comparing, epoch by epoch, the location of a given spectral line and that of the continuum (*25*). The slope of the distribution of the data points represents the orientation of the symmetry axis of the feature in question (line or continuum), projected onto the plane of the sky.

High-ionization lines (e.g., O V λ5597) appear only at the earliest phases and are generally thought to form in the relatively inner part of the CSM and close to the shock front, where the highest temperatures produce the highest ionization states. In the case of a spherically symmetric shock breakout and the resulting concentric ionization front, their identical shapes would manifest as a single dominant axis in the continuum and for all early-time emission lines. In SN 2024ggi (fig. S16), the polarization PAs of the spectral lines with the highest ionization potentials (e.g., O V, χ = 113.9 eV) follow that of the underlying continuum, while other lines such as C IV λ5807 (=64.5 eV) and Hα (=13.6 eV) exhibit distinctly different dominant axes than the continuum. Due to a saturation issue within the rest-frame wavelength range of 4630 to 4710 Å that covers the He II λ4686 (χ = 54.5 eV) emission line at day 1.1, this region was excluded from the analysis of the line polarization.

Although both Hα and Hβ arise from the recombination to the second excitation level of hydrogen, the transition probability expressed as the form of weighted oscillator strength [log(*gf*)] of Hα is a factor of ∼5 higher than that of the Hβ. Therefore, higher polarization can be expected for Hα wherever an energy level of 13.6 eV is reached. Compared to Hα, Hβ would mainly form at a much narrower region. The polarization is also weaker and only becomes dominant close to the photosphere, thus effectively tracing the geometry of the ionization front as early as day 1.1. The agreement between the dominant axes of the continuum and the distribution of the high-excitation lines on the *Q*-*U* diagram as presented in Fig. 3 further strengthens the interpretation of the axially symmetric configuration of the shock breakout. Portraits of the early-phase photoionized spectral features are offered in fig. S16. The O V line itself, whose electron-scattering wings are likely to arise from the CSM confined to the most energetic shock-ionization front, exhibits a relatively clear dominant axis that is similar to that of the continuum.

The line polarization behavior is also compatible with the picture inferred from the continuum polarization. As the shock-ionized emission preferentially emerges promptly from the less dense regions perpendicular to the plane of the CSM disc, the shock would propagate faster toward the perpendicular directions when the ejecta have not yet overrun the CSM. Consequentially, the faster shock heats the postshock gas to a higher temperature, thus producing the earliest prolate geometry that is aligned with the less-dense regions perpendicular to the CSM plane. In contrast, the denser CSM plane will decelerate the shock more strongly, resulting in a lower postshock temperature. The lower-ionization lines would preferentially be developed in this lower-temperature region and occupy a broad range in CSM-plane azimuth angle.

### Modeling the polarization for an expanding envelope

Following the general assumptions of the Sobolev approximation [e.g., (*108*)], we treat the SN atmosphere with a low-velocity gradient in its inner region, below some velocity cutoff $v_{\text{cut}}$ of a few thousand kilometers per second, that radiates as a blackbody and is surrounded by an expanding atmosphere with a significantly larger velocity power-law exponent *n*. The density of the atmosphere at a given time (*t*) after the explosion and different radial velocities ($v_{r}$) below and above the layer with $v_{\text{cut}}$ are given by

$$\rho_{\text{in}}\left(t\right)\propto {\left(\frac{t}{t_{0}}\right)}^{-2},\;\rho_{\text{out}}\left(v_{r},t\right)\propto {\left(\frac{v_{\text{cut}}}{v_{r}}\right)}^{n}\times {\left(\frac{t}{t_{0}}\right)}^{-2}$$ (5)

respectively. We denote as *u* and *v* the two components of $v_{r}$ that are projected onto and perpendicular to the plane of the sky, respectively. Therefore

$$\theta ={\text{tan}}^{-1}\left(|\frac{u}{v}|\right),v_{r}=\sqrt{u^{2}+v^{2}},\mu =\sqrt{1-{\left(\frac{u}{v_{\text{ph}}}\right)}^{2}}$$ (6)

where $v_{\text{ph}}$ represents the rest-frame velocity of the photosphere at a given *t*. For an atmosphere of free electrons governed by Thomson scattering, the intensities of the electric vectors parallel ($I_{l}$) and perpendicular ($I_{r}$) to the plane of the sky were adopted from equations 122 and 123 of (*109*). With this, the polarization degree *p* and the intensity-normalized Stokes *Q* and *U* parameters can be derived as

$$\begin{array}{c} I_{0}\left(\mu \right)=I_{r}\left(\mu \right)+I_{l}\left(\mu \right),\;p=\frac{I_{r}\left(\mu \right)-I_{l}\left(\mu \right)}{I_{r}\left(\mu \right)+I_{l}\left(\mu \right)},\;Q=p\text{cos}\left(2\phi \right),\;U=p\text{sin}\left(2\phi \right) \end{array}$$ (7)

Here, ϕ is the longitude measured toward the line of sight.

Following (*108*, *110*), we calculated the shape of the P Cygni profile of the Hα line under the Sobolev approximation. The flux density profile $f_{\text{env}}$ was computed separately for the blue side ($v<-\;v_{\text{ph}}$), the middle region ($-\;v_{\text{ph}}\leq v<0$ km s^−1^), and the red side (v≥0 km s^−1^). This prescription assumes a spherical atmosphere established soon after the SN explosion. To account for the effect of asphericity, we introduce a geometric factor A(θ,ϕ). By multiplying by the optical depth calculated for specific line velocities in the rest frame, this function characterizes the directional dependence of the emission.

To investigate the overall geometric properties of the line-emitting region, we adopted for the sake of simplicity an oblate spatial distribution of the optical depth, namely

$$\frac{x^{2}+y^{2}}{a^{2}}+\frac{z^{2}}{c^{2}}=1\;$$ (8)

$$A\left(\theta ,\phi \right)={\left[\frac{\text{cos}{\left(\theta \right)}^{2}\text{sin}{\left(\phi \right)}^{2}}{a^{2}}+\frac{\text{sin}{\left(\theta \right)}^{2}\text{sin}{\left(\phi \right)}^{2}}{a^{2}}+\frac{\text{cos}{\left(\phi \right)}^{2}}{c^{2}}\right]}^{-\frac{1}{2}}$$ (9)

Therefore

$$I=f_{\text{env}}A\left(\theta ,\phi \right)\;$$ (10)

The polarization is then calculated as

q=Ipcos2ϕsin2θ (11)

u=Ipsin2ϕsin2θ (12)

### The misaligned symmetry axes of the shock breakout and the ejecta-CSM interaction

With the most plausible scenario suggested by the temporal evolution of the polarization of SN 2024ggi (Figs. 1 and 4; see the “Interstellar polarization” section), we expect a ${\mathrm{180}}^{\circ }$ difference between the PA estimated at stages I and III because the transition from a prolate to oblate geometry must go through a point with zero polarization and flip the signs of the Stokes *Q-U* parameters. A basic flip in the orientation of the *Q* and *U* polarization distribution is illustrated in the bottom row of fig. S17 for the case when the prolate and oblate components have a common symmetry axis. For this model, the polarization of the electron-scattering emitting region is calculated for an expanding envelope (see the “Modeling the polarization for an expanding envelope” section), which can be linearly decomposed into a “prolate” and an “oblate” component. The former and the latter represent the prompt and the later emission that mainly originate from the directions perpendicular to and within the CSM plane, respectively.

On day 2.0, the continuum polarization jumps to its peak, i.e., from $\begin{array}{c} {\left[Q,U\right]}^{\text{day}1.1}=\left[-0.043\;\pm \;0.074\%,\;0.046\;\pm \;0.077\%\right] \end{array}$ to ${\left[Q,U\right]}^{\text{day}2.0}=\left[+0.110\;\pm \;0.075\%,\;0.381\;\pm \;0.069\%\right]$ (Fig. 1), computed as the error-weighted mean values of more than 3800 to 7800 Å. The continuum polarization subsequently decreases monotonically. We hereby break down the possible configurations of the ejecta and the CSM displayed in Fig. 4. In Fig. 4A, both the ejecta and the CSM are spherical, so that there will be no net polarization. In Fig. 4B, the shock emerges from the star spherically symmetrically, and the asphericity is entirely due to the CSM. In Fig. 4C, prolate ejecta will lead to a prompt diffusion of photons from a spherical CSM along certain directions. Configurations illustrated by Fig. 4 (B and C) exhibit only one symmetry axis, producing a single dominant axis in the *Q-U* diagram (*2*). The breakout emission would thus emerge promptly toward the direction where a shorter diffusion time is achieved (see the “Schematic evolution of the geometry of the ionization front” section), producing a prolate photosphere as represented by the equal-arrival-time contour. As a consequence, the dominant axis would shrink monotonically and its orientation remains constant over time until a flip of the signs of *Q* and *U* takes place (see the bottom row of fig. S17). The blue and green dashed lines in Fig. 2 would also coincide. The fact that we observed two distinctly different axes in Fig. 1 disfavors the schematic scenario presented in Fig. 4B. A similar argument applies to the alternative where the ejecta are aspherical and the CSM is spherically symmetric (Fig. 4C).

If an aspherical shock breaks through the surface of the progenitor into a nonspherical CSM (Fig. 4D), then the behavior is more complex. The early polarization would also tend to be that of a prolate structure but aligned with the axes of neither the ejecta nor the CSM. There would be a complex interaction between the ejecta and the CSM, and the polarization tends to show an oblate geometry as the photosphere recedes toward the H-rich envelope of the SN ejecta. At later times when the CSM becomes transparent, the polarization should probe the geometry of deeper layers of the ejecta. This qualitative behavior is reflected in our observations. The gradually rotating distribution of the polarization of SN 2024ggi in the Stokes *Q-U* plane from days 1.1 to 6.9, which can only be disclosed by the time-resolved data, suggests a misalignment between the aforementioned symmetry axes (see the top row of fig. S17). Consequently, the continuum polarization paints a “loop-like” trajectory over time (Fig. 2).

Models illustrating the gradual rotation of the direction of the centers of the data cloud on the Stokes *Q-U* plane over time, assuming one prolate (blue) and one oblate (red) emission component each, are presented in fig. S17. The top and the bottom rows display the two symmetry axes misaligned by $∼\;{\mathrm{20}}^{\circ }$ and aligned scenarios, respectively. The presented models adopt $v_{\text{cut}}=1000$ km s^−1^, a density index of *n* = 1.5, and a viewing angle of $\theta_{0}={\mathrm{90}}^{\circ }$ and $\phi_{0}={\mathrm{70}}^{\circ }$. Coefficients that are arbitrarily assigned to describe the relative strengths of the prolate and the oblate components (i.e., [$c_{p}$, $c_{o}$]) for the four epochs are [0.5, 0.0], [0.4, 1.2], [0.2, 1.6], and [0.1, 2.0]. We note that the aim of fig. S17 is to provide a schematic illustration of the polarization time evolution for the cases of a time-variant and a fixed axisymmetry, as presented in the top and the bottom rows, respectively. The continuum polarization of SN 2024ggi draws a loop-like trajectory (Fig. 2), suggesting that the ejecta-CSM interaction exhibits a different geometry compared to that measured at the shock breakout and the H-rich ejecta.

### Schematic evolution of the geometry of the ionization front

As a simple representation of the aspherical CSM profile that would produce the proposed prolate-to-oblate geometric transformation, outside the expanding early ejecta, we adopt a spherical CSM envelope that exhibits density variation throughout the azimuth, where the highest density is achieved near the denoted CSM plane. We assume that the CSM is centered on the SN and quickly swept up by the matter ejected by the SN explosion. The time between the emission of a photon from the surface of the SN ejecta and its diffusion out of the surrounding CSM can be estimated for any point at the outer CSM boundary as

$$t_{d}=\frac{\kappa \rho \left(r,\theta \right)\Delta R^{2}}{c}\;$$ (13)

where =0.34 cm^2^ g^−1^ gives the opacity, ρ(r,θ) represents the number density of the medium at distance *r* and viewing angle θ, ΔR denotes the diffusion length from the ejecta to the location (r,θ) in the CSM, and *c* is the speed of light.

The density profile of the CSM can be described as

$$\rho \left(r,\theta \right)=\rho_{0}r^{-n}\left[|\text{cos}\left(\theta \right)|+\;\rho_{\text{min}}\right]\;$$ (14)

where $\rho_{\text{min}}$ indicates the minimum density of the CSM at a latitude angle of ϕ and *n* denotes the power-law index of the radial density distribution. We estimate a characteristic isodiffusion-time contour by adding up the distances between a given point of the CSM and all points on the ejecta surface and dividing the lengths of each path by the associated photon travel speed. The former counts only the line segments that connect the given point on the outer boundary of the CSM to the ejecta surface, while the latter is dependent on the number density of the CSM where the photon is traveling through (Fig. 4). The schematic isodelay contour takes into account the asphericity of the ejecta as well as a disk-concentrated configuration of the CSM. The isodiffusion-time surface can then be sketched over the entire CSM surface. The isodiffusion-time surface can then be sketched over the entire CSM surface.

When the shock propagates outward and progressively runs into the CSM, the shock breakout can be seen toward the less-dense regions as hinted by the dominant axes measured across the continuum, which aligned with the photoionized features (Fig. 4). For a spherical CSM embedding a spherical shock breakout, photons will emerge from the CSM isotropically; thus, no polarization would be expected as a consequence of the persistent spherical symmetry (Fig. 4A). Any deviation from spherical symmetry in the CSM or the ejecta would produce an aspherical isophoton-travel-time surface. Examples for the former case with a less-dense CSM toward the perpendicular directions and the latter case with a stretched ejecta are given in Fig. 4 (B and C), respectively. When both configurations are aspherical and misaligned by a certain angle, the prolate geometry is manifested as an interplay between the internal shock breakout and the external CSM density distribution (Fig. 4D). On day 1, lower-excitation lines can be found over a wide range in azimuth. The emitting region traced by integrating the reciprocal of the diffusion time calculated over the SN ejecta exhibits a peanut shape (Fig. 4D). For this configuration, the symmetry axis connects the perpendicular directions, which have the lowest CSM density.

### Polarization of the prolate and oblate geometric configurations

We use the 3D Monte Carlo polarization simulation code (*111*) for electron-scattering–dominated photospheres to estimate the deviation from spherical symmetry of SN 2024ggi at various phases. Following the prescription provided by (*112*), this technique has been implemented in many SN polarization calculations (*41*, *113*–*115*). We discretize the space by a 100by100by100 3D grid with uniform density (ρ) and electron-scattering opacity ($\kappa_{\text{es}}$). Unpolarized Monte Carlo photon packets are emitted from an electron-scattering–dominated photosphere with an even surface brightness, where the Stokes parameters of each photon packet are initialized as

$$I=\left(\begin{array}{c} I\\ Q\\ U \end{array}\right)=\left(\begin{array}{c} 1\\ 0\\ 0 \end{array}\right)$$ (15)

For different ellipsoidal envelope configurations, Eq. 8 can be rewritten in cylindrical coordinates as

$$\frac{r^{2}}{A^{2}}+z^{2}=c^{2}$$ (16)

where we introduce the axis ratio (A=a/c), with A<1 and A>1 representing the prolate and the oblate configurations, respectively. The ellipsoidal envelope along radial isodensity surfaces can be expressed as

$$\rho \left(\xi \right)=\rho_{0}\xi^{-n}$$ (17)

where

$$\xi =\sqrt{\frac{r^{2}}{A^{2}}+z^{2}}\;$$ (18)

In all calculations, we adopt a power-law index n=12 (*61*) considering the rather dense and steep density profile of the outer layers of the ejecta within the first few days after the SN explosion. The maximum photosphere radius ($R_{\text{ph}}$) is determined by the position where the electron-scattering optical depth (τ) along the semimajor axis of the ellipsoidal envelope equals unity, where

$$\tau =\int_{R_{\text{ph}}}^{\xi_{\text{out}}}\kappa_{\text{es}}\rho \left(\xi \right)\mathrm{dz}=1\;$$ (19)

Here, $\xi_{\text{out}}$ denotes the outer boundary of the ellipsoidal envelope. Each photon packet is assigned a random optical depth τ=−ln(z) (0<z≤1) during its propagation, indicating that scattering will occur whenever an electron packet reaches this optical depth while traversing the medium. Each scattering would alter the Stokes parameters of the photon packet through multiplying the rotation [L(ϕ)] and the phase [R(Θ)] matrices

$$I_{\text{out}}=L\left(\pi -i_{2}\right)R\left(\Theta \right)L\left(-i_{1}\right)I_{\text{in}}\;$$ (20)

where $I_{\text{in}}$ and $I_{\text{out}}$ denote the set of Stokes parameters in the rest frame before and after a certain scattering event, respectively. Terms $i_{1}$ and $i_{2}$ denote the angles on the spherical triangle as defined in (*116*). The rotation matrix yields

$$L\left(\phi \right)=\left(\begin{array}{ccc} 1 & 0 & 0\\ 0 & \text{cos}2\phi & \text{sin}2\phi \\ 0 & -\;\text{sin}2\phi & \text{cos}2\phi \end{array}\right)$$ (21)

and the phase matrix in the scattering frame can be written as

$$\begin{array}{c} R\left(\Theta \right)=\frac{3}{4}\left(\begin{array}{ccc} {\text{cos}}^{2}\Theta \;+\;1 & {\text{cos}}^{2}\Theta \;-\;1 & 0\\ {\text{cos}}^{2}\Theta \;-\;1 & {\text{cos}}^{2}\Theta \;+\;1 & 0\\ 0 & 0 & 2\text{cos}\Theta \end{array}\right) \end{array}$$ (22)

where Θ is the scattering angle in the scattering plane, which has been chosen by sampling its probability distribution function ($f_{\text{pdf}}$)

$$\begin{array}{c} \begin{array}{c} f_{\text{pdf}}\left(\Theta ,i_{1}\right)=\frac{1}{2}\left({\text{cos}}^{2}\Theta \;+\;1\right)\\ +\frac{1}{2}\left({\text{cos}}^{2}\Theta -1\right)\left(\text{cos}2i_{1}Q_{\text{in}}/I_{\text{in}}-\text{sin}2i_{1}U_{\text{in}}/I_{\text{in}}\right) \end{array} \end{array}$$ (23)

Therefore, $i_{1}$ and cosΘ can be sampled from a uniform distribution

$$\begin{array}{l} i_{1}=2{\pi \xi }_{1}\\ \;\text{cos}\Theta =1-2\xi_{2} \end{array}$$ (24)

The random seeds $\xi_{1}$ and $\xi_{2}$ are chosen from a uniform distribution on the interval [0, 1]. After each scattering, the photon packet will travel along a different direction determined by $i_{1}$ and cosΘ. This process continues until the photon packet escapes the computational boundary and will be collected in different viewing angle (θ) bins depending on its final direction $\vec{n}$. The continuum polarization of prolate and oblate photospheres seen from different viewing angles θ is presented in figs. S18 and S19, respectively.

We remark that the purpose of these calculations is to provide a rough quantitative justification of the inferred bipolar shock breakout and the subsequent prolate-to-oblate geometric transformation. The latter is also naturally reproduced by the calculations for a prolate (days 1.1 and 2.0, fig. S18) and an oblate (days 5.8 to 10.9, fig. S19) configuration. A schematic of the temporal evolution of the emission component as approximated by the combination of these two is also illustrated in fig. S17. Within the optically thick regime (τ>1), the peak of the polarization degree decreases as the optical depth increases until it reaches its asymptotic value at τ≳4 [figure 1 of (*113*)]. Therefore, the estimated ellipticities yield a lower bound of the departure of the photosphere from spherical symmetry.

### Polarization across the Balmer and He II lines

The systematically blueshifted Hα profile with emission peak velocities of ~–3000 to −2000 km s^−1^ from days 10.9 to 33.0 [fig. S20, see also (*51*, *52*)] suggests a rather steep radial density structure of the H-rich envelope of SN 2024ggi. This can be understood as an enhanced occultation of the receding side of the ejecta, namely, extincted by gas on the approaching side, leading to a suppressed emission toward the red end of the emission profile (*117*). Furthermore, a rather steep density gradient during the early recombination phase is expected (*61*). Under such a high-density regime, the Balmer emission is driven by a combination of electron scattering and collisional bound-bound excitation. The line polarization profile may thus be formed due to a combined effect of the aspherical limb of the photosphere and the line excitation (*118*). The latter may lead to an uneven blocking of the underlying photosphere that induces polarization. The universal symmetry axis shared by the prolate shock breakout and the oblate H-rich envelope further strengthens the proposal of an aspherical explosion of SN 2024ggi, with a well-defined symmetry axis.

Additionally, our monitoring of the polarization of SN 2024ggi until day 80.8 shows several distinct temporal trends (fig. S21). Portraits of the spectral evolution of the Hα and Hβ lines are provided in fig. S16. First, a dominant axis with $2{\text{PA}}_{+80.8d}=37^{\circ }.0_{-5^{\circ }.1}^{+5^{\circ }.9}$ can be identified (Fig. 1). A similar PA is measured after excluding the He I and Balmer lines, $2\text{PA}=34^{\circ }.5_{-5^{\circ }.2}^{+4^{\circ }.6}$ (fig. S1). Second, the He I λ5876 line has emerged, the polarization of which tightly follows a well-defined dominant axis of 2 PA$2{\text{PA}}^{\text{HeI}}=+19^{\circ }.0_{-4^{\circ }.7}^{+4^{\circ }.9}$ that is $∼{\mathrm{33}}^{\circ }$ off from the symmetry axis shared by the earliest prolate and the later oblate configurations (Fig. 5). A rather small misalignment between the well-defined dominant axis of the ejecta and that of He I λ5876 is inferred, $2{\text{PA}}_{+80.8d}-2{\text{PA}}^{\text{HeI}}\approx {\mathrm{16}}^{\circ }$. The presence of both strong Balmer and He II lines and their different dominant axes, therefore, suggests that the mixing of helium into the still optically thick H-rich envelope exhibits a different symmetry axis.

Determining the He-rich layer geometry requires spectropolarimetry after the photosphere recedes through the H-rich envelope, the inner ejecta exhibit a symmetry axis as indicated by the dominant axis fitted to the continuum at day 80.8, which is misaligned with the outermost H-rich envelope as defined by a prolate-to-oblate geometry transformation since the shock breakout. A more complicated inner geometry can be inferred from the deviations from axial symmetry in moderate scales as indicated by the departure from the dominant axis at day 80.8 (Fig. 1, bottom right; and fig. S2, right). This is also compatible with the main features of the neutrino-driven explosion that manifests on a large scale: bubbles and fractured structures (*119*). Fallback-induced accretion may be involved in reshaping the inner geometry of the ejecta (*12*, *120*).

## Supplementary Materials

### This PDF file includes:

Supplementary Text Figs. S1 to S21 References

## References

[1] T. D. Lee, S. Drell, Particle physics and introduction to field theory. Phys. Today 34, 55–56 (1981).

[2] L. Wang, J. C. Wheeler, Spectropolarimetry of supernovae. Ann. Rev. Astron. Astrophys. 46, 433–474 (2008).

[3] S. J. Smartt, J. J. Eldridge, R. M. Crockett, J. R. Maund, The death of massive stars - I. Observational constraints on the progenitors of Type II-P supernovae. Mon. Not. R. Astron. Soc. 395, 1409–1437 (2009).

[4] S. J. Smartt, Observational constraints on the progenitors of core-collapse supernovae: The case for missing high-mass stars. Pub. Astron. Soc. Aust. 32, e016 (2015).

[5] W. Li, J. Leaman, R. Chornock, A. V. Filippenko, D. Poznanski, M. Ganeshalingam, X. Wang, M. Modjaz, S. Jha, R. J. Foley, N. Smith, Nearby supernova rates from the Lick Observatory Supernova Search - II. The observed luminosity functions and fractions of supernovae in a complete sample. Mon. Not. R. Astron. Soc. 412, 1441–1472 (2011).

[6] A. Burrows, Colloquium: Perspectives on core-collapse supernova theory. Rev. Mod. Phys. 85, 245 (2013).

[7] A. Burrows, T. Wang, D. Vartanyan, Physical correlations and predictions emerging from modern core-collapse supernova theory. Astrophys. J. Lett. 964, L16 (2024).

[8] T. Wang, A. Burrows, Supernova explosions of the lowest-mass massive star progenitors. Astrophys. J. 969, 74 (2024).

[9] C. D. Ott, A. Burrows, T. A. Thompson, E. Livne, R. Walder, The spin periods and rotational profiles of neutron stars at birth. Astrophys. J. Suppl. Ser. 164, 130–155 (2006).

[10] A. Burrows, L. Dessart, E. Livne, C. D. Ott, J. Murphy, Simulations of magnetically driven supernova and hypernova explosions in the context of rapid rotation. Astrophys. J. 664, 416–434 (2007).

[11] J. M. LeBlanc, J. R. Wilson, A numerical example of the collapse of a rotating magnetized star. Astrophys. J. 161, 541 (1970).

[12] A. M. Khokhlov, P. A. Höflich, E. S. Oran, J. C. Wheeler, L. Wang, A. Y. Chtchelkanova, Jet-induced explosions of core collapse supernovae. Astrophys. J. 524, L107–L110 (1999).

[13] K. Maeda, K. Nomoto, Bipolar supernova explosions: Nucleosynthesis and implications for abundances in extremely metal-poor stars. Astrophys. J. 598, 1163–1200 (2003).

[14] M. Obergaulinger, H.-T. Janka, M. A. Aloy, Magnetic field amplification and magnetically supported explosions of collapsing, non-rotating stellar cores. Mon. Not. R. Astron. Soc. 445, 3169–3199 (2014).

[15] P. Mösta, C. D. Ott, D. Radice, L. F. Roberts, E. Schnetter, R. Haas, A large-scale dynamo and magnetoturbulence in rapidly rotating core-collapse supernovae. Nature 528, 376–379 (2015). 26618868 10.1038/nature15755

[16] H.-T. Janka, T. Melson, A. Summa, Physics of core-collapse supernovae in three dimensions: A sneak preview. Ann. Rev. Nucl. Part. Sci. 66, 341–375 (2016).

[17] B. Aschenbach, R. Egger, J. Trümper, Discovery of explosion fragments outside the Vela supernova remnant shock-wave boundary. Nature 373, 587–590 (1995).

[18] N. Soker, The role of jets in exploding supernovae and in shaping their remnants. Res. Astron. Astrophys. 22, DOI:10.1088/1674-4527/ac9782 (2022).

[19] A. G. Lyne, D. R. Lorimer, High birth velocities of radio pulsars. Nature 369, 127–129 (1994).

[20] B. M. S. Hansen, E. S. Phinney, The pulsar kick velocity distribution. Mon. Not. R. Astron. Soc. 291, 569–577 (1997).

[21] H.-T. Janka, Explosion mechanisms of core-collapse supernovae. Ann. Rev. Nucl. Part. Sci. 62, 407–451 (2012).

[22] H.-T. Janka, Neutron star kicks by the gravitational tug-boat mechanism in asymmetric supernova explosions: Progenitor and explosion dependence. Astrophys. J. 837, 84 (2017).

[23] T. M. Tauris, M. Kramer, P. C. C. Freire, N. Wex, H.-T. Janka, N. Langer, P. Podsiadlowski, E. Bozzo, S. Chaty, M. U. Kruckow, E. P. J. van den Heuvel, J. Antoniadis, R. P. Breton, D. J. Champion, Formation of double neutron star systems. Astrophys. J. 846, 170 (2017).

[24] P. Hoeflich, J. C. Wheeler, D. C. Hines, S. R. Trammell, Analysis of the polarization and flux spectra of SN 1993J. Astrophys. J. 459, 307–321 (1996).

[25] L. Wang, D. A. Howell, P. Höflich, J. C. Wheeler, Bipolar supernova explosions. Astrophys. J. 550, 1030–1035 (2001).

[26] J. R. Maund, J. C. Wheeler, D. Baade, F. Patat, P. Höflich, L. Wang, A. Clocchiatti, The early asymmetries of supernova 2008D/XRF 080109. Astrophys. J. 705, 1139–1151 (2009).

[27] A. Perego, M. Hempel, C. Fröhlich, K. Ebinger, M. Eichler, J. Casanova, M. Liebendörfer, F.-K. Thielemann, PUSHing core-collapse supernovae to explosions in spherical symmetry I: The model and the case of SN 1987A. Astrophys. J. 806, 275 (2015).

[28] E. O’Connor, R. Bollig, A. Burrows, S. Couch, T. Fischer, H.-T. Janka, K. Kotake, E. J. Lentz, M. Liebendörfer, O. E. B. Messer, A. Mezzacappa, T. Takiwaki, D. Vartanyan, Global comparison of core-collapse supernova simulations in spherical symmetry. J. Phys. G Nucl. Part. Phys. 45, 104001 (2018).

[29] S. M. Couch, M. L. Warren, E. P. O’Connor, Simulating turbulence-aided neutrino-driven core-collapse supernova explosions in one dimension. Astrophys. J. 890, 127 (2020).

[30] B. Müller, D. W. Gay, A. Heger, T. M. Tauris, S. A. Sim, Multidimensional simulations of ultrastripped supernovae to shock breakout. Mon. Not. R. Astron. Soc. 479, 3675–3689 (2018).

[31] D. Vartanyan, B.-T. Tsang, D. Kasen, A. Burrows, T. Wang, L. Teryoshin, A 3D simulation of a Type II-P supernova: From core bounce to beyond shock breakout. Astrophys. J. 982, 9 (2025).

[32] G. Li, M. Hu, W. Li, Y. Yang, X. Wang, S. Yan, L. Hu, J. Zhang, Y. Mao, H. Riise, X. Gao, T. Sun, J. Liu, D. Xiong, L. Wang, J. Mo, A. Iskandar, G. Xi, D. Xiang, L. Wang, G. Sun, K. Zhang, J. Chen, W. Lin, F. Guo, Q. Liu, G. Cai, W. Zhou, J. Zhao, J. Chen, X. Zheng, K. Li, M. Zhang, S. Xu, X. Lyu, A. J. Castro-Tirado, V. Chufarin, N. Potapov, I. Ionov, S. Korotkiy, S. Nazarov, K. Sokolovsky, N. Hamann, E. Herman, A shock flash breaking out of a dusty red supergiant. Nature 627, 754–758 (2024). 38093004 10.1038/s41586-023-06843-6

[33] A. Gal-Yam, I. Arcavi, E. O. Ofek, S. Ben-Ami, S. B. Cenko, M. M. Kasliwal, Y. Cao, O. Yaron, D. Tal, J. M. Silverman, A. Horesh, A. De Cia, F. Taddia, J. Sollerman, D. Perley, P. M. Vreeswijk, S. R. Kulkarni, P. E. Nugent, A. V. Filippenko, J. C. Wheeler, A WolfRayet-like progenitor of SN 2013cu from spectral observations of a stellar wind. Nature 509, 471–474 (2014). 24848059 10.1038/nature13304

[34] D. Khazov, O. Yaron, A. Gal-Yam, I. Manulis, A. Rubin, S. R. Kulkarni, I. Arcavi, M. M. Kasliwal, E. O. Ofek, Y. Cao, D. Perley, J. Sollerman, A. Horesh, M. Sullivan, A. V. Filippenko, P. E. Nugent, D. A. Howell, S. B. Cenko, J. M. Silverman, H. Ebeling, F. Taddia, J. Johansson, R. R. Laher, J. Surace, U. D. Rebbapragada, P. R. Wozniak, T. Matheson, Flash spectroscopy: Emission lines from the ionized circumstellar material around <10-day-old Type II supernovae. Astrophys. J. 818, 1329868 (2016).

[35] O. Yaron, D. A. Perley, A. Gal-Yam, J. H. Groh, A. Horesh, E. O. Ofek, S. R. Kulkarni, J. Sollerman, C. Fransson, A. Rubin, P. Szabo, N. Sapir, F. Taddia, S. B. Cenko, S. Valenti, I. Arcavi, D. A. Howell, M. M. Kasliwal, P. M. Vreeswijk, D. Khazov, O. D. Fox, Y. Cao, O. Gnat, P. L. Kelly, P. E. Nugent, A. V. Filippenko, R. R. Laher, P. R. Wozniak, W. H. Lee, U. D. Rebbapragada, K. Maguire, M. Sullivan, M. T. Soumagnac, Confined dense circumstellar material surrounding a regular type II supernova. Nat. Phys. 13, 510–517 (2017).

[36] L. Dessart, D. J. Hillier, E. Audit, Explosion of red-supergiant stars: Influence of the atmospheric structure on shock breakout and early-time supernova radiation. Astron. Astrophys. 605, A83 (2017).

[37] R. J. Bruch, A. Gal-Yam, S. Schulze, O. Yaron, Y. Yang, M. Soumagnac, M. Rigault, N. L. Strotjohann, E. Ofek, J. Sollerman, F. J. Masci, C. Barbarino, A. Y. Q. Ho, C. Fremling, D. Perley, J. Nordin, S. B. Cenko, S. Adams, I. Adreoni, E. C. Bellm, N. Blagorodnova, M. Bulla, K. Burdge, K. De, S. Dhawan, A. J. Drake, D. A. Duev, A. Dugas, M. Graham, M. L. Graham, I. Irani, J. Jencson, E. Karamehmetoglu, M. Kasliwal, Y.-L. Kim, S. Kulkarni, T. Kupfer, J. Liang, A. Mahabal, A. A. Miller, T. A. Prince, R. Riddle, Y. Sharma, R. Smith, F. Taddia, K. Taggart, R. Walters, L. Yan, A large fraction of hydrogen-rich supernova progenitors experience elevated mass loss shortly prior to explosion. Astrophys. J. 912, 46 (2021).

[38] R. J. Bruch, A. Gal-Yam, O. Yaron, P. Chen, N. L. Strotjohann, I. Irani, E. Zimmerman, S. Schulze, Y. Yang, Y.-L. Kim, M. Bulla, J. Sollerman, M. Rigault, E. Ofek, M. Soumagnac, F. J. Masci, C. Fremling, D. Perley, J. Nordin, S. B. Cenko, A. Y. Q. Ho, S. Adams, I. Adreoni, E. C. Bellm, N. Blagorodnova, K. Burdge, K. De, R. G. Dekany, S. Dhawan, A. J. Drake, D. A. Duev, M. Graham, M. L. Graham, J. Jencson, E. Karamehmetoglu, M. M. Kasliwal, S. Kulkarni, A. A. Miller, J. D. Neill, T. A. Prince, R. Riddle, B. Rusholme, Y. Sharma, R. Smith, N. Sravan, K. Taggart, R. Walters, L. Yan, The prevalence and influence of circumstellar material around hydrogen-rich supernova progenitors. Astron. Astrophys. 952, 119 (2023).

[39] W. V. Jacobson-Galán, L. Dessart, K. W. Davis, C. D. Kilpatrick, R. Margutti, R. J. Foley, R. Chornock, G. Terreran, D. Hiramatsu, M. Newsome, E. P. Gonzalez, C. Pellegrino, D. A. Howell, A. V. Filippenko, J. P. Anderson, C. R. Angus, K. Auchettl, K. A. Bostroem, T. G. Brink, R. Cartier, D. A. Coulter, T. de Boer, M. R. Drout, N. Earl, K. Ertini, J. R. Farah, D. Farias, C. Gall, H. Gao, M. A. Gerlach, F. Guo, A. Haynie, G. Hosseinzadeh, A. L. Ibik, S. W. Jha, D. O. Jones, D. Langeroodi, N. LeBaron, E. A. Magnier, A. L. Piro, S. I. Raimundo, A. Rest, S. Rest, R. M. Rich, C. Rojas-Bravo, H. Sears, K. Taggart, V. A. Villar, R. J. Wainscoat, X.-F. Wang, A. R. Wasserman, S. Yan, Y. Yang, J. Zhang, W. Zheng, Final moments. II. Observational properties and physical modeling of circumstellar-material-interacting Type II supernovae. Astrophys. J. 970, 189 (2024).

[40] L. Wang, D. Baade, P. Höflich, A. Khokhlov, J. C. Wheeler, D. Kasen, P. E. Nugent, S. Perlmutter, C. Fransson, P. Lundqvist, Spectropolarimetry of SN 2001el in NGC 1448: Asphericity of a normal Type Ia supernova. Astrophys. J. 591, 1110–1128 (2003).

[41] D. Kasen, P. Nugent, L. Wang, D. A. Howell, J. C. Wheeler, P. Höflich, D. Baade, E. Baron, P. H. Hauschildt, Analysis of the flux and polarization spectra of the Type Ia supernova SN 2001el: Exploring the geometry of the high-velocity ejecta. Astrophys. J. 593, 788–808 (2003).

[42] S. Srivastav, T. W. Chen, S. J. Smartt, M. Nicholl, K. W. Smith, D. R. Young, M. Fulton, M. McCollum, T. Moore, J. Weston, X. Sheng, A. Aamer, C. R. Angus, P. Ramsden, L. Shingles, J. Gillanders, L. Rhodes, A. Andersson, H. Stevance, L. Denneau, J. Tonry, H. Weiland, A. Lawrence, R. Siverd, N. Erasmus, W. Koorts, A. Jordan, V. Suc, A. Rest, C. Stubbs, J. Sommer, ATLAS24fsk (AT2024ggi): Discovery of a nearby candidate SN in NGC 3621 at 7 Mpc with a possible progenitor detection. Trans. Name Server AstroNote 100, 1 (2024).

[43] A. Saha, F. Thim, G. A. Tammann, B. Reindl, A. Sandage, Cepheid distances to SNe Ia host galaxies based on a revised photometric zero point of the HST WFPC2 and new PL relations and metallicity corrections. Astrophys. J. Suppl. Ser. 165, 108–137 (2006).

[44] Q. Zhai, L. Li, Z. Wang, J. Zhang, X. Wang, LJT spectroscopic classification of AT 2024ggi as a young Type II supernova with flash features. Trans. Name Server AstroNote 104, 1 (2024).

[45] J. L. Tonry, L. Denneau, A. N. Heinze, B. Stalder, K. W. Smith, S. J. Smartt, C. W. Stubbs, H. J. Weiland, A. Rest, ATLAS: A high-cadence all-sky survey system. Publ. Astron. Soc. Pac. 130, 064505 (2018).

[46] J. Zhang, L. Dessart, X. Wang, Q. Zhai, Y. Yang, L. Li, H. Lin, G. Valerin, Y. Cai, Z. Guo, L. Wang, Z. Zhao, Z. Wang, S. Yan, Probing the shock breakout signal of SN 2024ggi from the transformation of early flash spectroscopy. Astrophys. J. Lett. 970, L18 (2024).

[47] S. S. Vasylyev, Y. Yang, A. V. Filippenko, K. C. Patra, T. G. Brink, L. Wang, R. Chornock, R. Margutti, E. L. Gates, A. J. Burgasser, P. R. Karpoor, N. LeBaron, E. Softich, C. A. Theissen, E. Wiston, W. Zheng, Early time spectropolarimetry of the aspherical Type II supernova SN 2023ixf. Astrophys. J. Lett. 955, L37 (2023).

[48] J. R. Maund, J. C. Wheeler, L. Wang, D. Baade, A. Clocchiatti, F. Patat, P. Höflich, J. Quinn, P. Zelaya, A spectropolarimetric view on the nature of the peculiar Type I SN 2005hk. Astrophys. J. Lett. 722, 1162–1174 (2010).

[49] W. V. Jacobson-Galán, K. W. Davis, C. D. Kilpatrick, L. Dessart, R. Margutti, R. Chornock, R. J. Foley, P. Arunachalam, K. Auchettl, C. R. Bom, R. Cartier, D. A. Coulter, G. Dimitriadis, D. Dickinson, M. R. Drout, A. T. Gagliano, C. Gall, B. Garretson, L. Izzo, D. O. Jones, N. LeBaron, H.-Y. Miao, D. Milisavljevic, Y.-C. Pan, A. Rest, C. Rojas-Bravo, A. Santos, H. Sears, B. M. Subrayan, K. Taggart, S. Tinyanont, SN 2024ggi in NGC 3621: Rising ionization in a nearby, circumstellar-material-interacting Type II supernova. Astrophys. J. 972, 177 (2024).

[50] D. Xiang, J. Mo, X. Wang, L. Wang, J. Zhang, H. Lin, L. Chen, C. Song, L.-D. Liu, Z. Wang, G. Li, The red supergiant progenitor of Type II supernova 2024ggi. Astrophys. J. Lett. 969, L15 (2024).

[51] T. Pessi, R. Cartier, E. Hueichapan, D. de Brito Silva, J. L. Prieto, R. R. Muñoz, G. E. Medina, P. Diaz, T. S. Li, Early emission lines in SN 2024ggi revealed by high-resolution spectroscopy. Astron. Astrophys. 688, L28 (2024).

[52] M. Shrestha, K. A. Bostroem, D. J. Sand, G. Hosseinzadeh, J. E. Andrews, Y. Dong, E. Hoang, D. Janzen, J. Pearson, J. E. Jencson, M. J. Lundquist, D. Mehta, A. P. Ravi, N. Meza Retamal, S. Valenti, P. J. Brown, S. W. Jha, C. Macrie, B. Hsu, J. Farah, D. A. Howell, C. McCully, M. Newsome, E. Padilla Gonzalez, C. Pellegrino, G. Terreran, L. Kwok, N. Smith, M. Schwab, A. Martas, R. R. Munoz, G. E. Medina, T. S. Li, P. Diaz, D. Hiramatsu, B. E. Tucker, J. C. Wheeler, X. Wang, Q. Zhai, J. Zhang, A. Gangopadhyay, Y. Yang, C. P. Gutiérrez, Extended shock breakout and early circumstellar interaction in SN 2024ggi. Astrophys. J. Lett. 972, L15 (2024).

[53] A. Singh, R. S. Teja, T. J. Moriya, K. Maeda, K. S. Kawabata, M. Tanaka, R. Imazawa, T. Nakaoka, A. Gangopadhyay, M. Yamanaka, V. Swain, D. K. Sahu, G. C. Anupama, B. Kumar, R. M. Anche, Y. Sano, A. Raj, V. K. Agnihotri, V. Bhalerao, D. Bisht, M. S. Bisht, K. Belwal, S. K. Chakrabarti, M. Fujii, T. Nagayama, K. Matsumoto, T. Hamada, M. Kawabata, A. Kumar, R. Kumar, B. K. Malkan, P. Smith, Y. Sakagami, K. Taguchi, N. Tominaga, A. Watanabe, Unravelling the asphericities in the explosion and multifaceted circumstellar matter of SN 2023ixf. Astrophys. J. 975, 132 (2024).

[54] M. Shrestha, S. DeSoto, D. J. Sand, G. G. Williams, J. L. Hoffman, P. S. Smith, C. McCall, J. R. Maund, I. A. Steele, K. Wiersema, J. E. Andrews, N. Smith, C. Bilinski, P. Milne, R. M. Anche, K. A. Bostroem, G. Hosseinzadeh, J. Pearson, D. C. Leonard, B. Hsu, Y. Dong, E. Hoang, D. Janzen, J. E. Jencson, S. W. Jha, M. J. Lundquist, D. Mehta, N. M. Retamal, S. Valenti, J. Farah, D. A. Howell, C. McCully, M. Newsome, E. P. Gonzalez, C. Pellegrino, G. Terreran, Spectropolarimetry of SN 2023ixf reveals both circumstellar material and an aspherical helium core. Astrophys. J. Lett. 982, L32 (2025).

[55] S. S. Vasylyev, L. Dessart, Y. Yang, A. V. Filippenko, K. C. Patra, T. G. Brink, L. Wang, R. Chornock, R. Margutti, E. L. Gates, A. J. Burgasser, H. Sears, P. R. Karpoor, N. LeBaron, E. Softich, C. A. Theissen, E. Wiston, W. Zheng, Spectropolarimetrice of SN 2023ixf: An asymmetric explosion in a confined aspherical circumstellar medium. arXiv:2505.03975. (2025).

[56] K. Serkowski, D. S. Mathewson, V. L. Ford, Wavelength dependence of interstellar polarization and ratio of total to selective extinction. Astrophys. J. 196, 261–290 (1975).

[57] T.-W. Chen, S. Yang, S. Srivastav, T. J. Moriya, S. J. Smartt, S. Rest, A. Rest, H. W. Lin, H.-Y. Miao, Y.-C. Cheng, A. Aryan, C.-Y. Cheng, M. Fraser, L.-C. Huang, M.-H. Lee, C.-H. Lai, Y.-H. Liu, A. Sankar.K, K. W. Smith, H. F. Stevance, Z.-N. Wang, J. P. Anderson, C. R. Angus, T. de Boer, K. Chambers, H.-Y. Duan, N. Erasmus, M. Fulton, H. Gao, J. Herman, W.-J. Hou, H.-Y. Hsiao, M. E. Huber, C.-C. Lin, H.-C. Lin, E. A. Magnier, K. K. Man, T. Moore, C.-C. Ngeow, M. Nicholl, P.-S. Ou, G. Pignata, Y.-C. Shiau, J. S. Sommer, J. L. Tonry, X.-F. Wang, R. Wainscoat, D. R. Young, Y.-T. Yeh, J. Zhang, J. Zhang, Discovery and extensive follow-up of SN 2024ggi, a nearby Type IIP supernova in NGC 3621. Astrophys. J. 983, 86 (2025).

[58] E. Waxman, B. Katz, in *Handbook of Supernovae*, A. W. Alsabti, P. Murdin, Eds. (Springer, 2017), p. 967.

[59] J. Morag, N. Sapir, E. Waxman, Shock cooling emission from explosions of red supergiants - I. A numerically calibrated analytic model. Mon. Not. R. Astron. Soc. 522, 2764–2776 (2023).

[60] M. Tanaka, K. Maeda, P. A. Mazzali, K. S. Kawabata, K. Nomoto, Three-dimensional explosion geometry of stripped-envelope core-collapse supernovae. II. Modeling of polarization. Astrophys. J. 837, 105 (2017).

[61] A. Chieffi, I. Domínguez, P. Höflich, M. Limongi, O. Straniero, Theoretical light curves of Type II-P supernovae and applications to cosmology. Mon. Not. R. Astron. Soc. 345, 111–122 (2003).

[62] A. A. Lutovinov, A. N. Semena, I. A. Mereminskiy, S. Y. Sazonov, S. V. Molkov, A. Y. Tkachenko, V. A. Arefiev, SRG/ART-XC detects SN2024ggi in X-rays. Astron. Telegr. 16586, 1 (2024).

[63] R. Margutti, B. Grefenstette, NuSTAR detection of SN2024ggi at 2 days post discovery. Astron. Telegr. 16587, 1 (2024).

[64] J. Zhang, C. K. Li, H. Q. Cheng, Q. Y. Wu, S. M. Jia, Y. Chen, W. W. Cui, H. Feng, J. Guan, D. W. Han, W. Li, C. Z. Liu, F. J. Lu, L. M. Song, J. Wang, J. J. Xu, S. N. Zhang, H. S. Zhao, X. F. Zhao, C. C. Jin, Z. X. Ling, H. Y. Liu, M. J. Liu, Y. Liu, D. Y. Li, H. Sun, W. Yuan, C. Zhang, W. D. Zhang, R. Z. Li, Y. Wang, H. Zhou, K. Nandra, A. Rau, P. Friedrich, N. Meidinger, V. Burwitz, E. Kuulkers, A. Santovincenzo, P. O’Brien, B. Cordier, X. F. Wang, W. X. Li, SN 2024ggi: Detection of X-ray emission by EP-FXT. Astron. Telegr. 16588, 1 (2024).

[65] D. C. Leonard, A. V. Filippenko, A. J. Barth, T. Matheson, Evidence for asphericity in the Type IIN supernova SN 1998S. Astrophys. J. 536, 239–254 (2000).

[66] L. Dessart, D. C. Leonard, S. S. Vasylyev, D. J. Hillier, Spectropolarimetric modeling of interacting Type II supernovae. Application to early-time observations of SN 1998S. Astron. Astrophys. 696, L12 (2025).

[67] E. A. Zimmerman, I. Irani, P. Chen, A. Gal-Yam, S. Schulze, D. A. Perley, J. Sollerman, A. V. Filippenko, T. Shenar, O. Yaron, S. Shahaf, R. J. Bruch, E. O. Ofek, A. De Cia, T. G. Brink, Y. Yang, S. S. Vasylyev, S. Ben Ami, M. Aubert, A. Badash, J. S. Bloom, P. J. Brown, K. De, G. Dimitriadis, C. Fransson, C. Fremling, K. Hinds, A. Horesh, J. P. Johansson, M. M. Kasliwal, S. R. Kulkarni, D. Kushnir, C. Martin, M. Matuzewski, R. C. McGurk, A. A. Miller, J. Morag, J. D. Neil, P. E. Nugent, R. S. Post, N. Z. Prusinski, Y. Qin, A. Raichoor, R. Riddle, M. Rowe, B. Rusholme, I. Sfaradi, K. M. Sjoberg, M. Soumagnac, R. D. Stein, N. L. Strotjohann, J. H. Terwel, T. Wasserman, J. Wise, A. Wold, L. Yan, K. Zhang, The complex circumstellar environment of supernova 2023ixf. Nature 627, 759–762 (2024). 38538936 10.1038/s41586-024-07116-6

[68] G. Hosseinzadeh, J. Farah, M. Shrestha, D. J. Sand, Y. Dong, P. J. Brown, K. A. Bostroem, S. Valenti, S. W. Jha, J. E. Andrews, I. Arcavi, J. Haislip, D. Hiramatsu, E. Hoang, D. A. Howell, D. Janzen, J. E. Jencson, V. Kouprianov, M. Lundquist, C. McCully, N. E. M. Retamal, M. Modjaz, M. Newsome, E. P. Gonzalez, J. Pearson, C. Pellegrino, A. P. Ravi, D. E. Reichart, N. Smith, G. Terreran, J. Vinkó, Shock cooling and possible precursor emission in the early light curve of the Type II SN 2023ixf. ApJ 953, L16 (2023).

[69] W. V. Jacobson-Galán, L. Dessart, R. Margutti, R. Chornock, R. J. Foley, C. D. Kilpatrick, D. O. Jones, K. Taggart, C. R. Angus, S. Bhattacharjee, L. A. Braff, D. Brethauer, A. J. Burgasser, F. Cao, C. M. Carlile, K. C. Chambers, D. A. Coulter, E. Dominguez-Ruiz, C. B. Dickinson, T. de Boer, A. Gagliano, C. Gall, H. Gao, E. L. Gates, S. Gomez, M. Guolo, M. R. J. Halford, J. Hjorth, M. E. Huber, M. N. Johnson, P. R. Karpoor, T. Laskar, N. LeBaron, Z. Li, Y. Lin, S. D. Loch, P. D. Lynam, E. A. Magnier, P. Maloney, D. J. Matthews, M. McDonald, H.-Y. Miao, D. Milisavljevic, Y.-C. Pan, S. Pradyumna, C. L. Ransome, J. M. Rees, A. Rest, C. Rojas-Bravo, N. R. Sandford, L. S. Ascencio, S. Sanjaripour, A. Savino, H. Sears, N. Sharei, S. J. Smartt, E. R. Softich, C. A. Theissen, S. Tinyanont, H. Tohfa, V. A. Villar, Q. Wang, R. J. Wainscoat, A. L. Westerling, E. Wiston, M. A. Wozniak, S. K. Yadavalli, Y. Zenati, SN 2023ixf in Messier 101: Photo-ionization of dense, close-in circumstellar material in a nearby Type II supernova. Astrophys. J. 954, L42 (2023).

[70] D. Vartanyan, M. S. B. Coleman, A. Burrows, The collapse and three-dimensional explosion of three-dimensional massive-star supernova progenitor models. Mon. Not. R. Astron. Soc. 510, 4689–4705 (2022).

[71] J. M. Blondin, A. Mezzacappa, C. DeMarino, Stability of standing accretion shocks, with an eye toward core-collapse supernovae. Astrophys. J. 584, 971–980 (2003).

[72] F. Hanke, B. Müller, A. Wongwathanarat, A. Marek, H.-T. Janka, SASI activity in three-dimensional neutrino-hydrodynamics simulations of supernova cores. Astrophys. J. 770, 66 (2013).

[73] G. Stockinger, H.-T. Janka, D. Kresse, T. Melson, T. Ertl, M. Gabler, A. Gessner, A. Wongwathanarat, A. Tolstov, S.-C. Leung, K. Nomoto, A. Heger, Three-dimensional models of core-collapse supernovae from low-mass progenitors with implications for Crab. Mon. Not. R. Astron. Soc. 496, 2039–2084 (2020).

[74] C. Winteler, R. Käppeli, A. Perego, A. Arcones, N. Vasset, N. Nishimura, M. Liebendörfer, F.-K. Thielemann, Magnetorotationally driven supernovae as the origin of early galaxy *r*-process elements? Astrophys. J. Lett. 750, L22 (2012).

[75] P. Mösta, S. Richers, C. D. Ott, R. Haas, A. L. Piro, K. Boydstun, E. Abdikamalov, C. Reisswig, E. Schnetter, Magnetorotational core-collapse supernovae in three dimensions. Astrophys. J. 785, L29 (2014).

[76] M. C. Weisskopf, J. J. Hester, A. F. Tennant, R. F. Elsner, N. S. Schulz, H. L. Marshall, M. Karovska, J. S. Nichols, D. A. Swartz, J. J. Kolodziejczak, S. L. O’Dell, Discovery of spatial and spectral structure in the X-ray emission from the Crab Nebula. Astrophys. J. 536, L81–L84 (2000). 10859123 10.1086/312733

[77] J. M. Laming, U. Hwang, B. Radics, G. Lekli, E. Takács, The polar regions of Cassiopeia A: The aftermath of a gamma-ray burst? Astrophys. J. 644, 260–273 (2006).

[78] D. Milisavljevic, T. Temim, I. De Looze, D. Dickinson, J. M. Laming, R. Fesen, J. C. Raymond, R. G. Arendt, J. Vink, B. Posselt, G. G. Pavlov, O. D. Fox, E. Pinarski, B. Subrayan, J. Schmidt, W. P. Blair, A. Rest, D. Patnaude, B.-C. Koo, J. Rho, S. Orlando, H.-T. Janka, M. Andrews, M. J. Barlow, A. Burrows, R. Chevalier, G. Clayton, C. Fransson, C. Fryer, H. L. Gomez, F. Kirchschlager, J.-J. Lee, M. Matsuura, M. Niculescu-Duvaz, J. D. R. Pierel, P. P. Plucinsky, F. D. Priestley, A. P. Ravi, N. S. Sartorio, F. Schmidt, M. Shahbandeh, P. Slane, N. Smith, N. Sravan, K. Weil, R. Wesson, J. C. Wheeler, A JWST survey of the supernova remnant Cassiopeia A. Astrophys. J. Lett. 965, L27 (2024).

[79] A. I. MacFadyen, S. E. Woosley, Collapsars: Gamma-ray bursts and explosions in “failed supernovae”. Astrophys. J. 524, 262–289 (1999).

[80] D. Kasen, L. Bildsten, Supernova light curves powered by young magnetars. Astrophys. J. 717, 245–249 (2010).

[81] S. E. Woosley, Bright supernovae from magnetar birth. Astrophys. J. 719, L204–L207 (2010).

[82] A. Ercolino, H. Jin, N. Langer, L. Dessart, Interacting supernovae from wide massive binary systems. Astron. Astrophys. 685, A58 (2024).

[83] S. Li, P. Sanhueza, H. Beuther, H.-R. V. Chen, R. Kuiper, F. A. Olguin, R. E. Pudritz, I. W. Stephens, Q. Zhang, F. Nakamura, X. Lu, R. L. Kuruwita, T. Sakai, T. Henning, K. Taniguchi, F. Li, Observations of high-order multiplicity in a high-mass stellar protocluster. Nat. Astron. 8, 472–481 (2024).

[84] R. A. Chevalier, D. Luo, Magnetic shaping of planetary nebulae and other stellar wind bubbles. Astrophys. J. 421, 225 (1994).

[85] E. G. Blackman, A. Frank, J. A. Markiel, J. H. Thomas, H. M. Van Horn, Dynamos in asymptotic-giant-branch stars as the origin of magnetic fields shaping planetary nebulae. Nature 409, 485–487 (2001). 11206538 10.1038/35054008

[86] L. Wang, J. C. Wheeler, P. Höflich, A. Khokhlov, D. Baade, D. Branch, P. Challis, A. V. Filippenko, C. Fransson, P. Garnavich, R. P. Kirshner, P. Lundqvist, R. McCray, N. Panagia, C. S. J. Pun, M. M. Phillips, G. Sonneborn, N. B. Suntzeff, The axisymmetric ejecta of supernova 1987A. Astrophys. J. 579, 671–677 (2002).

[87] J. C. Wheeler, I. Yi, P. Höflich, L. Wang, Asymmetric supernovae, pulsars, magnetars, and gamma-ray bursts. Astrophys. J. 537, 810–823 (2000).

[88] S. Akiyama, J. C. Wheeler, D. L. Meier, I. Lichtenstadt, The magnetorotational instability in core-collapse supernova explosions. Astrophys. J. 584, 954–970 (2003).

[89] J. C. Wheeler, J. R. Maund, S. M. Couch, The shape of Cas A. Astrophys. J. 677, 1091–1099 (2008).

[90] F. Patat, J. R. Maund, S. Benetti, M. T. Botticella, E. Cappellaro, A. Harutyunyan, M. Turatto, VLT spectropolarimetry of the optical transient in NGC 300. Astron. Astrophys. 510, A108 (2010).

[91] L. Dessart, D. C. Leonard, D. J. Hillier, G. Pignata, Multiepoch VLT-FORS spectropolarimetric observations of supernova 2012aw reveal an asymmetric explosion. Astron. Astrophys. 651, A19 (2021).

[92] H. F. Stevance, J. R. Maund, D. Baade, P. Höflich, S. Howerton, F. Patat, M. Rose, J. Spyromilio, J. C. Wheeler, L. Wang, The evolution of the 3D shape of the broad-lined Type Ic SN 2014ad. Mon. Not. R. Astron. Soc. 469, 1897–1911 (2017).

[93] Y. Yang, D. Baade, P. Hoeflich, L. Wang, A. Cikota, T.-W. Chen, J. Burke, D. Hiramatsu, C. Pellegrino, D. A. Howell, C. McCully, S. Valenti, S. Schulze, A. Gal-Yam, L. Wang, A. V. Filippenko, K. Maeda, M. Bulla, Y. Yao, J. R. Maund, F. Patat, J. Spyromilio, J. C. Wheeler, A. Rau, L. Hu, W. Li, J. E. Andrews, L. Galbany, D. J. Sand, M. Shahbandeh, E. Y. Hsiao, X. Wang, The interaction of supernova 2018evt with a substantial amount of circumstellar matter - An SN 1997cy-like event. Mon. Not. R. Astron. Soc. 519, 1618–1647 (2022).

[94] D. A. Howell, P. Höflich, L. Wang, J. C. Wheeler, Evidence for asphericity in a subluminous Type Ia supernova: Spectropolarimetry of SN 1999by. Astrophys. J. 556, 302–321 (2001).

[95] N. Mandarakas, K. Tassis, R. Skalidis, 3D interstellar medium structure challenges the Serkowski relation. Astron. Astrophys. 698, A168 (2025).

[96] I. Appenzeller, K. J. Fricke, W. Fürtig, W. Gassler, R. M. Hafner, R. Harke, W. Hummel, P. Jürgens, W. Meisl, B. Muschielok, H. E. Nicklas, G. Rupprecht, W. Seifert, O. Stahl, T. Szeifert, K. Tarantik, Successful commissioning of FORS1 – The first optical instrument on the VLT. Messenger 94, 1–6 (1998).

[97] J. Anderson, Very Large Telescope Paranal Science Operations FORS2 User Manual (European Southern Observatory Doc. No. VLT-MAN-ESO-13100-1543, 2018).

[98] D. Tody, in *Instrumentation in Astronomy VI*, D. L. Crawford, Ed. (SPIE, 1986), vol. 627, p. 733.

[99] D. Tody, in *Astronomical Data Analysis Software and Systems II*, R. J. Hanisch, R. J. V. Brissenden, J. Barnes, Eds. (Astronomical Society of the Pacific, 1993), vol. 52, p. 173.

[100] F. Patat, M. Romaniello, Error analysis for dual-beam optical linear polarimetry. Publ. Astron. Soc. Pac. 118, 146–161 (2006).

[101] J. F. L. Simmons, B. G. Stewart, Point and interval estimation of the true unbiased degree of linear polarization in the presence of low signal-to-noise ratios. Astron. Astrophys. 142, 100–106 (1985).

[102] Y. Yang, P. Hoeflich, D. Baade, J. R. Maund, L. Wang, P. J. Brown, H. F. Stevance, I. Arcavi, J. Burke, A. Cikota, A. Clocchiatti, A. Gal-Yam, M. L. Graham, D. Hiramatsu, G. Hosseinzadeh, D. A. Howell, S. W. Jha, C. McCully, F. Patat, D. J. Sand, S. Schulze, J. Spyromilio, S. Valenti, J. Vinkó, X. Wang, J. C. Wheeler, O. Yaron, J. Zhang, The young and nearby normal Type Ia supernova 2018gv: UV-optical observations and the earliest spectropolarimetry. Astrophys. J. 902, 46 (2020).

[103] A. Cikota, F. Patat, S. Cikota, T. Faran, Linear spectropolarimetry of polarimetric standard stars with VLT/FORS2. arXiv:1610.00722 (astro-ph.IM) (2017).

[104] B. S. Koribalski, L. Staveley-Smith, V. A. Kilborn, S. D. Ryder, R. C. Kraan-Korteweg, E. V. Ryan-Weber, R. D. Ekers, H. Jerjen, P. A. Henning, M. E. Putman, M. A. Zwaan, W. J. G. de Blok, M. R. Calabretta, M. J. Disney, R. F. Minchin, R. Bhathal, P. J. Boyce, M. J. Drinkwater, K. C. Freeman, B. K. Gibson, A. J. Green, R. F. Haynes, S. Juraszek, M. J. Kesteven, P. M. Knezek, S. Mader, M. Marquarding, M. Meyer, J. R. Mould, T. Oosterloo, J. O’Brien, R. M. Price, E. M. Sadler, A. Schröder, I. M. Stewart, F. Stootman, M. Waugh, B. E. Warren, R. L. Webster, A. E. Wright, The 1000 brightest HIPASS galaxies: HI properties. Astrophys. J. 128, 16–46 (2004).

[105] N. N. Chugai, Broad emission lines from the opaque electron-scattering environment of SN 1998S. arXiv:astro-ph/0106234 (astro-ph) (2001).

[106] L. Dessart, D. J. Hillier, S. Gezari, S. Basa, T. Matheson, SN 1994W: An interacting supernova or two interacting shells? Mon. Not. R. Astron. Soc. 394, 21–37 (2009).

[107] C. Huang, R. A. Chevalier, Electron scattering wings on lines in interacting supernovae. Mon. Not. R. Astron. Soc. 475, 1261–1273 (2018).

[108] D. Kasen, D. Branch, E. Baron, D. Jeffery, A complete analytic inversion of supernova lines in the Sobolev approximation. Astrophys. J. 565, 380–384 (2002).

[109] S. Chandrasekhar, On the radiative equilibrium of a stellar atmosphere. X. Astrophys. J. 103, 351 (1946).

[110] D. Branch, D. J. Jeffery, M. Blaylock, K. Hatano, Supernova resonance-scattering profiles in the presence of external illumination. Publ. Astron. Soc. Pac. 112, 217–223 (2000).

[111] X. Wen, H. Gao, S. Ai, L.-D. Liu, J.-P. Zhu, W.-H. Lei, Polarization signature of companion-fed supernovae arising from BH-NS/BH progenitor systems. Astrophys. J. 955, 9 (2023).

[112] A. D. Code, B. A. Whitney, Polarization from scattering in blobs. Astrophys. J. 441, 400 (1995).

[113] P. Hoflich, Asphericity effects in scatterring dominated photospheres. Astron. Astrophys. 246, 481 (1991).

[114] L. B. Lucy, Monte Carlo techniques for time-dependent radiative transfer in 3-D supernovae. Astron. Astrophys. 429, 19–30 (2005).

[115] M. Bulla, S. A. Sim, M. Kromer, Polarization spectral synthesis for Type Ia supernova explosion models. Mon. Not. R. Astron. Soc. 450, 967–981 (2015).

[116] S. Chandrasekhar, *Radiative Transfer* (Dover Publications, 1960).

[117] L. Dessart, D. J. Hillier, Quantitative spectroscopy of photospheric-phase type II supernovae. Astron. Astrophys. 437, 667–685 (2005).

[118] M. L. McCall, Are supernovae round? I - The case for spectropolarimetry. Mon. Not. R. Astron. Soc. 210, 829–837 (1984).

[119] A. Burrows, D. Vartanyan, Core-collapse supernova explosion theory. Nature 589, 29–39 (2021). 33408377 10.1038/s41586-020-03059-w

[120] P. Höflich, A. Khokhlov, L. Wang, in *20th Texas Symposium on Relativistic Astrophysics*, J. C. Wheeler, H. Martel, Eds. (American Institute of Physics, 2001), vol. 586, pp. 459–471.

[121] Gaia Collaboration, VizieR Online Data Catalog: Gaia EDR3 (Gaia Collaboration, 2020).

[122] J. A. Cardelli, G. C. Clayton, J. S. Mathis, The relationship between infrared, optical, and ultraviolet extinction. Astrophys. J. 345, 245 (1989).

[123] E. F. Schlafly, D. P. Finkbeiner, Measuring reddening with Sloan Digital Sky Survey stellar spectra and recalibrating SFD. Astrophys. J. 737, 103 (2011).

