# Supplementary material for: An axisymmetric shock breakout indicated by prompt polarized emission from the type II supernova 2024ggi
Source: Sci Adv. 2025 Nov 12;11(46):eadx2925. doi: 10.1126/sciadv.adx2925 (PMC13142702; doi:10.1126/sciadv.adx2925)
Supplement: Supplementary file 1 — Supplementary Text Figs. S1 to S21 References [file sciadv.adx2925_sm.pdf]

Science Advances
AAAS

Supplementary Materials for

An axisymmetric shock breakout indicated by prompt polarized emission from the type II supernova 2024ggi

Yi Yang *et al.*

Corresponding author: Yi Yang, yi_yang@mail.tsinghua.edu.cn; Lifan Wang, lifan@tamu.edu; Xiaofeng Wang, wang_xf@mail.tsinghua.edu.cn

Sci. Adv. **11**, eadx2925 (2025)
DOI: 10.1126/sciadv.adx2925

This PDF file includes:

Supplementary Text
Figs. S1 to S21
References

Supplementary Text

As a sanity check, we also attempted to estimate the Galactic ISP caused by Galactic extinction with observations of the "probe star" CD328000 ($\alpha = 11 : 18 : 10.676$, $\delta = -32 : 51 : 10.729$; J2000), which is $143''.85$ W and $55''.43$ S of SN 2024ggi and has a distance of 1081 ± 21 pc from the Sun as measured by Gaia (*121*). The 3D extinction map estimates the Galactic reddening toward CD328000, $E(B-V)^{\rm Map} = 0.09^{+0.01}_{-0.02}$ mag (*122*, *123*), which is smaller than the Galactic extinction $E(B-V)_{\rm Gal} = 0.120 \pm 0.028$ mag (*52*) estimated by adding the reddening derived from the two Na I D1 and D2 components from the rest velocity ($E(B-V)_{\rm Gal} = 0.054 \pm 0.020$ mag) and a Galactic intervening cloud at redshift $z = 0.00039$ ($E(B-V)_{\rm Cloud} = 0.066 \pm 0.020$).

In fig. S2 we present VLT spectropolarimetry of CD328000, as a sanity check for the Galactic component of the ISP. The error-weighted mean values of the continuum polarization calculated for different wavelength ranges are all consistent. Their combined value is ($Q^{\rm ISP}_{\rm probe}$, $U^{\rm ISP}_{\rm probe}$) = ($-0.20\% \pm 0.07\%$, $+0.08\% \pm 0.08\%$). If the total ISP is proportional to the ratio between the total Galactic extinction along the SN-Earth line of sight and the extinction measured from the probe star (assuming it to be intrinsically unpolarized which is the case for most single stars), the total Galactic ISP of SN 2024ggi will then amount to $(Q^{\rm ISP}_{\rm MW}, U^{\rm ISP}_{\rm MW}) = (Q^{\rm ISP}_{\rm probe}, U^{\rm ISP}_{\rm probe}) \times E(B-V)^{\rm MW}/E(B-V)^{\rm Map} = (Q^{\rm ISP}_{\rm probe}, U^{\rm ISP}_{\rm probe}) \times 1.508 \pm 0.389 = [-0.30\% \pm 0.14\%, 0.12\% \pm 0.12\%]$.

The estimated Galactic ISP component is consistent with an empirical relation between extinction and dichroic extinction-induced polarization, $p_{\rm ISP}[\%] < 9\% \times E(B-V)$, which has been found for dust in the Milky Way (*56*). However, while different reddening components can be added because they are scalar quantities, this is not true for the ISP values because polarization is a pseudovector. We also assume that both the Galactic and the SN 2024ggi host dust follow a similar $R_V = 3.1$ reddening law (*122*). Depending on the orientation difference between the ISP in the host galaxy and the MW, we estimate a range of the total ISP toward SN 2024ggi as $-0.38\% \pm 0.17\% < Q^{\rm ISP} < -0.21\% \pm 0.10\%$, $0.09\% \pm 0.09\% < U^{\rm ISP} < 0.16\% \pm 0.16\%$.

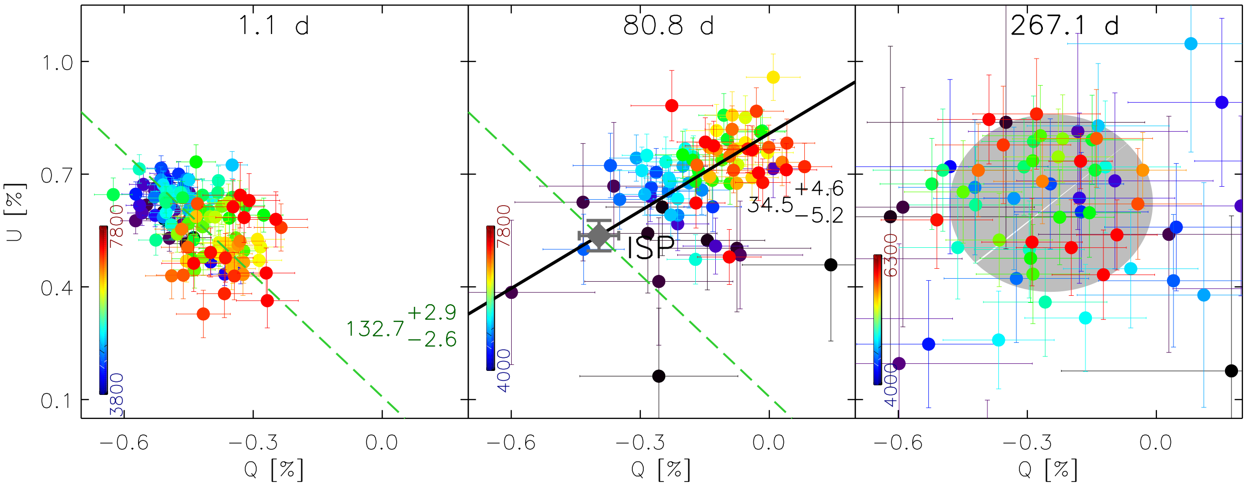

Figure S1: **Estimation of the interstellar polarization.** Small filled circles show the measurements in 30 Å bins, and their colors identify the wavelengths according to the color bar. The green dashed and the black solid lines in the left and the middle panels fit the dominant axes to the observed polarization in the wavelength range 3800–7800 Å on days 1.1 and 80.8, respectively. The filled gray circle (with 1σ error bars) in the middle panel marks the estimated ISP: $Q_{\mathrm{ISP}} = -0.40 \pm 0.05$ %, $U_{\mathrm{ISP}} = 0.54 \pm 0.04$ %. The right panel shows the polarization at day 267.1, when SN 2024ggi has entered the nebular phase and the emission is dominated by a blend of intrinsically unpolarized Fe-group features. The ISP estimated by the error-weighted mean polarization within 4000–6300 Å gives $Q_{\mathrm{ISP}}^{+267\,\mathrm{d}} = -0.25 \pm 0.24$ %, $U_{\mathrm{ISP}}^{+267\,\mathrm{d}} = 0.62 \pm 0.24$ % as indicated by the gray-shaded ellipse.

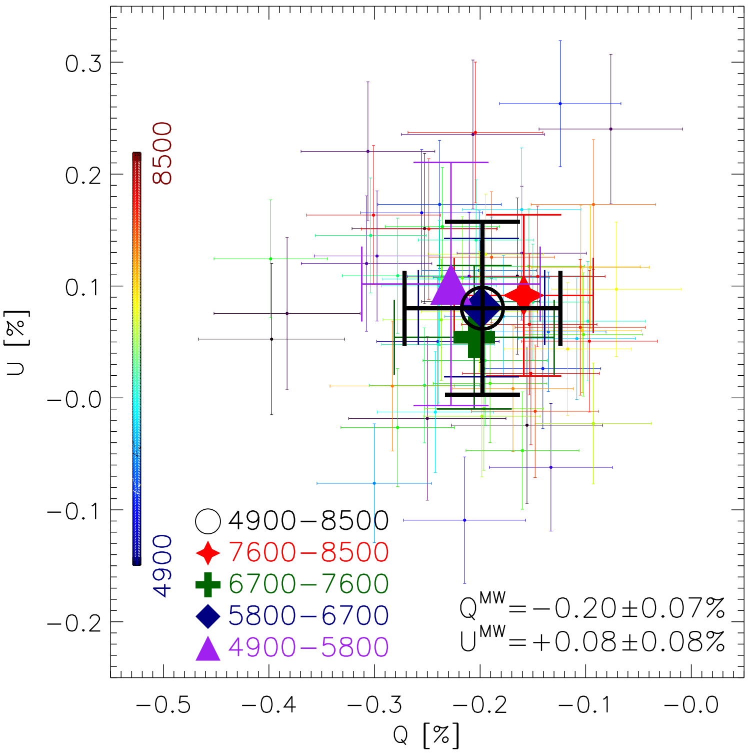

Figure S2: **Polarization of the Galactic ISP probe star.** Four filled symbols represent the weighted-mean polarization in four different wavelength ranges (as labeled). The small dots show the measurements in 50 Å bins, and their colors identify the wavelengths according to the color bar. The black circle (with 1σ error bars) marks the overall ISP, the Q and U values of which appear in the legend.

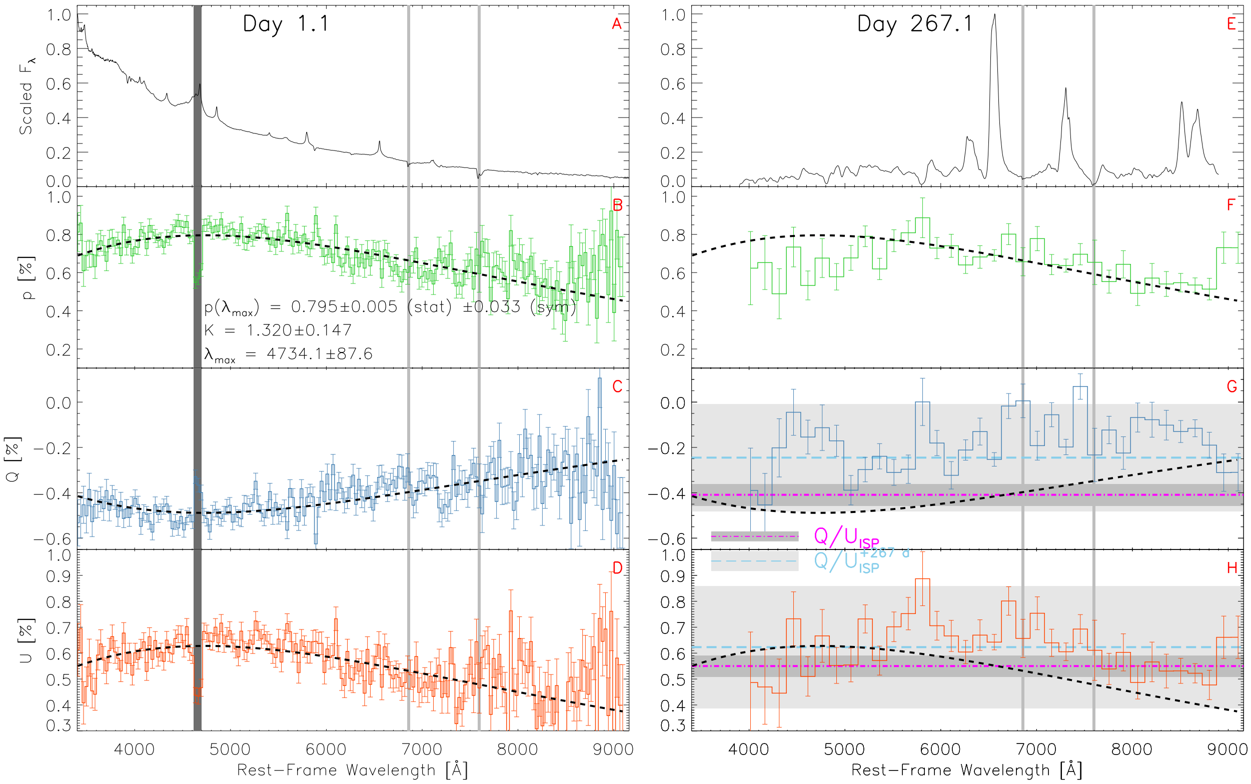

Figure S3: **Fitting the day 1.1 spectropolarimetry of SN 2024ggi with a Serkowski law.** Panel **A** shows the flux spectrum at day 1.1 normalized to the maximum value within the observed spectral range. Panels **B–D** present the degree of linear polarization, the Stokes Q and U for the same epoch, respectively, with the black dashed curve indicating the best fit using a Serkowski law. The result parameters are also labeled. The first and the second error terms in $p(\lambda_{\rm max})$ denote the statistical uncertainty due to the Serkowski-law fitting and the systematic uncertainty as represented by the median error in the p spectrum at day 1.1, respectively. The data have been rebinned to 30 Å. The region of the dark-gray-shaded band suffers from detector saturation and has been excluded from the fitting. The right panel shows the observation on day 267.1 and compares the polarization with the best-fit Serkowski law on day 1.1. The former exhibits significant departures from the latter, indicating the wavelength-dependent polarization at day 1.1 is not caused by the ISP. The data have been rebinned to 150 Å for clarity. The horizontal gray and light gray-shaded bands overlay the ISP estimated from the intersection of the dominant axes on days 1.1 and 80.8 and the nebular phase data at day 267.1, respectively.

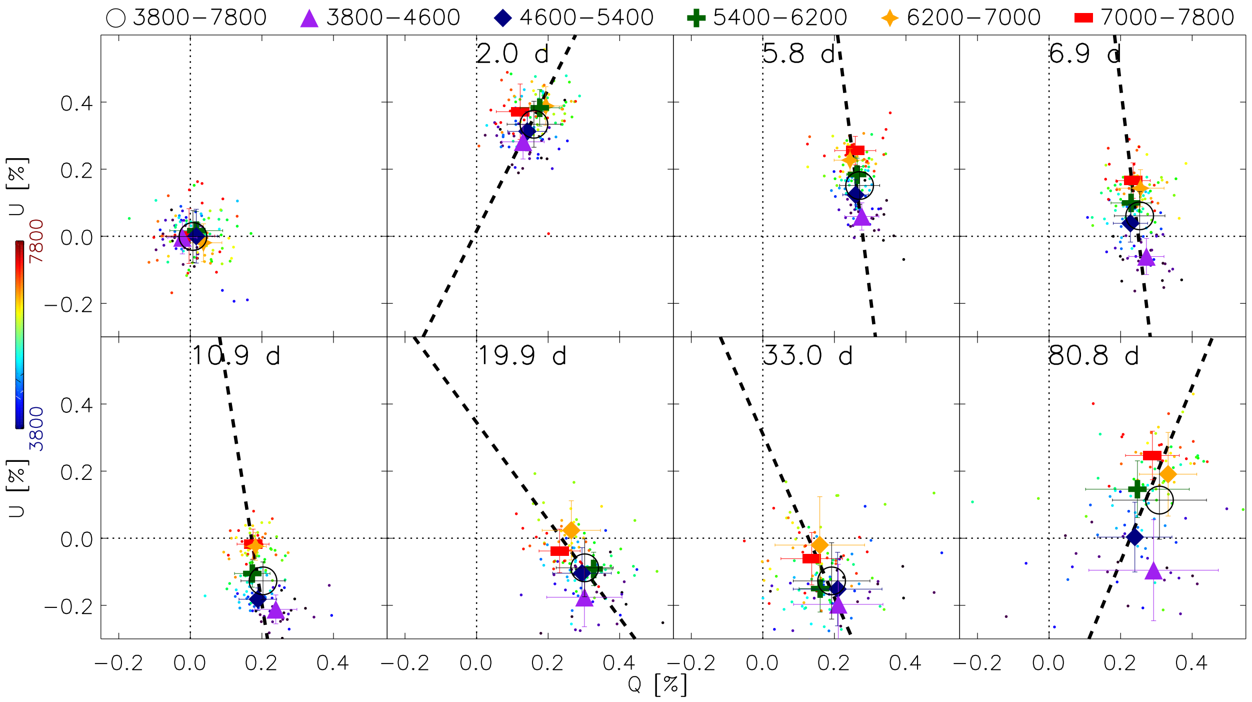

Figure S4: **Reproduction of Fig. 1 after removing the wavelength dependence of the day 1.1 spectropolarimetry.** By attributing the latter to the ISP that follows a Serkowski law described in fig. S3, the subtraction would introduce wavelength-dependent polarization at all other epochs as indicated by the best fit to the data shown by the black dashed lines.

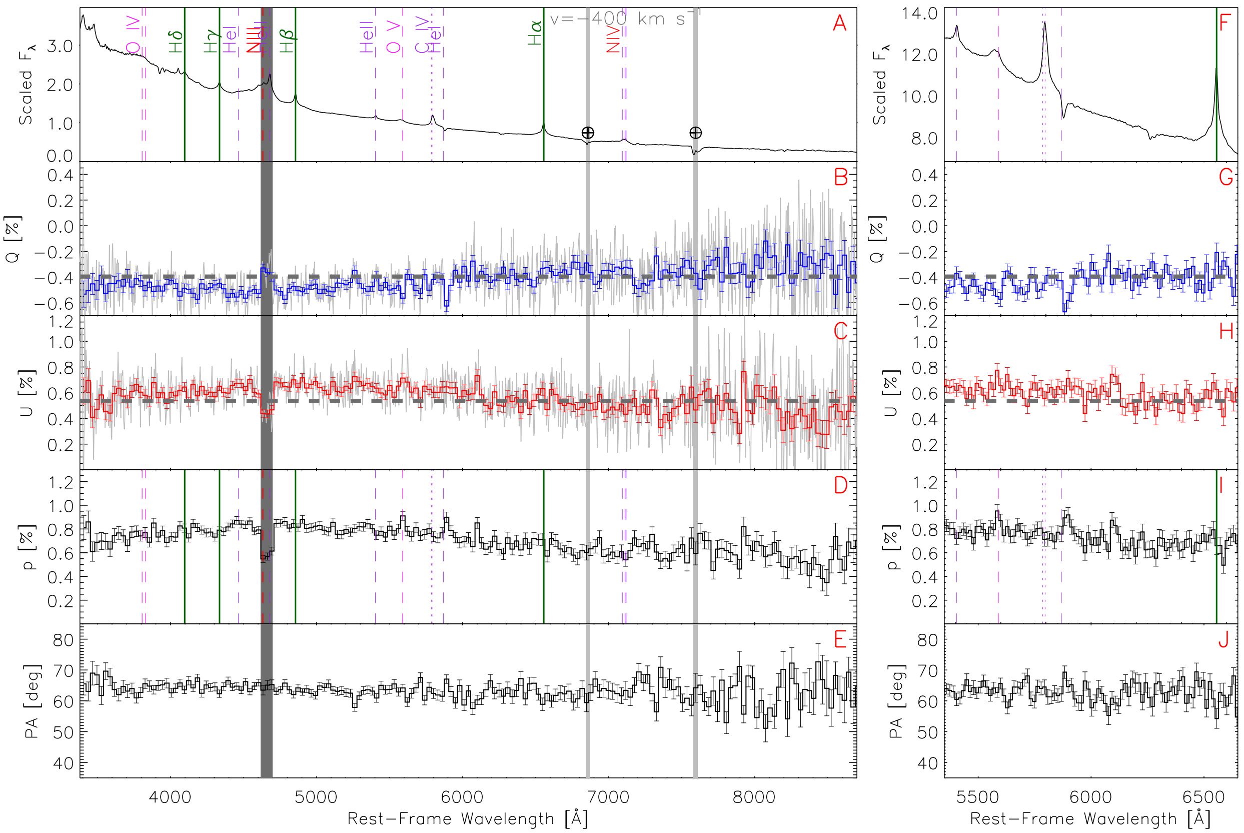

Figure S5: **Spectropolarimetry of SN 2024ggi on day 1.1 (epoch 1).** The five panels on the left (from top to bottom) display (**A**) the arbitrarily scaled total-flux spectrum with major spectral features identified; (**B**, **C**) the intensity-normalized Stokes parameters Q and U, respectively, with the level of ISP indicated by the horizontal gray dashed lines; (**D**) the polarization spectrum (p); and (**E**) the polarization position angle. Panels (**B**)–(**E**) represent the polarimetry before ISP correction, using 30 Å bins for clarity. The light-gray-shaded vertical bands identify regions of telluric contamination while the region of the dark-gray-shaded band suffers from detector saturation. The panels on the right (**F**)–(**J**) repeat some of the data in the left panels at higher resolution using 15 Å bins.

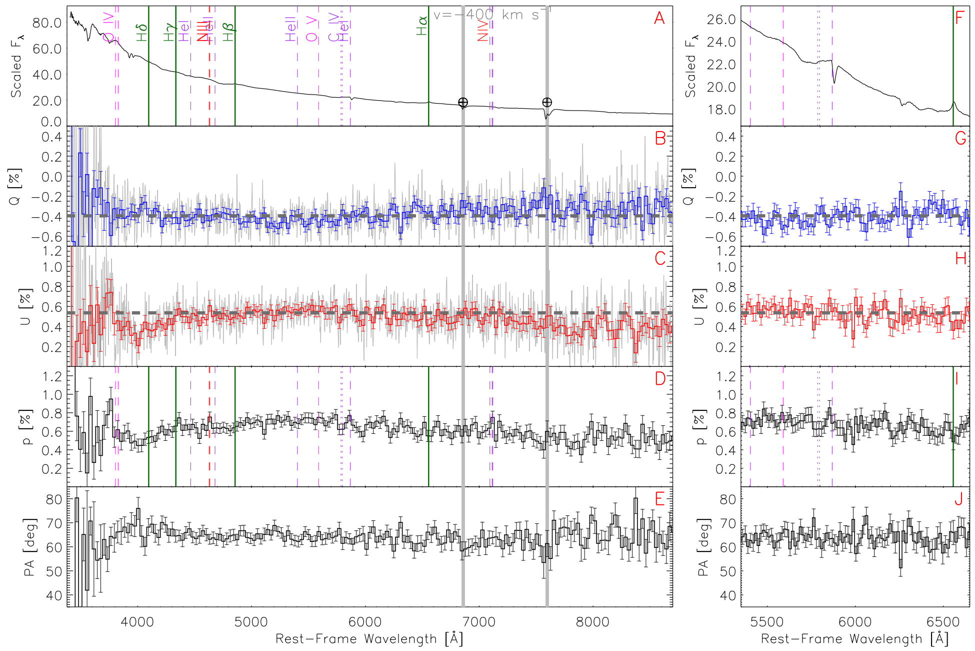

Figure S6: **Spectropolarimetry of SN 2024ggi on day 2.0 (epoch 2).** The layout is the same as that of fig. S5, namely the Stokes I, Q, U, p, and PA, from top to bottom rows, respectively.

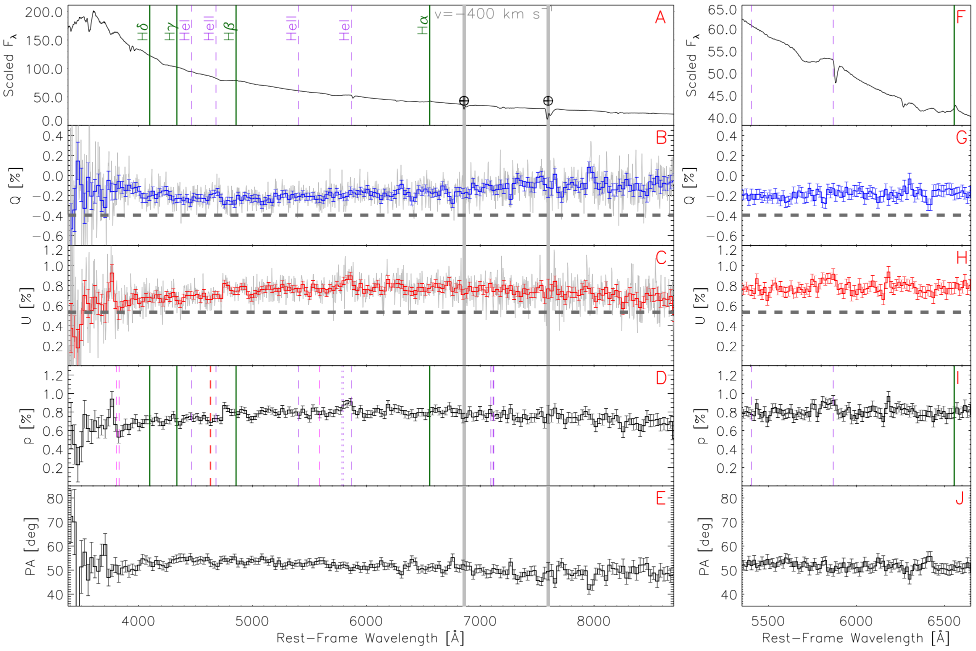

Figure S7: **Spectropolarimetry of SN 2024ggi on day 5.8 (epoch 3).** The layout is the same as that of fig. S5, namely the Stokes I, Q, U, p, and PA, from top to bottom rows, respectively.

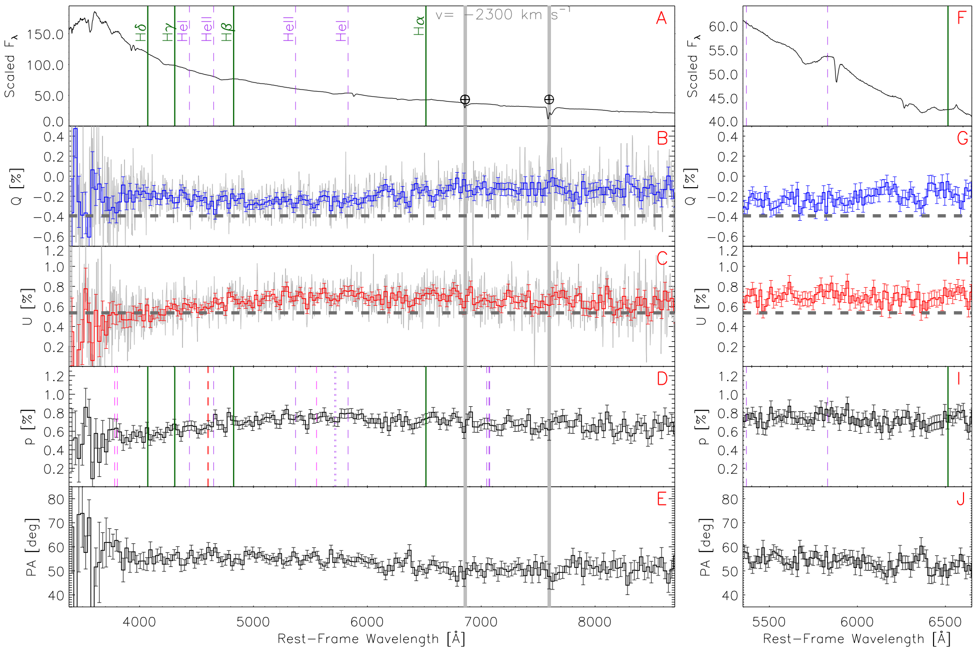

Figure S8: **Spectropolarimetry of SN 2024ggi on day 6.9 (epoch 4).** The layout is the same as that of fig. S5, namely the Stokes I, Q, U, p, and PA, from top to bottom rows, respectively.

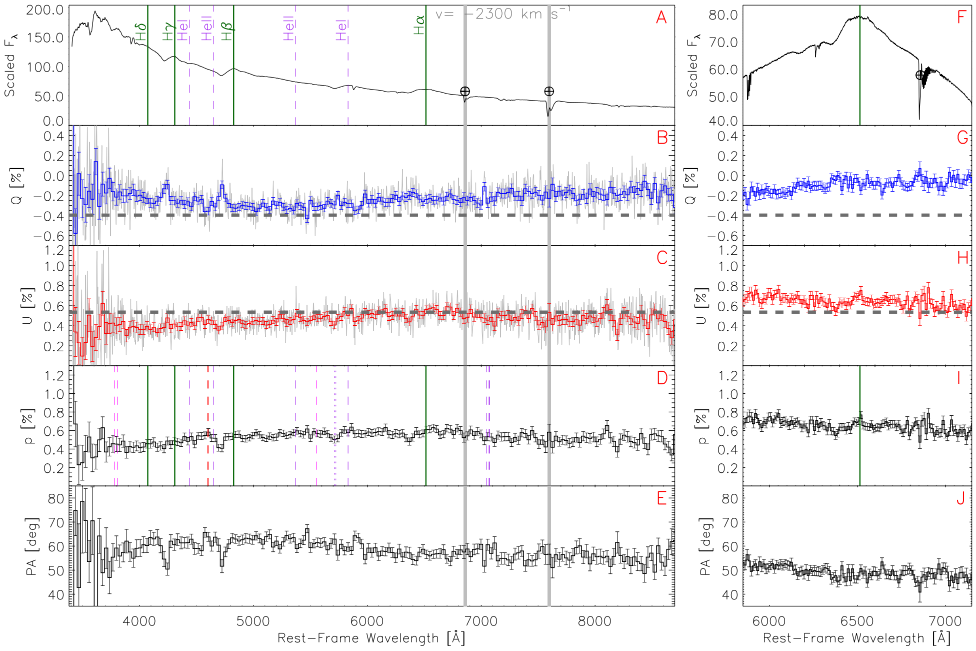

Figure S9: **Spectropolarimetry of SN 2024ggi on day 10.9 (epoch 5).** The layout is the same as that of fig. S5, namely the Stokes I, Q, U, p, and PA, from top to bottom rows, respectively.

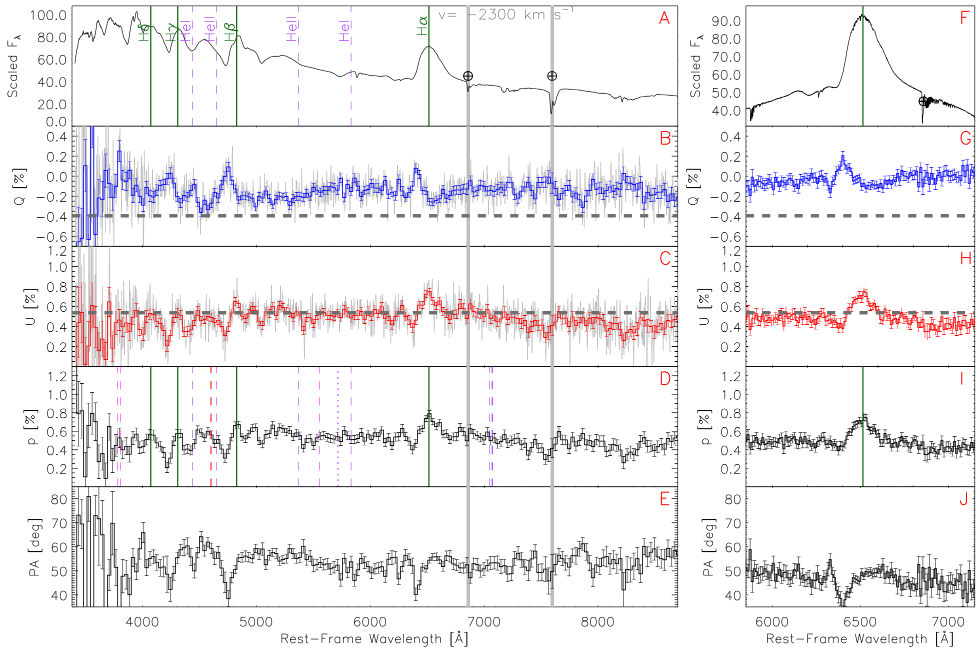

Figure S10: **Spectropolarimetry of SN 2024ggi on day 19.9 (epoch 6).** The layout is the same as that of fig. S5, namely the Stokes I, Q, U, p, and PA, from top to bottom rows, respectively.

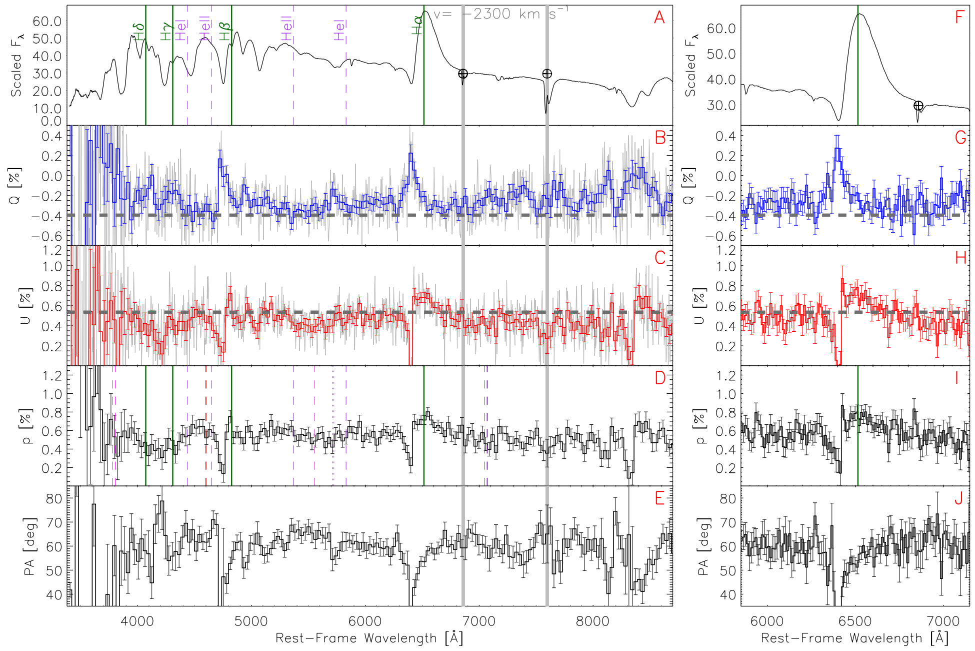

Figure S11: **Spectropolarimetry of SN 2024ggi on day 33.0 (epoch 7).** The layout is the same as that of fig. S5, namely the Stokes I, Q, U, p, and PA, from top to bottom rows, respectively.

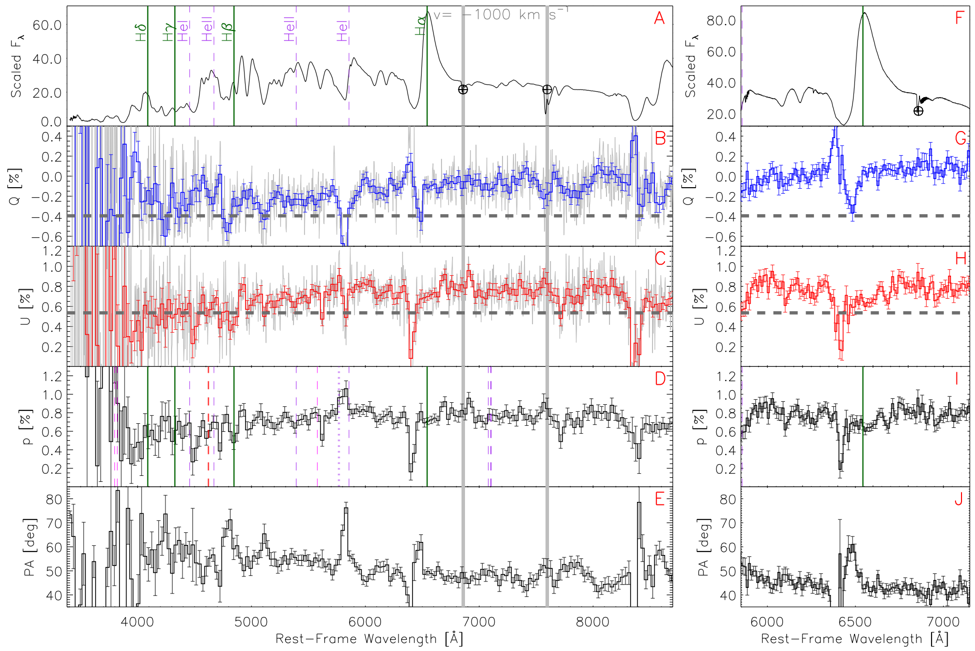

Figure S12: **Spectropolarimetry of SN 2024ggi on day 80.8 (epoch 8).** The layout is the same as that of fig. S5, namely the Stokes I, Q, U, p, and PA, from top to bottom rows, respectively.

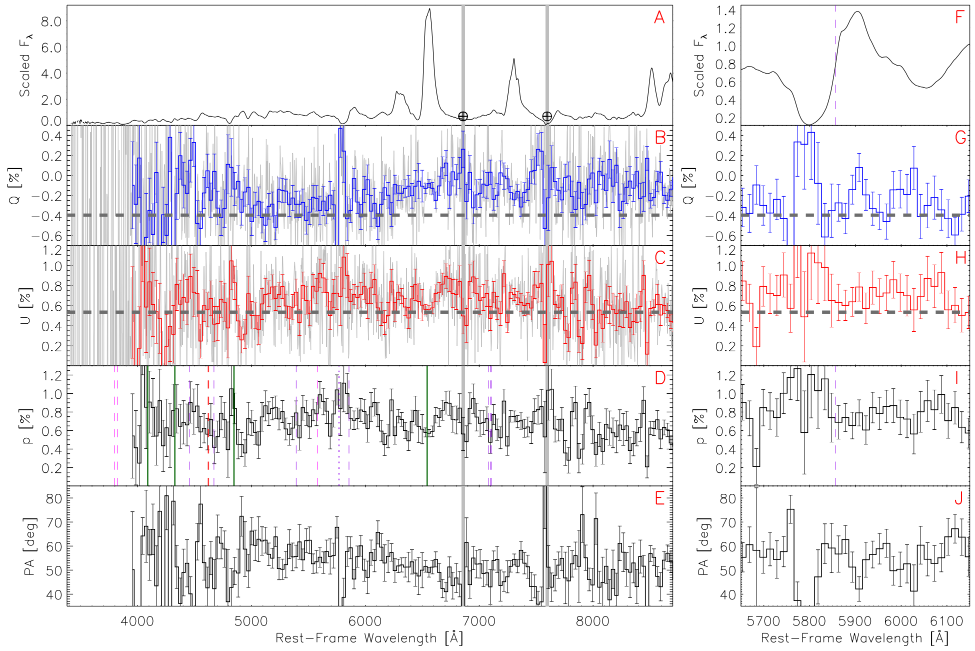

Figure S13: **Spectropolarimetry of SN 2024ggi on day 267.1 (epoch 9).** The layout is the same as that of fig. S5, namely the Stokes I, Q, U, p, and PA, from top to bottom rows, respectively.

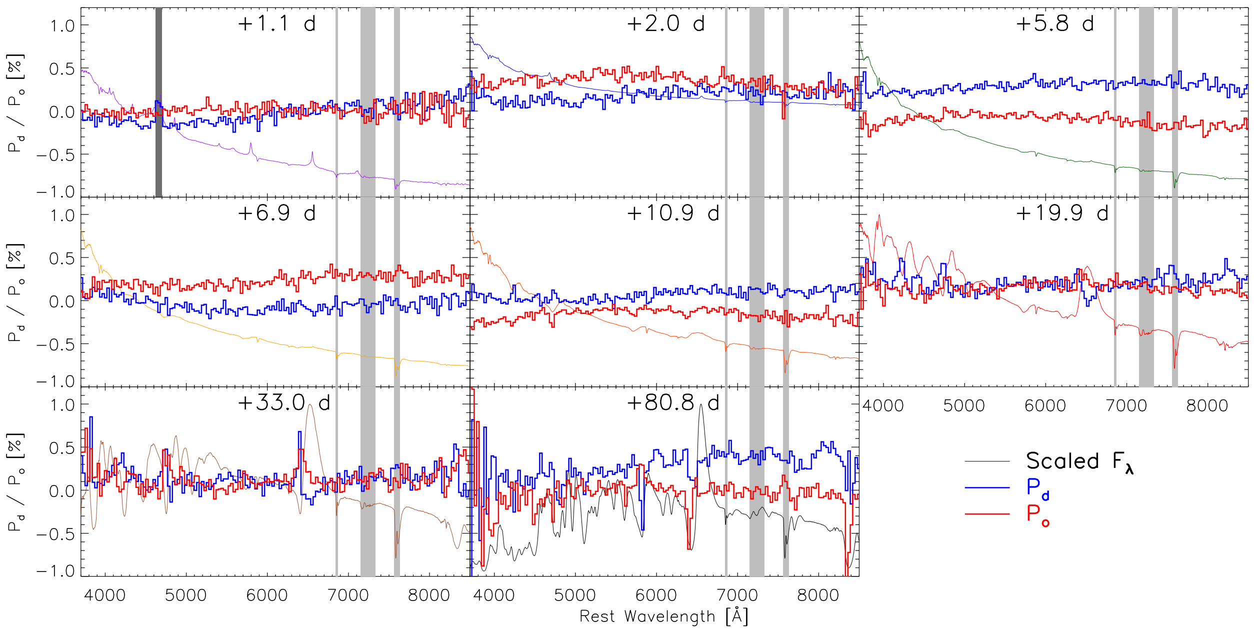

Figure S14: **Principal-components decomposition of the SN 2024ggi spectropolarimetry obtained between days 1.1 and 80.8.** In each panel, the color-coded line represents the arbitrarily scaled total-flux spectrum. The blue and the red histograms present the polarization spectrum projected onto the dominant (P_d) and the orthogonal axes (P_o), respectively, fitted across the entire observed wavelength range.

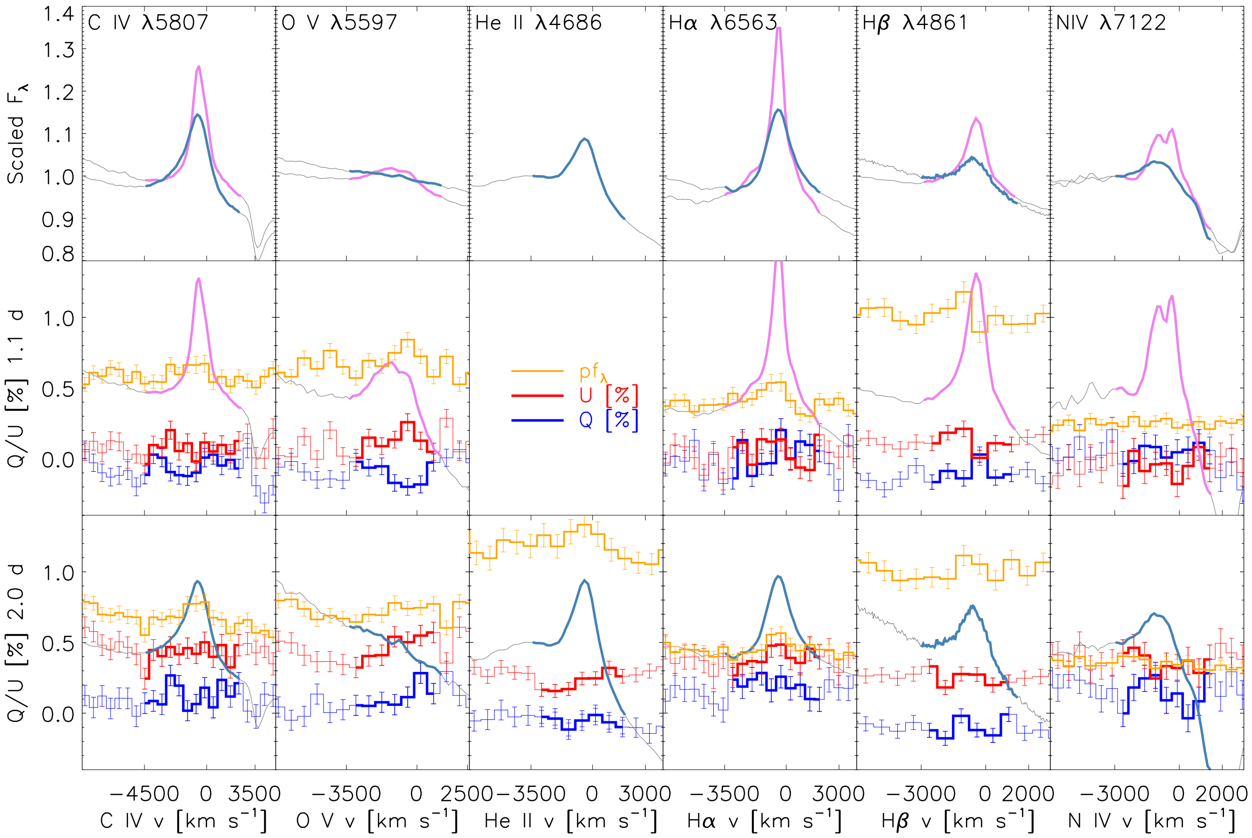

Figure S15: **Portrait gallery of the early-phase photoionized spectral features in Fig. 3.** The top row compares the scaled flux profiles (Stokes *I*) on days 1.1 (violet lines) and 2.0 (steel-blue lines). The spectral lines are identified above the top panels. The middle and bottom rows present the measurements of Stokes *Q* (blue histograms) and *U* (red histograms), respectively, for day 1.1 (middle row) and day 2.0 (bottom row) with arbitrarily scaled Stokes *I* overlaid. The orange histogram traces the polarized flux density $p \times f_\lambda$, which displays no significant deviation from the adjacent continuum. The color-coded wavelength segments identify the ranges over which the dominant axes of the continuum polarization shown in Fig. 3 have been fitted in the Stokes $Q - U$ plane.

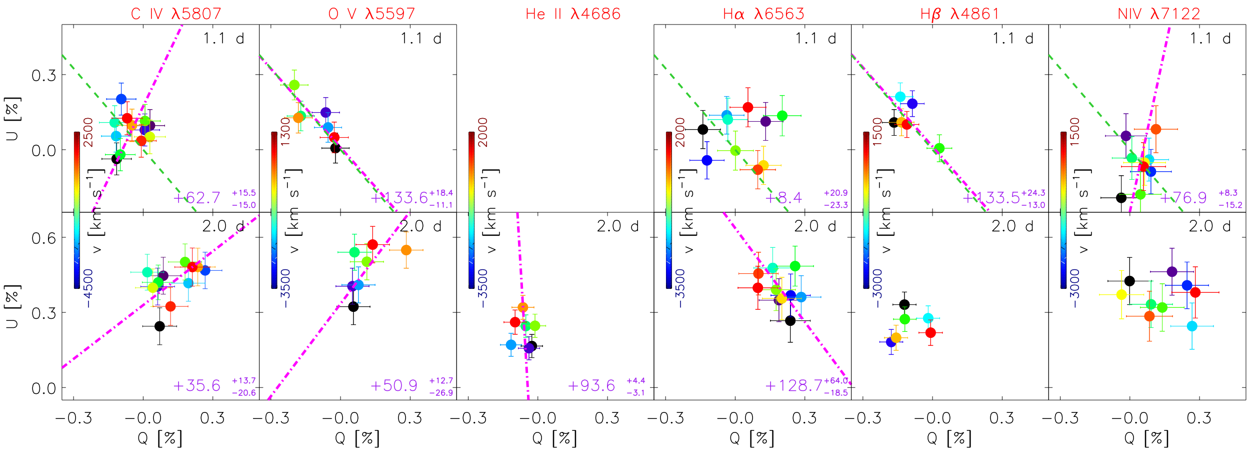

Figure S16: **Evolution of the line polarization of SN 2024ggi within two days of the discovery.** $Q-U$ plots with a 15 Å binning adopted are shown for five spectral lines as labeled and days 1.1 (top) and 2.0 (bottom) after the discovery (on day 1.1, the He II λ4686 emission was saturated and thus excluded from the analysis of the line polarization). In each panel, the magenta dash-dotted line fits the polarization measured at different velocity intervals in the rest frame identified by color; note that the color bars have different velocity ranges. The green dashed lines are the dominant axes of the continuum polarization (copied from Fig. 1). In the top row (day 1.1), the dominant axes of the spectral features with high excitation potential (e.g., O V, Hβ) closely follow that of the continuum, while other lines, which are formed farther out in the ionization front, have different orientations.

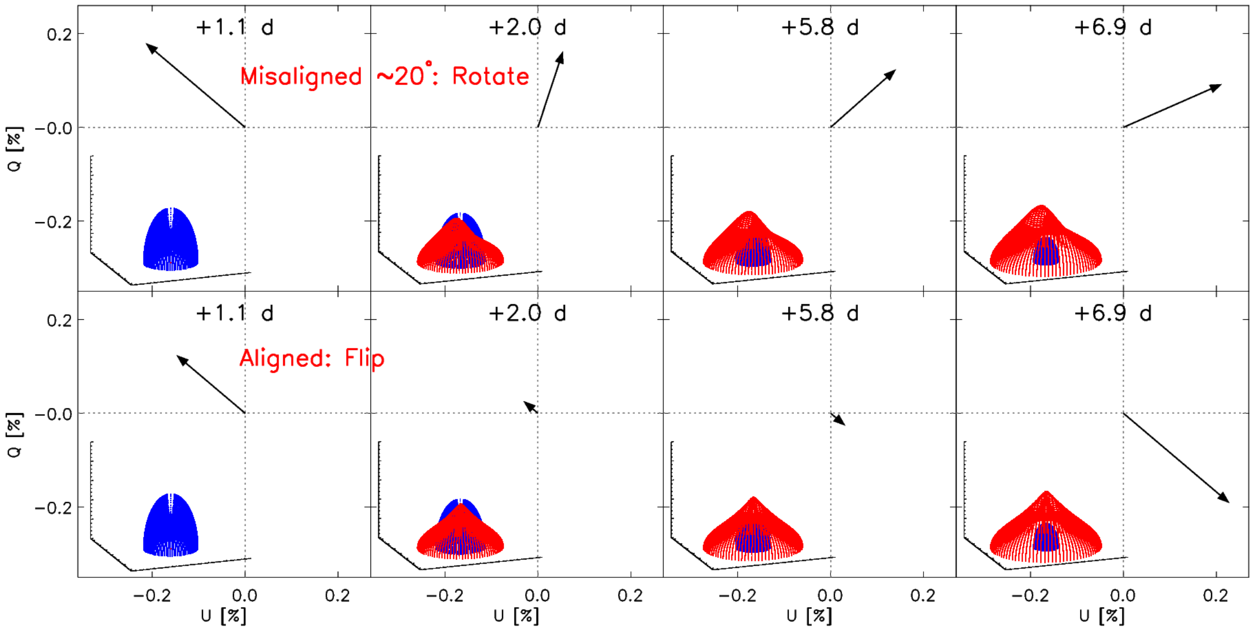

Figure S17: **Schematic illustration of the geometric prolate-to-oblate transition in one hemisphere that accounts for the clockwise rotation in the $Q-U$ plane of the dominant axis from days 1.1 to 6.9**. In each panel of the top row, the arrow points towards the direction of the continuum polarization computed from a linear combination of a prolate (blue) and an oblate (red) scattering atmosphere shown in the inset in the lower-left corner. The latter is tilted at an angle of 20° relative to the prolate structure and grows monotonically over time representing the way, in which the emission from the CSM concentrated in a plane becomes dominant. The bottom row illustrates the temporal evolution of the same quantity with the prolate and the oblate components aligned with each other, in which case the direction of the dominant axis exhibits a flip instead of a rotation. The relative strengths of the prolate and oblate emission components and the wavelengths were all arbitrarily assigned for illustration purposes.

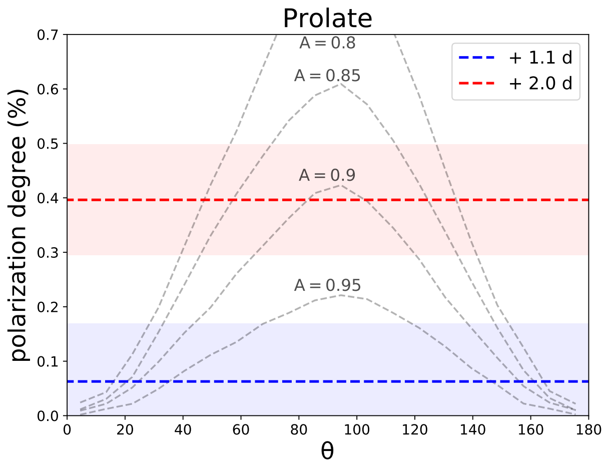

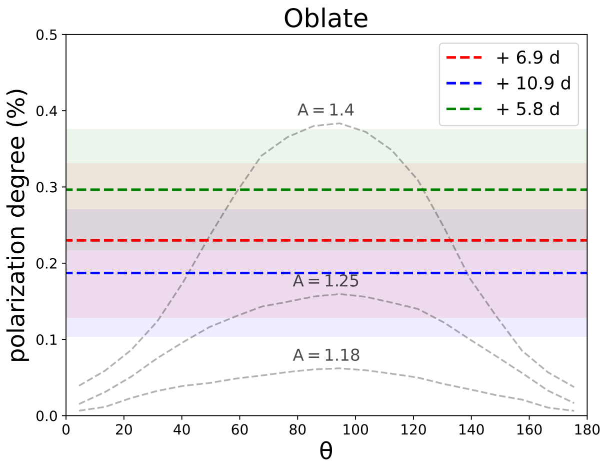

Figure S18: **Continuum polarization of a prolate geometry.** Results with $A = a/c < 1$ seen from different viewing angles θ are presented. The ejecta have a radial density structure of $\rho(r) \propto r^{-12}$. Photons are emitted from the photosphere with an optical depth $\tau = 1$.

Figure S19: **Continuum polarization of a oblate geometry.** Results with $A = a/c > 1$ seen from different viewing angles θ are presented. Calculations and layout are similar to that of fig. S18.

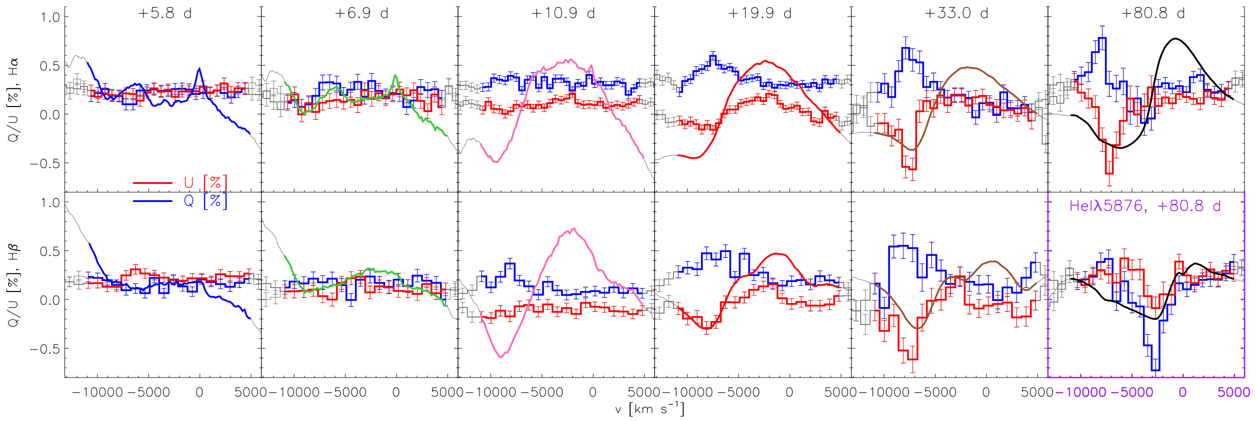

Figure S20: **Stokes $Q - U$ diagrams showing the Hα (top row) and Hβ features (bottom row) of SN 2024ggi.** The layout of the figure is similar to that of fig. S15 except for the epochs being days 5.8 (left column) to 33.0 (right column).

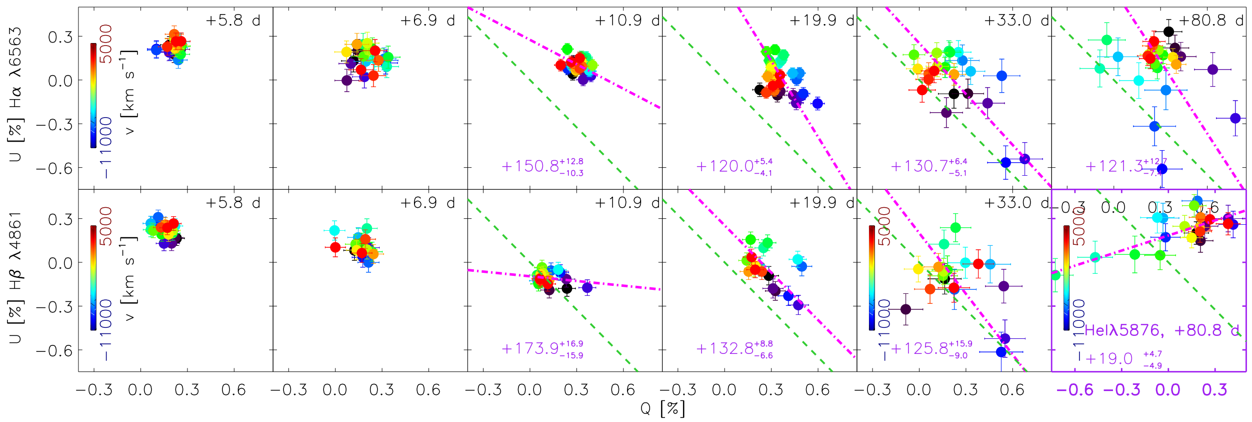

Figure S21: **Evolution of the Hα (top row) and Hβ (bottom row) polarization of SN 2024ggi from days 5.8 (left) to 80.8 (right column).** The colors encode velocities according to the color bars. In each panel, the magenta dot-dashed line fits the polarization distribution measured at different velocities that cover the corresponding spectral feature. The green dashed lines in the third to sixth columns overplot the dominant axis at day 1.1, which appear to be aligned with that of the H envelope that progressively emerged after day 6.9 (magenta dot-dashed line). The bottom-right panel presents the polarization across the He I λ5876 profile on day 80.8. The dominant axis fitted to this feature is misaligned with that shared by the prolate and the oblate configurations.

REFERENCES AND NOTES

1. T. D. Lee, S. Drell, Particle physics and introduction to field theory. *Phys. Today* **34**, 55–56 (1981).

2. L. Wang, J. C. Wheeler, Spectropolarimetry of supernovae. *Ann. Rev. Astron. Astrophys.* **46**, 433–474 (2008).

3. S. J. Smartt, J. J. Eldridge, R. M. Crockett, J. R. Maund, The death of massive stars - I. Observational constraints on the progenitors of Type II-P supernovae. *Mon. Not. R. Astron. Soc.* **395**, 1409–1437 (2009).

4. S. J. Smartt, Observational constraints on the progenitors of core-collapse supernovae: The case for missing high-mass stars. *Pub. Astron. Soc. Aust.* **32**, e016 (2015).

5. W. Li, J. Leaman, R. Chornock, A. V. Filippenko, D. Poznanski, M. Ganeshalingam, X. Wang, M. Modjaz, S. Jha, R. J. Foley, N. Smith, Nearby supernova rates from the Lick Observatory Supernova Search - II. The observed luminosity functions and fractions of supernovae in a complete sample. *Mon. Not. R. Astron. Soc.* **412**, 1441–1472 (2011).

6. A. Burrows, Colloquium: Perspectives on core-collapse supernova theory. *Rev. Mod. Phys.* **85**, 245 (2013).

7. A. Burrows, T. Wang, D. Vartanyan, Physical correlations and predictions emerging from modern core-collapse supernova theory. *Astrophys. J. Lett.* **964**, L16 (2024).

8. T. Wang, A. Burrows, Supernova explosions of the lowest-mass massive star progenitors. *Astrophys. J.* **969**, 74 (2024).

9. C. D. Ott, A. Burrows, T. A. Thompson, E. Livne, R. Walder, The spin periods and rotational profiles of neutron stars at birth. *Astrophys. J. Suppl. Ser.* **164**, 130–155 (2006).

10. A. Burrows, L. Dessart, E. Livne, C. D. Ott, J. Murphy, Simulations of magnetically driven supernova and hypernova explosions in the context of rapid rotation. *Astrophys. J.* **664**, 416–434 (2007).

11. J. M. LeBlanc, J. R. Wilson, A numerical example of the collapse of a rotating magnetized star. *Astrophys. J.* **161**, 541 (1970).

12. A. M. Khokhlov, P. A. Höflich, E. S. Oran, J. C. Wheeler, L. Wang, A. Y. Chtchelkanova, Jet-induced explosions of core collapse supernovae. *Astrophys. J.* **524**, L107–L110 (1999).

13. K. Maeda, K. Nomoto, Bipolar supernova explosions: Nucleosynthesis and implications for abundances in extremely metal-poor stars. *Astrophys. J.* **598**, 1163–1200 (2003).

14. M. Obergaulinger, H.-T. Janka, M. A. Aloy, Magnetic field amplification and magnetically supported explosions of collapsing, non-rotating stellar cores. *Mon. Not. R. Astron. Soc.* **445**, 3169–3199 (2014).

15. P. Mösta, C. D. Ott, D. Radice, L. F. Roberts, E. Schnetter, R. Haas, A large-scale dynamo and magnetoturbulence in rapidly rotating core-collapse supernovae. *Nature* **528**, 376–379 (2015).

16. H.-T. Janka, T. Melson, A. Summa, Physics of core-collapse supernovae in three dimensions: A sneak preview. *Ann. Rev. Nucl. Part. Sci.* **66**, 341–375 (2016).

17. B. Aschenbach, R. Egger, J. Trümper, Discovery of explosion fragments outside the Vela supernova remnant shock-wave boundary. *Nature* **373**, 587–590 (1995).

18. N. Soker, The role of jets in exploding supernovae and in shaping their remnants. *Res. Astron. Astrophys.* **22**, DOI:10.1088/1674-4527/ac9782 (2022).

19. A. G. Lyne, D. R. Lorimer, High birth velocities of radio pulsars. *Nature* **369**, 127–129 (1994).

20. B. M. S. Hansen, E. S. Phinney, The pulsar kick velocity distribution. *Mon. Not. R. Astron. Soc.* **291**, 569–577 (1997).

21. H.-T. Janka, Explosion mechanisms of core-collapse supernovae. *Ann. Rev. Nucl. Part. Sci.* **62**, 407–451 (2012).

22. H.-T. Janka, Neutron star kicks by the gravitational tug-boat mechanism in asymmetric supernova explosions: Progenitor and explosion dependence. *Astrophys. J.* **837**, 84 (2017).

23. T. M. Tauris, M. Kramer, P. C. C. Freire, N. Wex, H.-T. Janka, N. Langer, P. Podsiadlowski, E. Bozzo, S. Chaty, M. U. Kruckow, E. P. J. van den Heuvel, J. Antoniadis, R. P. Breton, D. J. Champion, Formation of double neutron star systems. *Astrophys. J.* **846**, 170 (2017).

24. P. Hoeflich, J. C. Wheeler, D. C. Hines, S. R. Trammell, Analysis of the polarization and flux spectra of SN 1993J. *Astrophys. J.* **459**, 307–321 (1996).

25. L. Wang, D. A. Howell, P. Höflich, J. C. Wheeler, Bipolar supernova explosions. *Astrophys. J.* **550**, 1030–1035 (2001).

26. J. R. Maund, J. C. Wheeler, D. Baade, F. Patat, P. Höflich, L. Wang, A. Clocchiatti, The early asymmetries of supernova 2008D/XRF 080109. *Astrophys. J.* **705**, 1139–1151 (2009).

27. A. Perego, M. Hempel, C. Fröhlich, K. Ebinger, M. Eichler, J. Casanova, M. Liebendörfer, F.-K. Thielemann, PUSHing core-collapse supernovae to explosions in spherical symmetry I: The model and the case of SN 1987A. *Astrophys. J.* **806**, 275 (2015).

28. E. O'Connor, R. Bollig, A. Burrows, S. Couch, T. Fischer, H.-T. Janka, K. Kotake, E. J. Lentz, M. Liebendörfer, O. E. B. Messer, A. Mezzacappa, T. Takiwaki, D. Vartanyan, Global comparison of core-collapse supernova simulations in spherical symmetry. *J. Phys. G Nucl. Part. Phys.* **45**, 104001 (2018).

29. S. M. Couch, M. L. Warren, E. P. O'Connor, Simulating turbulence-aided neutrino-driven core-collapse supernova explosions in one dimension. *Astrophys. J.* **890**, 127 (2020).

30. B. Müller, D. W. Gay, A. Heger, T. M. Tauris, S. A. Sim, Multidimensional simulations of ultrastripped supernovae to shock breakout. *Mon. Not. R. Astron. Soc.* **479**, 3675–3689 (2018).

31. D. Vartanyan, B.-T. Tsang, D. Kasen, A. Burrows, T. Wang, L. Teryoshin, A 3D simulation of a Type II-P supernova: From core bounce to beyond shock breakout. *Astrophys. J.* **982**, 9 (2025).

32. G. Li, M. Hu, W. Li, Y. Yang, X. Wang, S. Yan, L. Hu, J. Zhang, Y. Mao, H. Riise, X. Gao, T. Sun, J. Liu, D. Xiong, L. Wang, J. Mo, A. Iskandar, G. Xi, D. Xiang, L. Wang, G. Sun, K. Zhang, J. Chen, W. Lin, F. Guo, Q. Liu, G. Cai, W. Zhou, J. Zhao, J. Chen, X. Zheng, K. Li, M. Zhang, S. Xu, X. Lyu, A. J. Castro-Tirado, V. Chufarin, N. Potapov, I. Ionov, S. Korotkiy, S. Nazarov, K. Sokolovsky, N. Hamann, E. Herman, A shock flash breaking out of a dusty red supergiant. *Nature* **627**, 754–758 (2024).

33. A. Gal-Yam, I. Arcavi, E. O. Ofek, S. Ben-Ami, S. B. Cenko, M. M. Kasliwal, Y. Cao, O. Yaron, D. Tal, J. M. Silverman, A. Horesh, A. De Cia, F. Taddia, J. Sollerman, D. Perley, P. M. Vreeswijk, S. R. Kulkarni, P. E. Nugent, A. V. Filippenko, J. C. Wheeler, A WolfRayet-like progenitor of SN 2013cu from spectral observations of a stellar wind. *Nature* **509**, 471–474 (2014).

34. D. Khazov, O. Yaron, A. Gal-Yam, I. Manulis, A. Rubin, S. R. Kulkarni, I. Arcavi, M. M. Kasliwal, E. O. Ofek, Y. Cao, D. Perley, J. Sollerman, A. Horesh, M. Sullivan, A. V. Filippenko, P. E. Nugent, D. A. Howell, S. B. Cenko, J. M. Silverman, H. Ebeling, F. Taddia, J. Johansson, R. R. Laher, J. Surace, U. D. Rebbapragada, P. R. Wozniak, T. Matheson, Flash spectroscopy: Emission lines from the ionized circumstellar material around <10-day-old Type II supernovae. *Astrophys. J.* **818**, 1329868 (2016).

35. O. Yaron, D. A. Perley, A. Gal-Yam, J. H. Groh, A. Horesh, E. O. Ofek, S. R. Kulkarni, J. Sollerman, C. Fransson, A. Rubin, P. Szabo, N. Sapir, F. Taddia, S. B. Cenko, S. Valenti, I. Arcavi, D. A. Howell, M. M. Kasliwal, P. M. Vreeswijk, D. Khazov, O. D. Fox, Y. Cao, O. Gnat, P. L. Kelly, P. E. Nugent, A. V. Filippenko, R. R. Laher, P. R. Wozniak, W. H. Lee, U. D. Rebbapragada, K. Maguire, M. Sullivan, M. T. Soumagnac, Confined dense circumstellar material surrounding a regular type II supernova. *Nat. Phys.* **13**, 510–517 (2017).

36. L. Dessart, D. J. Hillier, E. Audit, Explosion of red-supergiant stars: Influence of the atmospheric structure on shock breakout and early-time supernova radiation. *Astron. Astrophys.* **605**, A83 (2017).

37. R. J. Bruch, A. Gal-Yam, S. Schulze, O. Yaron, Y. Yang, M. Soumagnac, M. Rigault, N. L. Strotjohann, E. Ofek, J. Sollerman, F. J. Masci, C. Barbarino, A. Y. Q. Ho, C. Fremling, D. Perley, J. Nordin, S. B. Cenko, S. Adams, I. Adreoni, E. C. Bellm, N. Blagorodnova, M.

Bulla, K. Burdge, K. De, S. Dhawan, A. J. Drake, D. A. Duev, A. Dugas, M. Graham, M. L. Graham, I. Irani, J. Jencson, E. Karamehmetoglu, M. Kasliwal, Y.-L. Kim, S. Kulkarni, T. Kupfer, J. Liang, A. Mahabal, A. A. Miller, T. A. Prince, R. Riddle, Y. Sharma, R. Smith, F. Taddia, K. Taggart, R. Walters, L. Yan, A large fraction of hydrogen-rich supernova progenitors experience elevated mass loss shortly prior to explosion. *Astrophys. J.* **912**, 46 (2021).

38. R. J. Bruch, A. Gal-Yam, O. Yaron, P. Chen, N. L. Strotjohann, I. Irani, E. Zimmerman, S. Schulze, Y. Yang, Y.-L. Kim, M. Bulla, J. Sollerman, M. Rigault, E. Ofek, M. Soumagnac, F. J. Masci, C. Fremling, D. Perley, J. Nordin, S. B. Cenko, A. Y. Q. Ho, S. Adams, I. Adreoni, E. C. Bellm, N. Blagorodnova, K. Burdge, K. De, R. G. Dekany, S. Dhawan, A. J. Drake, D. A. Duev, M. Graham, M. L. Graham, J. Jencson, E. Karamehmetoglu, M. M. Kasliwal, S. Kulkarni, A. A. Miller, J. D. Neill, T. A. Prince, R. Riddle, B. Rusholme, Y. Sharma, R. Smith, N. Sravan, K. Taggart, R. Walters, L. Yan, The prevalence and influence of circumstellar material around hydrogen-rich supernova progenitors. *Astron. Astrophys.* **952**, 119 (2023).

39. W. V. Jacobson-Galán, L. Dessart, K. W. Davis, C. D. Kilpatrick, R. Margutti, R. J. Foley, R. Chornock, G. Terreran, D. Hiramatsu, M. Newsome, E. P. Gonzalez, C. Pellegrino, D. A. Howell, A. V. Filippenko, J. P. Anderson, C. R. Angus, K. Auchettl, K. A. Bostroem, T. G. Brink, R. Cartier, D. A. Coulter, T. de Boer, M. R. Drout, N. Earl, K. Ertini, J. R. Farah, D. Farias, C. Gall, H. Gao, M. A. Gerlach, F. Guo, A. Haynie, G. Hosseinzadeh, A. L. Ibik, S. W. Jha, D. O. Jones, D. Langeroodi, N. LeBaron, E. A. Magnier, A. L. Piro, S. I. Raimundo, A. Rest, S. Rest, R. M. Rich, C. Rojas-Bravo, H. Sears, K. Taggart, V. A. Villar, R. J. Wainscoat, X.-F. Wang, A. R. Wasserman, S. Yan, Y. Yang, J. Zhang, W. Zheng, Final moments. II. Observational properties and physical modeling of circumstellar-material-interacting Type II supernovae. *Astrophys. J.* **970**, 189 (2024).

40. L. Wang, D. Baade, P. Höflich, A. Khokhlov, J. C. Wheeler, D. Kasen, P. E. Nugent, S. Perlmutter, C. Fransson, P. Lundqvist, Spectropolarimetry of SN 2001el in NGC 1448: Asphericity of a normal Type Ia supernova. *Astrophys. J.* **591**, 1110–1128 (2003).

41. D. Kasen, P. Nugent, L. Wang, D. A. Howell, J. C. Wheeler, P. Höflich, D. Baade, E. Baron, P. H. Hauschildt, Analysis of the flux and polarization spectra of the Type Ia supernova SN 2001el: Exploring the geometry of the high-velocity ejecta. *Astrophys. J.* **593**, 788–808 (2003).

42. S. Srivastav, T. W. Chen, S. J. Smartt, M. Nicholl, K. W. Smith, D. R. Young, M. Fulton, M. McCollum, T. Moore, J. Weston, X. Sheng, A. Aamer, C. R. Angus, P. Ramsden, L. Shingles, J. Gillanders, L. Rhodes, A. Andersson, H. Stevance, L. Denneau, J. Tonry, H. Weiland, A. Lawrence, R. Siverd, N. Erasmus, W. Koorts, A. Jordan, V. Suc, A. Rest, C. Stubbs, J. Sommer, ATLAS24fsk (AT2024ggi): Discovery of a nearby candidate SN in NGC 3621 at 7 Mpc with a possible progenitor detection. *Trans. Name Server AstroNote* **100**, 1 (2024).

43. A. Saha, F. Thim, G. A. Tammann, B. Reindl, A. Sandage, Cepheid distances to SNe Ia host galaxies based on a revised photometric zero point of the HST WFPC2 and new PL relations and metallicity corrections. *Astrophys. J. Suppl. Ser.* **165**, 108–137 (2006).

44. Q. Zhai, L. Li, Z. Wang, J. Zhang, X. Wang, LJT spectroscopic classification of AT 2024ggi as a young Type II supernova with flash features. *Trans. Name Server AstroNote* **104**, 1 (2024).

45. J. L. Tonry, L. Denneau, A. N. Heinze, B. Stalder, K. W. Smith, S. J. Smartt, C. W. Stubbs, H. J. Weiland, A. Rest, ATLAS: A high-cadence all-sky survey system. *Publ. Astron. Soc. Pac.* **130**, 064505 (2018).

46. J. Zhang, L. Dessart, X. Wang, Q. Zhai, Y. Yang, L. Li, H. Lin, G. Valerin, Y. Cai, Z. Guo, L. Wang, Z. Zhao, Z. Wang, S. Yan, Probing the shock breakout signal of SN 2024ggi from the transformation of early flash spectroscopy. *Astrophys. J. Lett.* **970**, L18 (2024).

47. S. S. Vasylyev, Y. Yang, A. V. Filippenko, K. C. Patra, T. G. Brink, L. Wang, R. Chornock, R. Margutti, E. L. Gates, A. J. Burgasser, P. R. Karpoor, N. LeBaron, E. Softich, C. A. Theissen, E. Wiston, W. Zheng, Early time spectropolarimetry of the aspherical Type II supernova SN 2023ixf. *Astrophys. J. Lett.* **955**, L37 (2023).

48. J. R. Maund, J. C. Wheeler, L. Wang, D. Baade, A. Clocchiatti, F. Patat, P. Höflich, J. Quinn, P. Zelaya, A spectropolarimetric view on the nature of the peculiar Type I SN 2005hk. *Astrophys. J. Lett.* **722**, 1162–1174 (2010).

49. W. V. Jacobson-Galán, K. W. Davis, C. D. Kilpatrick, L. Dessart, R. Margutti, R. Chornock, R. J. Foley, P. Arunachalam, K. Auchettl, C. R. Bom, R. Cartier, D. A. Coulter, G. Dimitriadis, D. Dickinson, M. R. Drout, A. T. Gagliano, C. Gall, B. Garretson, L. Izzo, D. O. Jones, N. LeBaron, H.-Y. Miao, D. Milisavljevic, Y.-C. Pan, A. Rest, C. Rojas-Bravo, A. Santos, H. Sears, B. M. Subrayan, K. Taggart, S. Tinyanont, SN 2024ggi in NGC 3621: Rising ionization in a nearby, circumstellar-material-interacting Type II supernova. *Astrophys. J.* **972**, 177 (2024).

50. D. Xiang, J. Mo, X. Wang, L. Wang, J. Zhang, H. Lin, L. Chen, C. Song, L.-D. Liu, Z. Wang, G. Li, The red supergiant progenitor of Type II supernova 2024ggi. *Astrophys. J. Lett.* **969**, L15 (2024).

51. T. Pessi, R. Cartier, E. Hueichapan, D. de Brito Silva, J. L. Prieto, R. R. Muñoz, G. E. Medina, P. Diaz, T. S. Li, Early emission lines in SN 2024ggi revealed by high-resolution spectroscopy. *Astron. Astrophys.* **688**, L28 (2024).

52. M. Shrestha, K. A. Bostroem, D. J. Sand, G. Hosseinzadeh, J. E. Andrews, Y. Dong, E. Hoang, D. Janzen, J. Pearson, J. E. Jencson, M. J. Lundquist, D. Mehta, A. P. Ravi, N. Meza Retamal, S. Valenti, P. J. Brown, S. W. Jha, C. Macrie, B. Hsu, J. Farah, D. A. Howell, C. McCully, M. Newsome, E. Padilla Gonzalez, C. Pellegrino, G. Terreran, L. Kwok, N. Smith, M. Schwab, A. Martas, R. R. Munoz, G. E. Medina, T. S. Li, P. Diaz, D. Hiramatsu, B. E. Tucker, J. C. Wheeler, X. Wang, Q. Zhai, J. Zhang, A. Gangopadhyay, Y. Yang, C. P. Gutiérrez, Extended shock breakout and early circumstellar interaction in SN 2024ggi. *Astrophys. J. Lett.* **972**, L15 (2024).

53. A. Singh, R. S. Teja, T. J. Moriya, K. Maeda, K. S. Kawabata, M. Tanaka, R. Imazawa, T. Nakaoka, A. Gangopadhyay, M. Yamanaka, V. Swain, D. K. Sahu, G. C. Anupama, B. Kumar, R. M. Anche, Y. Sano, A. Raj, V. K. Agnihotri, V. Bhalerao, D. Bisht, M. S. Bisht, K. Belwal, S. K. Chakrabarti, M. Fujii, T. Nagayama, K. Matsumoto, T. Hamada, M. Kawabata, A. Kumar, R. Kumar, B. K. Malkan, P. Smith, Y. Sakagami, K. Taguchi, N. Tominaga, A.

Watanabe, Unravelling the asphericities in the explosion and multifaceted circumstellar matter of SN 2023ixf. *Astrophys. J.* **975**, 132 (2024).

54. M. Shrestha, S. DeSoto, D. J. Sand, G. G. Williams, J. L. Hoffman, P. S. Smith, C. McCall, J. R. Maund, I. A. Steele, K. Wiersema, J. E. Andrews, N. Smith, C. Bilinski, P. Milne, R. M. Anche, K. A. Bostroem, G. Hosseinzadeh, J. Pearson, D. C. Leonard, B. Hsu, Y. Dong, E. Hoang, D. Janzen, J. E. Jencson, S. W. Jha, M. J. Lundquist, D. Mehta, N. M. Retamal, S. Valenti, J. Farah, D. A. Howell, C. McCully, M. Newsome, E. P. Gonzalez, C. Pellegrino, G. Terreran, Spectropolarimetry of SN 2023ixf reveals both circumstellar material and an aspherical helium core. *Astrophys. J. Lett.* **982**, L32 (2025).

55. S. S. Vasylyev, L. Dessart, Y. Yang, A. V. Filippenko, K. C. Patra, T. G. Brink, L. Wang, R. Chornock, R. Margutti, E. L. Gates, A. J. Burgasser, H. Sears, P. R. Karpoor, N. LeBaron, E. Softich, C. A. Theissen, E. Wiston, W. Zheng, Spectropolarimetrice of SN 2023ixf: An asymmetric explosion in a confined aspherical circumstellar medium. arXiv:2505.03975. (2025).

56. K. Serkowski, D. S. Mathewson, V. L. Ford, Wavelength dependence of interstellar polarization and ratio of total to selective extinction. *Astrophys. J.* **196**, 261–290 (1975).

57. T.-W. Chen, S. Yang, S. Srivastav, T. J. Moriya, S. J. Smartt, S. Rest, A. Rest, H. W. Lin, H.-Y. Miao, Y.-C. Cheng, A. Aryan, C.-Y. Cheng, M. Fraser, L.-C. Huang, M.-H. Lee, C.-H. Lai, Y.-H. Liu, A. Sankar.K, K. W. Smith, H. F. Stevance, Z.-N. Wang, J. P. Anderson, C. R. Angus, T. de Boer, K. Chambers, H.-Y. Duan, N. Erasmus, M. Fulton, H. Gao, J. Herman, W.-J. Hou, H.-Y. Hsiao, M. E. Huber, C.-C. Lin, H.-C. Lin, E. A. Magnier, K. K. Man, T. Moore, C.-C. Ngeow, M. Nicholl, P.-S. Ou, G. Pignata, Y.-C. Shiau, J. S. Sommer, J. L. Tonry, X.-F. Wang, R. Wainscoat, D. R. Young, Y.-T. Yeh, J. Zhang, J. Zhang, Discovery and extensive follow-up of SN 2024ggi, a nearby Type IIP supernova in NGC 3621. *Astrophys. J.* **983**, 86 (2025).

58. E. Waxman, B. Katz, in *Handbook of Supernovae*, A. W. Alsabti, P. Murdin, Eds. (Springer, 2017), p. 967.

59. J. Morag, N. Sapir, E. Waxman, Shock cooling emission from explosions of red supergiants - I. A numerically calibrated analytic model. *Mon. Not. R. Astron. Soc.* **522**, 2764–2776 (2023).

60. M. Tanaka, K. Maeda, P. A. Mazzali, K. S. Kawabata, K. Nomoto, Three-dimensional explosion geometry of stripped-envelope core-collapse supernovae. II. Modeling of polarization. *Astrophys. J.* **837**, 105 (2017).

61. A. Chieffi, I. Domínguez, P. Höflich, M. Limongi, O. Straniero, Theoretical light curves of Type II-P supernovae and applications to cosmology. *Mon. Not. R. Astron. Soc.* **345**, 111–122 (2003).

62. A. A. Lutovinov, A. N. Semena, I. A. Mereminskiy, S. Y. Sazonov, S. V. Molkov, A. Y. Tkachenko, V. A. Arefiev, SRG/ART-XC detects SN2024ggi in X-rays. *Astron. Telegr.* **16586**, 1 (2024).

63. R. Margutti, B. Grefenstette, NuSTAR detection of SN2024ggi at 2 days post discovery. *Astron. Telegr.* **16587**, 1 (2024).

64. J. Zhang, C. K. Li, H. Q. Cheng, Q. Y. Wu, S. M. Jia, Y. Chen, W. W. Cui, H. Feng, J. Guan, D. W. Han, W. Li, C. Z. Liu, F. J. Lu, L. M. Song, J. Wang, J. J. Xu, S. N. Zhang, H. S. Zhao, X. F. Zhao, C. C. Jin, Z. X. Ling, H. Y. Liu, M. J. Liu, Y. Liu, D. Y. Li, H. Sun, W. Yuan, C. Zhang, W. D. Zhang, R. Z. Li, Y. Wang, H. Zhou, K. Nandra, A. Rau, P. Friedrich, N. Meidinger, V. Burwitz, E. Kuulkers, A. Santovincenzo, P. O'Brien, B. Cordier, X. F. Wang, W. X. Li, SN 2024ggi: Detection of X-ray emission by EP-FXT. *Astron. Telegr.* **16588**, 1 (2024).

65. D. C. Leonard, A. V. Filippenko, A. J. Barth, T. Matheson, Evidence for asphericity in the Type IIN supernova SN 1998S. *Astrophys. J.* **536**, 239–254 (2000).

66. L. Dessart, D. C. Leonard, S. S. Vasylyev, D. J. Hillier, Spectropolarimetric modeling of interacting Type II supernovae. Application to early-time observations of SN 1998S. *Astron. Astrophys.* **696**, L12 (2025).

67. E. A. Zimmerman, I. Irani, P. Chen, A. Gal-Yam, S. Schulze, D. A. Perley, J. Sollerman, A. V. Filippenko, T. Shenar, O. Yaron, S. Shahaf, R. J. Bruch, E. O. Ofek, A. De Cia, T. G. Brink, Y. Yang, S. S. Vasylyev, S. Ben Ami, M. Aubert, A. Badash, J. S. Bloom, P. J. Brown, K. De, G. Dimitriadis, C. Fransson, C. Fremling, K. Hinds, A. Horesh, J. P. Johansson, M. M. Kasliwal, S. R. Kulkarni, D. Kushnir, C. Martin, M. Matuzewski, R. C. McGurk, A. A. Miller, J. Morag, J. D. Neil, P. E. Nugent, R. S. Post, N. Z. Prusinski, Y. Qin, A. Raichoor, R. Riddle, M. Rowe, B. Rusholme, I. Sfaradi, K. M. Sjoberg, M. Soumagnac, R. D. Stein, N. L. Strotjohann, J. H. Terwel, T. Wasserman, J. Wise, A. Wold, L. Yan, K. Zhang, The complex circumstellar environment of supernova 2023ixf. *Nature* **627**, 759–762 (2024).

68. G. Hosseinzadeh, J. Farah, M. Shrestha, D. J. Sand, Y. Dong, P. J. Brown, K. A. Bostroem, S. Valenti, S. W. Jha, J. E. Andrews, I. Arcavi, J. Haislip, D. Hiramatsu, E. Hoang, D. A. Howell, D. Janzen, J. E. Jencson, V. Kouprianov, M. Lundquist, C. McCully, N. E. M. Retamal, M. Modjaz, M. Newsome, E. P. Gonzalez, J. Pearson, C. Pellegrino, A. P. Ravi, D. E. Reichart, N. Smith, G. Terreran, J. Vinkó, Shock cooling and possible precursor emission in the early light curve of the Type II SN 2023ixf. *ApJ* **953**, L16 (2023).

69. W. V. Jacobson-Galán, L. Dessart, R. Margutti, R. Chornock, R. J. Foley, C. D. Kilpatrick, D. O. Jones, K. Taggart, C. R. Angus, S. Bhattacharjee, L. A. Braff, D. Brethauer, A. J. Burgasser, F. Cao, C. M. Carlile, K. C. Chambers, D. A. Coulter, E. Dominguez-Ruiz, C. B. Dickinson, T. de Boer, A. Gagliano, C. Gall, H. Gao, E. L. Gates, S. Gomez, M. Guolo, M. R. J. Halford, J. Hjorth, M. E. Huber, M. N. Johnson, P. R. Karpoor, T. Laskar, N. LeBaron, Z. Li, Y. Lin, S. D. Loch, P. D. Lynam, E. A. Magnier, P. Maloney, D. J. Matthews, M. McDonald, H.-Y. Miao, D. Milisavljevic, Y.-C. Pan, S. Pradyumna, C. L. Ransome, J. M. Rees, A. Rest, C. Rojas-Bravo, N. R. Sandford, L. S. Ascencio, S. Sanjaripour, A. Savino, H. Sears, N. Sharei, S. J. Smartt, E. R. Softich, C. A. Theissen, S. Tinyanont, H. Tohfa, V. A. Villar, Q. Wang, R. J. Wainscoat, A. L. Westerling, E. Wiston, M. A. Wozniak, S. K. Yadavalli, Y. Zenati, SN 2023ixf in Messier 101: Photo-ionization of dense, close-in circumstellar material in a nearby Type II supernova. *Astrophys. J.* **954**, L42 (2023).

70. D. Vartanyan, M. S. B. Coleman, A. Burrows, The collapse and three-dimensional explosion of three-dimensional massive-star supernova progenitor models. *Mon. Not. R. Astron. Soc.* **510**, 4689–4705 (2022).

71. J. M. Blondin, A. Mezzacappa, C. DeMarino, Stability of standing accretion shocks, with an eye toward core-collapse supernovae. *Astrophys. J.* **584**, 971–980 (2003).

72. F. Hanke, B. Müller, A. Wongwathanarat, A. Marek, H.-T. Janka, SASI activity in three-dimensional neutrino-hydrodynamics simulations of supernova cores. *Astrophys. J.* **770**, 66 (2013).

73. G. Stockinger, H.-T. Janka, D. Kresse, T. Melson, T. Ertl, M. Gabler, A. Gessner, A. Wongwathanarat, A. Tolstov, S.-C. Leung, K. Nomoto, A. Heger, Three-dimensional models of core-collapse supernovae from low-mass progenitors with implications for Crab. *Mon. Not. R. Astron. Soc.* **496**, 2039–2084 (2020).

74. C. Winteler, R. Käppeli, A. Perego, A. Arcones, N. Vasset, N. Nishimura, M. Liebendörfer, F.-K. Thielemann, Magnetorotationally driven supernovae as the origin of early galaxy *r*-process elements? *Astrophys. J. Lett.* **750**, L22 (2012).

75. P. Mösta, S. Richers, C. D. Ott, R. Haas, A. L. Piro, K. Boydstun, E. Abdikamalov, C. Reisswig, E. Schnetter, Magnetorotational core-collapse supernovae in three dimensions. *Astrophys. J.* **785**, L29 (2014).

76. M. C. Weisskopf, J. J. Hester, A. F. Tennant, R. F. Elsner, N. S. Schulz, H. L. Marshall, M. Karovska, J. S. Nichols, D. A. Swartz, J. J. Kolodziejczak, S. L. O'Dell, Discovery of spatial and spectral structure in the X-ray emission from the Crab Nebula. *Astrophys. J.* **536**, L81–L84 (2000).

77. J. M. Laming, U. Hwang, B. Radics, G. Lekli, E. Takács, The polar regions of Cassiopeia A: The aftermath of a gamma-ray burst? *Astrophys. J.* **644**, 260–273 (2006).

78. D. Milisavljevic, T. Temim, I. De Looze, D. Dickinson, J. M. Laming, R. Fesen, J. C. Raymond, R. G. Arendt, J. Vink, B. Posselt, G. G. Pavlov, O. D. Fox, E. Pinarski, B. Subrayan, J. Schmidt, W. P. Blair, A. Rest, D. Patnaude, B.-C. Koo, J. Rho, S. Orlando, H.-T. Janka, M. Andrews, M. J. Barlow, A. Burrows, R. Chevalier, G. Clayton, C. Fransson, C. Fryer, H. L. Gomez, F. Kirchschlager, J.-J. Lee, M. Matsuura, M. Niculescu-Duvaz, J. D. R. Pierel, P. P. Plucinsky, F. D. Priestley, A. P. Ravi, N. S. Sartorio, F. Schmidt, M. Shahbandeh,

P. Slane, N. Smith, N. Sravan, K. Weil, R. Wesson, J. C. Wheeler, A JWST survey of the supernova remnant Cassiopeia A. *Astrophys. J. Lett.* **965**, L27 (2024).

79. A. I. MacFadyen, S. E. Woosley, Collapsars: Gamma-ray bursts and explosions in "failed supernovae". *Astrophys. J.* **524**, 262–289 (1999).

80. D. Kasen, L. Bildsten, Supernova light curves powered by young magnetars. *Astrophys. J.* **717**, 245–249 (2010).

81. S. E. Woosley, Bright supernovae from magnetar birth. *Astrophys. J.* **719**, L204–L207 (2010).

82. A. Ercolino, H. Jin, N. Langer, L. Dessart, Interacting supernovae from wide massive binary systems. *Astron. Astrophys.* **685**, A58 (2024).

83. S. Li, P. Sanhueza, H. Beuther, H.-R. V. Chen, R. Kuiper, F. A. Olguin, R. E. Pudritz, I. W. Stephens, Q. Zhang, F. Nakamura, X. Lu, R. L. Kuruwita, T. Sakai, T. Henning, K. Taniguchi, F. Li, Observations of high-order multiplicity in a high-mass stellar protocluster. *Nat. Astron.* **8**, 472–481 (2024).

84. R. A. Chevalier, D. Luo, Magnetic shaping of planetary nebulae and other stellar wind bubbles. *Astrophys. J.* **421**, 225 (1994).

85. E. G. Blackman, A. Frank, J. A. Markiel, J. H. Thomas, H. M. Van Horn, Dynamos in asymptotic-giant-branch stars as the origin of magnetic fields shaping planetary nebulae. *Nature* **409**, 485–487 (2001).

86. L. Wang, J. C. Wheeler, P. Höflich, A. Khokhlov, D. Baade, D. Branch, P. Challis, A. V. Filippenko, C. Fransson, P. Garnavich, R. P. Kirshner, P. Lundqvist, R. McCray, N. Panagia, C. S. J. Pun, M. M. Phillips, G. Sonneborn, N. B. Suntzeff, The axisymmetric ejecta of supernova 1987A. *Astrophys. J.* **579**, 671–677 (2002).

87. J. C. Wheeler, I. Yi, P. Höflich, L. Wang, Asymmetric supernovae, pulsars, magnetars, and gamma-ray bursts. *Astrophys. J.* **537**, 810–823 (2000).

88. S. Akiyama, J. C. Wheeler, D. L. Meier, I. Lichtenstadt, The magnetorotational instability in core-collapse supernova explosions. *Astrophys. J.* **584**, 954–970 (2003).

89. J. C. Wheeler, J. R. Maund, S. M. Couch, The shape of Cas A. *Astrophys. J.* **677**, 1091–1099 (2008).

90. F. Patat, J. R. Maund, S. Benetti, M. T. Botticella, E. Cappellaro, A. Harutyunyan, M. Turatto, VLT spectropolarimetry of the optical transient in NGC 300. *Astron. Astrophys.* **510**, A108 (2010).

91. L. Dessart, D. C. Leonard, D. J. Hillier, G. Pignata, Multiepoch VLT-FORS spectropolarimetric observations of supernova 2012aw reveal an asymmetric explosion. *Astron. Astrophys.* **651**, A19 (2021).

92. H. F. Stevance, J. R. Maund, D. Baade, P. Höflich, S. Howerton, F. Patat, M. Rose, J. Spyromilio, J. C. Wheeler, L. Wang, The evolution of the 3D shape of the broad-lined Type Ic SN 2014ad. *Mon. Not. R. Astron. Soc.* **469**, 1897–1911 (2017).

93. Y. Yang, D. Baade, P. Hoeflich, L. Wang, A. Cikota, T.-W. Chen, J. Burke, D. Hiramatsu, C. Pellegrino, D. A. Howell, C. McCully, S. Valenti, S. Schulze, A. Gal-Yam, L. Wang, A. V. Filippenko, K. Maeda, M. Bulla, Y. Yao, J. R. Maund, F. Patat, J. Spyromilio, J. C. Wheeler, A. Rau, L. Hu, W. Li, J. E. Andrews, L. Galbany, D. J. Sand, M. Shahbandeh, E. Y. Hsiao, X. Wang, The interaction of supernova 2018evt with a substantial amount of circumstellar matter - An SN 1997cy-like event. *Mon. Not. R. Astron. Soc.* **519**, 1618–1647 (2022).

94. D. A. Howell, P. Höflich, L. Wang, J. C. Wheeler, Evidence for asphericity in a subluminous Type Ia supernova: Spectropolarimetry of SN 1999by. *Astrophys. J.* **556**, 302–321 (2001).

95. N. Mandarakas, K. Tassis, R. Skalidis, 3D interstellar medium structure challenges the Serkowski relation. *Astron. Astrophys.* **698**, A168 (2025).

96. I. Appenzeller, K. J. Fricke, W. Fürtig, W. Gassler, R. M. Hafner, R. Harke, W. Hummel, P. Jürgens, W. Meisl, B. Muschielok, H. E. Nicklas, G. Rupprecht, W. Seifert, O. Stahl, T.

Szeifert, K. Tarantik, Successful commissioning of FORS1 – The first optical instrument on the VLT. *Messenger* **94**, 1–6 (1998).

97. J. Anderson, Very Large Telescope Paranal Science Operations FORS2 User Manual (European Southern Observatory Doc. No. VLT-MAN-ESO-13100-1543, 2018).

98. D. Tody, in *Instrumentation in Astronomy VI*, D. L. Crawford, Ed. (SPIE, 1986), vol. 627, p. 733.

99. D. Tody, in *Astronomical Data Analysis Software and Systems II*, R. J. Hanisch, R. J. V. Brissenden, J. Barnes, Eds. (Astronomical Society of the Pacific, 1993), vol. 52, p. 173.

100. F. Patat, M. Romaniello, Error analysis for dual-beam optical linear polarimetry. *Publ. Astron. Soc. Pac.* **118**, 146–161 (2006).

101. J. F. L. Simmons, B. G. Stewart, Point and interval estimation of the true unbiased degree of linear polarization in the presence of low signal-to-noise ratios. *Astron. Astrophys.* **142**, 100–106 (1985).

102. Y. Yang, P. Hoeflich, D. Baade, J. R. Maund, L. Wang, P. J. Brown, H. F. Stevance, I. Arcavi, J. Burke, A. Cikota, A. Clocchiatti, A. Gal-Yam, M. L. Graham, D. Hiramatsu, G. Hosseinzadeh, D. A. Howell, S. W. Jha, C. McCully, F. Patat, D. J. Sand, S. Schulze, J. Spyromilio, S. Valenti, J. Vinkó, X. Wang, J. C. Wheeler, O. Yaron, J. Zhang, The young and nearby normal Type Ia supernova 2018gv: UV-optical observations and the earliest spectropolarimetry. *Astrophys. J.* **902**, 46 (2020).

103. A. Cikota, F. Patat, S. Cikota, T. Faran, Linear spectropolarimetry of polarimetric standard stars with VLT/FORS2. arXiv:1610.00722 (astro-ph.IM) (2017).

104. B. S. Koribalski, L. Staveley-Smith, V. A. Kilborn, S. D. Ryder, R. C. Kraan-Korteweg, E. V. Ryan-Weber, R. D. Ekers, H. Jerjen, P. A. Henning, M. E. Putman, M. A. Zwaan, W. J. G. de Blok, M. R. Calabretta, M. J. Disney, R. F. Minchin, R. Bhathal, P. J. Boyce, M. J. Drinkwater, K. C. Freeman, B. K. Gibson, A. J. Green, R. F. Haynes, S. Juraszek, M. J. Kesteven, P. M. Knezek, S. Mader, M. Marquarding, M. Meyer, J. R. Mould, T. Oosterloo,

J. O'Brien, R. M. Price, E. M. Sadler, A. Schröder, I. M. Stewart, F. Stootman, M. Waugh, B. E. Warren, R. L. Webster, A. E. Wright, The 1000 brightest HIPASS galaxies: HI properties. *Astrophys. J.* **128**, 16–46 (2004).

105. N. N. Chugai, Broad emission lines from the opaque electron-scattering environment of SN 1998S. arXiv:astro-ph/0106234 (astro-ph) (2001).

106. L. Dessart, D. J. Hillier, S. Gezari, S. Basa, T. Matheson, SN 1994W: An interacting supernova or two interacting shells? *Mon. Not. R. Astron. Soc.* **394**, 21–37 (2009).

107. C. Huang, R. A. Chevalier, Electron scattering wings on lines in interacting supernovae. *Mon. Not. R. Astron. Soc.* **475**, 1261–1273 (2018).

108. D. Kasen, D. Branch, E. Baron, D. Jeffery, A complete analytic inversion of supernova lines in the Sobolev approximation. *Astrophys. J.* **565**, 380–384 (2002).

109. S. Chandrasekhar, On the radiative equilibrium of a stellar atmosphere. X. *Astrophys. J.* **103**, 351 (1946).

110. D. Branch, D. J. Jeffery, M. Blaylock, K. Hatano, Supernova resonance-scattering profiles in the presence of external illumination. *Publ. Astron. Soc. Pac.* **112**, 217–223 (2000).

111. X. Wen, H. Gao, S. Ai, L.-D. Liu, J.-P. Zhu, W.-H. Lei, Polarization signature of companion-fed supernovae arising from BH-NS/BH progenitor systems. *Astrophys. J.* **955**, 9 (2023).

112. A. D. Code, B. A. Whitney, Polarization from scattering in blobs. *Astrophys. J.* **441**, 400 (1995).

113. P. Hoflich, Asphericity effects in scatterring dominated photospheres. *Astron. Astrophys.* **246**, 481 (1991).

114. L. B. Lucy, Monte Carlo techniques for time-dependent radiative transfer in 3-D supernovae. *Astron. Astrophys.* **429**, 19–30 (2005).

115. M. Bulla, S. A. Sim, M. Kromer, Polarization spectral synthesis for Type Ia supernova explosion models. *Mon. Not. R. Astron. Soc.* **450**, 967–981 (2015).

116. S. Chandrasekhar, *Radiative Transfer* (Dover Publications, 1960).

117. L. Dessart, D. J. Hillier, Quantitative spectroscopy of photospheric-phase type II supernovae. *Astron. Astrophys.* **437**, 667–685 (2005).

118. M. L. McCall, Are supernovae round? I - The case for spectropolarimetry. *Mon. Not. R. Astron. Soc.* **210**, 829–837 (1984).

119. A. Burrows, D. Vartanyan, Core-collapse supernova explosion theory. *Nature* **589**, 29–39 (2021).

120. P. Höflich, A. Khokhlov, L. Wang, in *20th Texas Symposium on Relativistic Astrophysics*, J. C. Wheeler, H. Martel, Eds. (American Institute of Physics, 2001), vol. 586, pp. 459–471.

121. Gaia Collaboration, VizieR Online Data Catalog: Gaia EDR3 (Gaia Collaboration, 2020).

122. J. A. Cardelli, G. C. Clayton, J. S. Mathis, The relationship between infrared, optical, and ultraviolet extinction. *Astrophys. J.* **345**, 245 (1989).

123. E. F. Schlafly, D. P. Finkbeiner, Measuring reddening with Sloan Digital Sky Survey stellar spectra and recalibrating SFD. *Astrophys. J.* **737**, 103 (2011).
